# Eco-Evolutionary Feedbacks and the Maintenance of Metacommunity Diversity in a Changing Environment

**DOI:** 10.3390/genes11121433

**Published:** 2020-11-28

**Authors:** Aidan P. Fielding, Jelena H. Pantel

**Affiliations:** 1 Department of Biology, The College of William and Mary, P.O. Box 8795, Williamsburg, VA 23187-8795, USA; aidanfielding@gmail.com; 2 Department of Computer Science, Mathematics, and Environmental Science, The American University of Paris, 6 rue du Colonel Combes, 75007 Paris, France

**Keywords:** eco-evolutionary dynamics, eco-evolutionary feedback, metacommunity, evolving metacommunity, coexistence, competition, adaptive dynamics, quantitative trait evolution

## Abstract

The presence and strength of resource competition can influence how organisms adaptively respond to environmental change. Selection may thus reflect a balance between two forces, adaptation to an environmental optimum and evolution to avoid strong competition. While this phenomenon has previously been explored in the context of single communities, its implications for eco-evolutionary dynamics at the metacommunity scale are largely unknown. We developed a simulation model for the evolution of a quantitative trait that influences both an organism’s carrying capacity and its intra- and interspecific competitive ability. In the model, multiple species inhabit a three-patch landscape, and we investigated the effect of varying the connectivity level among patches, the presence and pace of directional environmental change, and the strength of competition between the species. Our model produced some patterns previously observed in evolving metacommunity models, such as species sorting and community monopolization. However, we found that species sorting was diminished even at low rates of dispersal and was influenced by competition strength, and that monopolization was observed only when environmental change was very rapid. We also detected an eco-evolutionary feedback loop between local phenotypic evolution at one site and competition at another site, which maintains species diversity in some conditions. The existence of a feedback loop maintained by dispersal indicates that eco-evolutionary dynamics in communities operate at a landscape scale.

## 1. Introduction

Competition for resources is a critical component structuring biodiversity in nature [1,2]. Many ecological theories developed to understand competition, coexistence, and biodiversity treat the traits that influence species interactions as fixed (e.g. [3]). Recent experimental studies have demonstrated, however, that critical parameters, such as the interaction strength of competitors (α) and the minimum resource level required for positive growth (R*), can evolve on an ecological time scale [4,5]. Studies have also demonstrated that ecological competition can either inhibit or enhance evolutionary diversification and speciation, and can play an important role for understanding biodiversity over macroevolutionary time scales [6,7,8]. It is therefore important that theory is developed to predict how coexistence and consequent biodiversity are affected when traits influencing ecological competition can evolve [9,10]. This is especially important as anthropogenic climate change increases existing or introduces novel selection pressures [11].

Theoretical models that evaluate the role of evolution for competition and coexistence in multiple species have been developed at both the local (single-site) and regional (multisite) scale. At the regional scale, de Mazancourt et al. [12] modeled phenotypic evolution in a trait that influences growth (where biomass at a site depends on the similarity between the population’s trait value and the local environmental optimum value) for multiple species that inhabited a patchy landscape and compete via a lottery for recruitment sites in proportion to their biomass. They found that adaptive evolution was slowed down when sites were connected, as local species were prevented from adapting by the arrival of pre-adapted immigrants (the metacommunity process of species sorting; [13,14]). Other multisite studies used a similar approach, where the evolving trait determines fitness in the local environment and species compete via a microsite lottery, and found that resident species can sometimes prevent colonization by other pre-adapted species due to a combination of local adaptation and a numerical advantage (community monopolization: [15,16,17,18]). Norberg et al. [19] modeled competition as per capita impacts of species on one another (via a Lotka–Volterra interaction term), and considered evolution in a trait that influenced growth rates (and was thus decoupled from competition). They found that local adaptation was inhibited by increased dispersal, because species shifted their ranges instead of adapting to environmental change (which actually increased extinctions of species at range and trait extremes in their system). Taken together, these studies indicate that metacommunity composition emerges as a product of environmental heterogeneity, site connectivity (or species dispersal rate), and the speed of adaptive evolution. However, in all of these studies the measure of (relative) fitness (recruitment, survival, or intrinsic rate of population increase) depended only on the local environment and was decoupled from competition.

In nature, trait evolution can be influenced by both abiotic and biotic selection pressures. In zooplankton, for example, evolution of important life history traits such as body size is driven not only by temperature (e.g., [20]), but also by selection for increased grazing efficiency (and thus increased competitive ability) and by predation risk from visually hunting fish [21,22,23]. Traits in protozoans, such as cell size or population growth rate, can also be influenced by competition and predation [24,25]. Traits with evolution that is influenced by both the abiotic environment and ecological competition (i.e., that are under a mixture of stabilizing and frequency-dependent disruptive selection) are frequently assumed in models of diversification and speciation (e.g., [26,27,28]), but usually assume a single-site setting (e.g., sympatric speciation; for exceptions, see, e.g., [29,30]) and constant (external) environmental conditions. The model of Johansson [31] combined trait-dependent competition with selection towards a changing local optimum (2008). The evolving trait influences both fitness in the local environment (i.e., carrying capacity depends on the distance between the trait value and the local optimum trait value) and the per capita effect of other species (i.e., the impact of competition depends on the difference in trait values between species). Competition was found to slow the rate of evolution, as trait convergence on the environmental optimum led to increased competition with other species (and populations of the same species with differing trait values; [31]). However, the model of Osmond and de Mazancourt [32] determined that competition can either decrease or increase the rate of evolution, depending on the degree of niche overlap between competitors, and whether competition selects in the same direction as the new environment or in the opposite direction.

The goal of our study was to consider the effects of ecological competition on trait evolution in response to a variable environment at the regional, metacommunity scale. The combination of environmental heterogeneity and spatial connectivity is expected to produce local communities that vary in the relative strength of selection due to the environment and due to competition. When these communities experience a changing environment, their ability to adapt might thus be either diminished or accelerated. Spatial structure could thus play a critical role in the ability of competing species to avoid extinction and adapt to environmental change (evolutionary rescue [33,34]).

**Figure 1:**
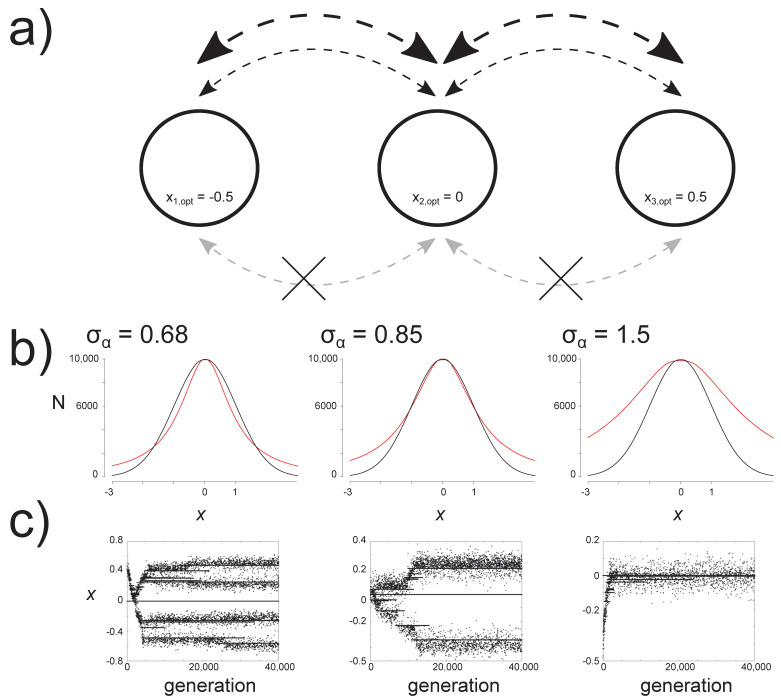
Model of metacommunity eco-evolutionary dynamics for species that compete for resources in a changing environment. (**a**) Communities inhabit a three-patch landscape with sites that vary in their resource distribution (which yields a distribution of carrying capacities centered around a maximum value with an optimal trait value *x_opt_*) and are connected at three levels of dispersal (*d* = 0, 0.01, 0.1 are the grey, thin black, and thick black lines respectively). (**b**) Simulations in the metacommunity were conducted using three different values for competition strength (σ_α_ = 0.68, 0.85, 1.5). Resident communities for the simulations were generated from modeling long-term coevolution in each patch (no dispersal) in a constant environment. The communities that emerge depend on the relationships between two functions that depend on a phenotype *x*. The competition function *C*(*x*) (red line, Equation (3)) depends on the difference in trait values between two populations, with a standard deviation σ_α_. The carrying capacity function *K*(*x*) (black line, Equation (1)) depends on the distance of a population’s trait from the local environmental optimum, can be interpreted as different resources that species can utilize at various efficiencies, and is a distribution with a standard deviation σ_K_ (equal to 1 in simulations). (**c**) The evolutionary history of a set of initial communities used in the simulation are shown. Species can coexist in a stable competitive hierarchy that evolves from a single initial population using a stochastic simulation of adaptive dynamics. In the plot, points represent trait values for all populations present at generation *t* (i.e., at least one individual exists with that trait value, population size is not represented), and a horizontal line at the optimal trait value is shown. The number of species (all unique populations in existence after 10^6^ generations) and the number of distinct cluster of phenotypes are determined by the ratio of the niche width to the width of the resource distribution (σ_α_/σ*_K_*). An initial mutant population can evolve into multiple stable clusters when σ_α_/σ*_K_* < 1 (corresponding to σ_α_ = 0.68 and 0.85 in this model), as a region of trait space exists where new mutants with trait values that differ from the local optimum still have a carrying capacity that exceeds the impact of competition (where the black line is above the red line). When σ_α_/σ*_K_* > 1 (σ_α_ = 1.5 in this model), a single population is maintained with a trait value at the local optimum. In these communities, *x*_0_ = 0.3602, −0.4795, and −0.4335 for σ_α_ = 0.68, 0.85, and 1.5, respectively.

We modeled population growth and competition for multiple species that inhabit a three-patch landscape. Population growth depends on a quantitative trait that determines both the carrying capacity in the local environment and the degree of intra- and interspecific competition. This trait can evolve (implemented using individual-based simulations grounded in the adaptive-dynamics framework [35,36]), and individuals can disperse among patches (Figure 1a). We monitored metacommunity eco-evolutionary dynamics to assess the effects of varying three components of the system: (1) spatial structure (isolated patches and patches with low and high levels of dispersal connectivity), (2) rate of environmental change (none, slow, and fast), and (3) strength of competition as a function of phenotypic distance (Figure 1b). For each scenario, we evaluated evolutionary and community dynamics by considering local and regional species diversity, measures of stability, and distributions of the quantitative trait over time and across sites. Because previous models have already addressed some aspects of these components, we focused specifically on two questions. First, we evaluated whether the overlap between selection from the local environment and from competition was a fixed property at each site or whether dispersal and environmental change caused it to vary over space and time. The degree of overlap between these two selection pressures (referred to as ‘niche overlap’ by Osmond and de Mazancourt [32], i.e., the overlap between the niche of competitors and the niche that a focal population is selected for by the environment) can determine whether adaptive evolution is constrained or increased by competition. In the case of complete niche overlap, the environment selects for a niche that increases the strength of competition, and adaptive evolution is constrained. However, in the case of partial niche overlap, competition decreases as the focal population adapts to the environment, and the speed of adaptive evolution is actually increased. In our model, as in the models of Osmond and de Mazancourt [32] and Johansson [31], the niche width is held as fixed within a simulation; but in Osmond and de Mazancourt [32], the niche overlap is also fixed and in Johansson [32] the niche overlap varies (stochastically) in the scenario of a stochastically varying direction of environmental change. In our model, we evaluate the effects of spatial structure and environmental change for the degree of niche overlap and how it varies over space and time. Second, we evaluated whether the findings of existing studies of evolution in metacommunities differed when trait evolution was influenced both by the environment and by competition. We used our evolving metacommunity model to determine whether the match between species traits and their environment (species sorting [13]) observed at low to intermediate dispersal in previous studies is also observed when competition potentially restricts species from adapting to the local environmental optimum, and under what conditions global monopolization emerged.

## 2. Methods

### 2.1. Overview

We developed a model of quantitative trait evolution in a metacommunity of species that compete for resources and inhabit a three-patch landscape. Patches vary in their environmental properties (which favor an optimum trait value for resident species; Figure 1a). Species in the model are (asexually reproducing) populations with distinct phenotypes that vary along a single trait axis (see below for an exact definition). Our results are thus limited to understanding evolution in asexual populations with mutations of predominantly small effect (for a discussion of why this assumption may not accurately describe phenotypic evolution, and how sexual reproduction can influence competition for continuous resources, see [37]). Our model results will thus be most applicable to systems where a guild of asexually reproducing species respond to environmental selection pressures and compete for resources via a shared key trait such as body or beak size (e.g., [38,39]), or resource uptake rate (e.g., [40]). Population growth follows Lotka–Volterra dynamics with density dependence and intra- and interspecific competition, and evolutionary dynamics are implemented using a simulation based on the adaptive-dynamics framework. This framework is useful to study effects of interactions between species that are connected by dispersal across multiple sites, because it links population dynamics directly to evolutionary dynamics [36,41], and also because these models can produce trait diversification that resembles distributions of species along niche axes observed in natural populations [26,42]. The phenotypic trait determines an individual’s carrying capacity and the strength of competition with other individuals. We model environmental change as a constant rate of increase in the optimum trait value (the trait value that leads to the highest carrying capacity). We consider the same rate of change for all patches. All individuals of all species have a uniform probability of dispersal and can move equiprobably to the other patches in the landscape. The formulas and model details for ecology, evolution, and dispersal are described below in the sections *Within-patch ecology*, *Evolution*, *Extinction*, *Dispersal*, and *Environmental dynamics*. The details for simulation conditions to address our research questions are described in the sections *Metacommunity initialization* and *Simulation conditions*. Finally, we describe measures for quantifying eco-evolutionary properties (e.g., diversity, degree of phenotypic evolution) in the section *Metrics for evolutionary and ecological properties*.

### 2.2. Within-Patch Ecology

Population dynamics are evaluated for all populations *i* that differ in their trait value *x*, i.e., each population has its own trait value *x_i_* and population size *N_it_* that varies over time *t*. Novel phenotypes that arise via mutation are considered new populations. Population size *N_it_* is a function of the vectors of trait values *x* for all populations present in the local patch and their population size in the previous generation *N_jt-1_*. Population growth depends on trait values *x* in two ways. First, the carrying capacity *K* of a population with trait value *x_i_* is a Gaussian function of the distance of *x_i_* from *x_opt_*, which is the patch-specific optimal trait value:

(1) $$K\left(x_{i}\right)=K_{max}\times e^{\frac{-{\left(x_{opt}-x_{i}\right)}^{2}}{2\sigma_{K}{}^{2}}}$$

Here *x_opt_* is the trait value corresponding to the maximum carrying capacity, *K_max_*, and *K_max_* is the same for all populations in the patch. Carrying capacity decreases if population traits are distant from the optimum *x_opt_*, and the width of this carrying capacity distribution is determined by the standard deviation term σ*_k_*. *K*(*x*) can be interpreted as representing a resource distribution (biotic or abiotic) on a given patch, scaled to equal the total population that the resources at any given position on the trait axis can sustain. A population’s trait *x_i_* therefore corresponds to its ability to exploit its most efficiently used resource, and its carrying capacity *K*(*x_i_*) is limited by that resource.

Competition between populations is a symmetric function of the difference in their trait values. Competition, the per capita negative effect of a population with trait *x_j_* on a population with trait *x_i_* (α*_ij_*) is determined by a decaying symmetric function of the distance between trait values *x_i_*−*x_j_*:

(2) $$\alpha \left(x_{i},x_{j}\right)=\frac{1}{1+\frac{{\left(x_{i}-x_{j}\right)}^{2}}{2\sigma_{\alpha }^{2}}}$$

where the strength of competition between individuals with differences in trait values is determined by the standard deviation term σ*_α_* (i.e., the niche width; we subsequently refer to the value of σ*_α_* as the competition strength or as the niche width, as an increased σ*_α_* means that more individuals face some competition; see [31]). When *x_i_* = *x_j_*, the populations are ecologically equivalent and their competitive effect on each other is the same as their intraspecific effect on themselves (α*_ii_* = 1; otherwise 0 ≤ α*_ij_* < 1). The competition function (Equation (2)) has heavier tails than a Gaussian function used for *K*(*x*), which prevents evolutionary equilibria with continuous phenotype distributions (see [37]).

The impact of competition for the growth of a population with trait *i* is given by:

(3) $$C\left(x_{i}\right)=\overset{m}{\underset{j=1}{\sum }}N_{jt}\alpha \left(x_{i},x_{j}\right)$$

where *m* is the total number of populations present in the patch, across all species (more on how species are defined in the section *Metacommunity initialization*). Equations (1)–(3) are incorporated into each population *i*’s growth as:

(4) $$G\left(x_{i},N_{it}\right)=r\left(1-\frac{C\left(x_{i}\right)}{K\left(x_{i}\right)}\right)$$

where *r* is the population’s intrinsic rate of increase and *m* is the total number of populations present in the patch, across all species (more on how species are defined in the section *Metacommunity initialization*). We considered discrete population dynamics, so population size from one time step to the next is:

(5) $$N_{it+1}=N_{it}\times \left(1+G_{it}\right)$$

### 2.3. Evolution

A mutation rate µ gives the probability that an individual born in a population carries a mutation. In our model, existing populations give birth to *rN_it_* individuals every generation. Each mutant born in a population in a given generation (total number of mutants *N_m_* = µ*rN_it_*) is treated as a new population (subtracted from the parent population size, with initial population size equal to 1), and mutants do not encounter competition in the generation they are generated (which allowed for computational tractability of tracking all mutant populations over time; results with competition in the same generation did not qualitatively differ from those presented here). For all populations, mutants are subtracted from all individuals born to a parental population (*rN_it_*) before the non-mutant individuals in the parental population experience competition (i.e., before the terms in the parentheses in Equation (4) are applied). The trait value of the mutant population, *x_m_*, is drawn from a normal distribution centered at the parental trait value *x_i_* with standard deviation σ_µ_, $x_{m}\sim N\left(x_{i},\sigma_{\mu }^{2}\right)$.

### 2.4. Extinction

At the end of each generation *t*, we remove all populations with *N_it_* < 1. We also implemented stochastic extinction at low population size: all populations with *N_it_* below an extinction threshold θ but above 1 have a probability ρ of being removed from the system.

### 2.5. Dispersal

All patches are equally connected by dispersal. Each generation, all individuals can disperse with probability *d*. Dispersing individuals from one patch have an equal probability of migrating to any of the other patches.

### 2.6. Environmental Dynamics

The metacommunity landscape consists of *k* patches (in all of the simulation conditions considered in this study, we set *k* = 3). The optimal trait values *x_opt,k_* are evenly spaced along the trait axis with fixed difference δ, which gives the degree of environmental heterogeneity in the metacommunity. Environmental change was implemented each generation, where the patch optimal trait values *x_opt,k_* increase by the same rate ∆ in all patches.

### 2.7. Metacommunity Initialization

We created initial metacommunities for each of the three levels of niche width (σ_α_ = 0.68, 0.86, 1.5), by seeding *k* = 3 patches with an initial population of size *N*_0_ = 500 and an initial phenotype of *x*_0_ that was allowed to evolve for 10^6^ generations under constant environmental conditions (*x_opt_* = 0, ∆ = 0) and no dispersal (*d* = 0). All patches had a *K_max_* = 10,000; other parameters were σ*_k_* = 1, *r* = 1.9, µ = 10^−5^, and σ_µ_ = 0.05, θ = 2, and ρ = 0.025 (results were qualitatively similar but over longer time scales for lower values of µ and σ_µ_). Preliminary tests of our simulation did not indicate an association between initial trait value and phenotypic trajectories (see *Results, Initial communities* below for detail), so we chose random *x*_0,*k*_ for the initialization. For σ_α_ = 0.68, *x*_0,1_ = 0.1140, *x*_0,2_ = −0.4689, and *x*_0,3_ = 0.3602; for σ_α_ = 0.85, *x*_0,1_ = 0.3511, *x*_0,2_ = −0.4795, and *x*_0,3_ = 0.1379; for σ_α_ = 1.5, *x*_0,1_ = 0.4198, *x*_0,2_ = −0.3504, and *x*_0,3_ = −0.4335. The evolving populations adapted to the local environmental conditions, and for low values of σ_α_, experienced evolutionary branching as a result of selection from competition (Figure 1c). The coexisting lineages with distinct trait values present at the end of the initialization period were considered unique species (similar to [31]). To adjust the communities that emerged from the initialization (with trait values adapted for *x_opt_* = 0) for use in the metacommunity simulation (with local environmental optima of *x*_1,*opt*_ = −0.5, *x*_2,*opt*_ = 0, and *x*_3,*opt*_ = 0.5; i.e., among-patch environmental distance δ = 0.5), we subtracted 0.5 from trait values for all individuals in patch 1 (i.e., *x_i,k_*_=1_ = *x_i_* – 0.5), kept the same trait values for all individuals in patch 2 (*x_i,k_*_=2_ = *x_i_*), and added 0.5 to trait values for all individuals in patch 3 (*x_i,k_*_=1_ = *x_i_* + 0.5). These metacommunities represent stochastic approximations of the coevolutionary equilibrium for that set of model conditions.

### 2.8. Simulation Conditions

The coevolved communities that arose during the initialization were then used as initial communities for the evolving metacommunity simulations. For each of the three different levels of niche width (σ_α_), we ran simulations for each three-patch metacommunity at different dispersal levels (*d* = 0, 0.01, 0.1) and different rates of environmental change (∆ = 0, 10^−5^, 4 × 10^−4^. All of the 27 simulations ran for 50,000 generations, and all simulation parameters were set at *t*_0_. Simulations were conducted in MATLAB R2017a version 9.2.0.556344.

### 2.9. Metrics for Evolutionary and Ecological Properties

**Figure 2:**
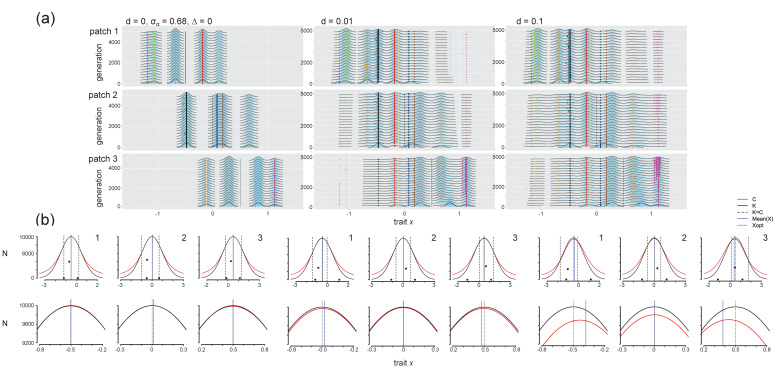
Trait evolution and competition for multiple species in communities over time in an unchanging environment (∆ = 0, σ_α_ = 0.68, *d* = 0, 0.01, and 0.1). (**a**) Time series of phenotypic distributions. Density plots of trait values (*x* axis) weighted by relative abundance (height of density curve; populations with a frequency below 0.01 are not shown) are shown over 5000 generations (*y* axis; the time series was thinned for visualization, so density plots along the *y* axis are shown every 250 generations), with a colored point indicating the species identity of lineages in each generation (each species is assigned a unique color). Simulations were run for 50,000 generations but the dynamics are clear within the first 5000 generations. The solid black line in each plot indicates the local optimal trait value. These density plots show the kernel density estimate of trait values across all individuals in the patch within a generation. Each population with a unique trait value is shown as a point, colored by species identity (drawn using the R package ‘ggplot2’ and ‘ggridges’, with a relative minimum height of 0.01 [43,44]). (**b**) Competition strength and carrying capacity as a function of trait value for *d* = 0, 0.01, and 0.1 (left to right) in three patches (left to right are patch 1–3). Plots on the top row show competition (*C*(*x*), red, Equation (3)) and carrying capacity (*K*(*x*), black, Equation (1)) as a function of trait value *x* (*x* axis) and are calculated as average values from 121 sampled generations spaced evenly from generation 20,000 to 50,000. Black points show the phenotypic distribution in each patch and their corresponding population size (*y* axis). The left and right-most points are the average trait values (across the 121 generations considered from time 20,000 to 50,000) of the species with the lowest and highest trait values and the middle point is the average trait value of the species with the highest population size. The patch’s *x_opt_* (short-dashed black line), the community’s mean *x* (blue solid line, averaged across the sampled 121 time points, weighted by population size), and points where the carrying capacity and impact of competition are equal to one another (long-dashed black line) are shown as well. The plots on the bottom row are zoomed in to better see distinctions between the *C*(*x*) and *K*(*x*) functions.

In our model, we define a species *l* as a population with a distinct trait value (i.e., the points in Figure 1c) that was present at the end of the initialization period (i.e., at time *t*_0_; similar to [31]). No new species are considered as arising after this point. Instead, populations *m* that arise via mutation from a given species *l* at a time *t* > *t*_0_ are considered subpopulations of that species (and so individuals with particular trait values can be indexed as *x_lm_*). We note that a number of species present in a cluster at time *t* = *t*_0_ are rare, and rapidly become extinct when the simulations begin. Therefore, in the subsequent simulations, phenotypic clusters most often represent a single species (e.g., Figure 2a). In some cases, however, two phenotypically similar species survive for a prolonged time period, giving rise to a polymorphic cluster (still treated as distinct species, e.g., the species with the lowest trait values in patch 1 in Figure 2a).

To characterize biodiversity in our metacommunities, we calculated (for each generation) patch α diversity using the inverse of Simpson’s diversity index: ^1^D_α_ = $\frac{1}{\sum_{i=1}^{l}p_{i}{}^{2}}$, where *l* is the number of species present in site *k* and *p_i_* is the relative proportion of the species at that site (all populations *m* for a species *l* are included in this *p_i_* calculation). We calculated regional γ diversity ^1^D_γ_ using the same formula, but after summing population sizes of each species across all sites, and we calculated β diversity as ^1^D_β_ = ^1^${\bar{D}}_{\alpha }$/^1^D_γ_. We calculated the coefficient of variation in total population size within a patch (i.e., across all populations for all species, giving a CV for the total number of individuals in a patch) and the sum of squared deviations of individual trait values from the local environmental optimum (calculated for all individuals *n* within a patch): $SSD_{k}=\sum_{i=1}^{n}{\left(x_{i}-x_{opt}\right)}^{2}$.

To better understand how environment-dependent selection and density-dependent competition (Equations (1)–(3)) interact to structure evolutionary trajectories in our evolving metacommunities, we compared the carrying capacity function *K*(*x*) and the population size change due to competition *C*(*x*), depending on trait value *x* (i.e., the curves in Figure 1b). These curves represent the selection pressures from carrying capacity and competition, respectively (after Figure 5 in [32]). The *C*(*x*) curve, $C_{t}\left(x\right)=\sum_{j=1}^{s}\alpha \left(x,x_{j}\right)N_{jt-1}$ for all populations *s* at time *t* (Equation (3)), where *s* is used to denote all possible values of *x*), gives the total amount of intra- and interspecific competition experienced by a focal individual with trait *x*. We approximated the curve by calculating *C*(*x*) for discrete values of *x* in intervals of 0.001 for the range {*x_opt,k_* − 3, *x_opt,k_* + 3}. In these curves, the value for an existing population’s trait value (i.e., a population actually present in the patch at that time) in generation *t* is calculated including both intraspecific competition (with other individuals with the same trait value) and interspecific competition (with individuals with all other trait values, across the same and other species) experienced in generation *t*, and for all other *x* values in the range it is calculated as the total amount of competition with individuals that have differing trait values. The *K*(*x*) curve was calculated using Equation (1) for the same range of *x* values. Mutants with trait values in a region of the trait axis where *K*(*x*) > *C*(*x*) experience positive population growth, whereas those with *K*(*x*) < *C*(*x*) experience negative growth (Equation (5)). All metrics were calculated and figures were made using R version 3.5.0 [45].

## 3. Results

### 3.1. Initial Communities

Our model is a stochastic simulation of an adaptive dynamics model [35,36], which has been shown in some conditions to exhibit evolutionary branching (where populations with multiple trait values can co-exist despite the presence of a single phenotypic optimum [27,46]). Since competition in our model is symmetric [31,47], the number of populations that can stably co-exist is determined by the ratio between niche width and the width of the resource landscape (σ_α_/σ*_K_*; Figure 1b,c). Evolutionary dynamics and consequent species composition in our model are thus determined by the position of both symmetric functions *α*(*x*) and *K*(*x*) on the resource optimum *x_opt_*, and also the shape of the functional form for *α*(*x*).

We evaluated the impacts of competition for population size using the function *C*(*x*), which gives the total, density-dependent effects of competition for a population. When niche width is smaller than the width of the resource distribution (σ_α_ < σ*_K_*) and the resident population is adapted to the resource optimum (*x_i_* = *x_opt_*), there are regions of trait space where *K*(*x*) exceeds *C*(*x*), and thus mutants with trait values away from the optimum can have positive population growth. In contrast, when σ_α_ > σ*_K_* no mutants can invade, as the reduction in competition is insufficient to compensate for the reduced carrying capacity [26,45] (Figure 1b). In all cases, the evolutionarily stable configuration of species is characterized by a balance between two opposing selective forces: one for increased carrying capacity and one for decreased competition. As niche width decreases relative to the width of the resource landscape, the number of evolutionarily stable coexisting populations and the variance of those populations in trait space both increase. We varied niche width in our simulations using σ_α_ = 0.68, 0.85, and 1.5 (with σ*_K_* = 1), which respectively produces 4, 2, and 1 distinct branches (clusters of populations) as evolutionarily stable solutions in trait space [31] (Figure 1c).

Our model broadly reflected these patterns (approximate number of stable branches) expected under adaptive dynamics, but had some variabilities in the exact configurations of initial communities (i.e., after 10^6^ generations of evolution). The positions of phenotypic clusters were not symmetric around the local *x_opt_*, and these positions also varied among repetitions of initialization runs (examples of repetitions at different *x*_0_ values are shown in Supplementary Information, Figure S1; note that the position of branches did not depend on the choice of *x*_0_). This may result from the stochasticity of mutations, and also because the fitness landscape flattens (and evolution thus becomes very slow) as the populations diversify to exploit the resource landscape. Additionally, the number of distinct clusters was not always constant, but instead demonstrated likely metastability, where differing numbers of clusters could persist for very long periods of time before finally shifting closer towards the predicted evolutionarily stable number. The number of clusters in our simulations always stabilized after 10^6^ generations; but in some instances, these metastable configurations contained clusters that consisted of two populations with similar trait values (e.g., patch 1, Figure 2a and Figure S1; note these are considered distinct species).

### 3.2. Effect of Dispersal in the Absence of Environmental Change

To determine how dispersal in a landscape where the environment is spatially heterogeneous (δ = 0.5) but temporally homogeneous (∆ = 0) influences metacommunity eco-evolutionary dynamics, we evaluated phenotypic distributions and species composition over time when dispersal *d* = 0, 0.01, and 0.1 for *t* = 50,000 generations. We first consider the case of σ_α_ = 0.68 (the most narrow niche width), which allows for coexistence of the largest number of species. Results to address the effects of varying ∆ are discussed in section *Environmental change* and of varying σ_α_ in the section *Increasing niche width*.

#### 3.2.1. Selection Pressures from Carrying Capacity and Competition

In the absence of dispersal (*d* = 0), no significant evolutionary change occurs after the establishment of the initial metacommunity (Figure 2a, left column). As a result, the selection pressure from carrying capacity (*K*(*x*), the black curves in Figure 2b) does not change over time. It also does not vary among patches, with the exception of the location of the optimum trait value *x_opt_*. Dispersal introduces new species into local communities (Figure 2a, middle and right column), and these immigrants alter the selection pressure from competition. Immigrants add density in the distribution of competition effects *C*(*x*) to their region of trait space, and the selection pressure from competition thus varies among patches. In patch 1, immigrants tend to have higher trait values than residents and the selection pressure from competition thus increases towards the right of the distribution. This reduces the impacts of competition towards the left of the distribution, which can be seen in patch 1 in Figure 2b when *d* > 0. The opposite is observed in patch 3, where immigrants tend to have lower trait values than residents, add density to the left of the trait distribution, and reduce the selection pressure from competition towards the right of the distribution (Figure 2b). The region of trait space in a patch where mutants can have positive realized growth rates (*x_fit_*, which we define as the region where *K* > *C*) can thus vary among patches, and the width of this region increases with dispersal. Populations with traits in these higher-fitness regions are selected to be maintained (i.e., populations with trait values in the region where *K* − *C* was highest had the highest population sizes; these values approximate the expected trait equilibrium indicated in [32]; Figure 2b). Dispersal thus shifts the selection pressure from competition in a patch-dependent way, and increasing dispersal increases the width (the range of trait values *x_fit_*) and depth (*K*(*x_fit_*) − *C*(*x_fit_*)) of the positive growth region.

#### 3.2.2. Phenotypic Trajectories and Metacommunity Dynamics

Dispersal slightly increases phenotypic homogenization towards the overall metacommunity ${\bar{x}}_{opt}$ (i.e., small populations with higher trait values colonize and persist in patch 1 and with lower trait values in patch 3, although these immigrant populations are at low density; Figure 2a, *d* > 0) and increases local community phenotypic variance (the distance between the minimum and maximum trait value present in a patch; Figure 2a).

**Figure 3:**
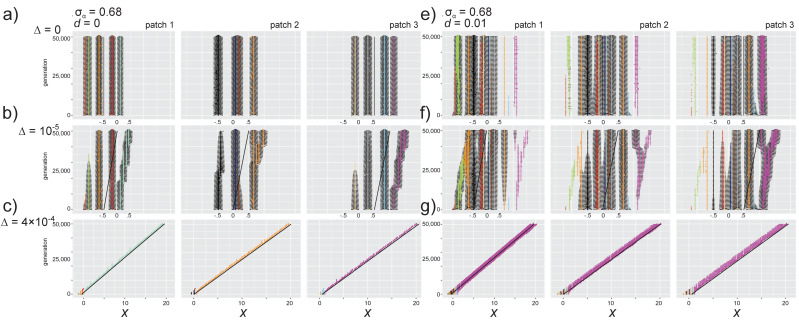
Trait distributions in communities over time across a range of environmental change rates with no and low dispersal. In all plots, σ_α_ = 0.68, *d* = 0 or *d* = 0.01, and the rate of environmental change varies: (**a**,**e**) ∆ = 0, (**b**,**f**) ∆ = 10^−5^, and (**c**,**g**) ∆ = 4 × 10^−4^. The *x* axis range differs for (**c**,**g**), as traits span a wider range (see Figure S4 to compare width of phenotypic distributions across all three rates of environmental change for *d* = 0). Communities in patches 1–3 are shown (from left to right) and features of the trait density plots are as described in Figure 2a (except that the time series are thinned to show every 800 generations and a relative minimum height of 0.05 is used). In (**b**), the species with the highest trait value in each patch is less constrained by competition than other species and experiences the most adaptive trait change. Species with lower trait values are gradually lost to extinction. In (**c**), all species are lost within 5000 generations and the species with the initially highest trait values persists and adapts over time. In (**f**), species can disperse to other patches and the species with the highest trait value in the entire metacommunity experiences the most adaptive trait change. The species in the metacommunity with the lowest trait value also experiences adaptive evolution and increased genetic diversity, but is eventually lost to extinction.

Introducing dispersal led to species replacement and altered local community composition (Figure 2a). In each patch, at least one resident species was replaced by an immigrant species. In patch 1, when *d* = 0.01, an immigrant species from patch 2 with a trait very close to the local resource optimum (*x*_opt_ = −0.5; the species colored black) establishes, as does a species from patch 3 (colored light yellow), a resident species in patch 1 (colored red) is eventually lost to extinction, and there is a strong reduction in population size of the patch 1 resident with the highest trait value (colored light blue; extinctions can be observed in Figure 3a,e, Figures S2 and S3, which show phenotypic distributions for 50,000 generations). In patch 2, two resident species with traits closest to the local environmental optimum (*x*_opt_ = 0; species colored blue and brown) are either lost or greatly reduced in population size as immigrants from patch 1 (colored red) and 3 (colored grey) successfully establish. In patch 3, the species combinations change as two resident species (colored light orange and light blue) are replaced by immigrants from patch 1 (colored red) and 2 (colored orange). The precise trait values of the novel species combinations are difficult to predict, but they differ from the combinations observed to be metastable after the 10^6^ generations of the initialization step. This indicates that the change in selection pressure introduced by dispersal of competitors favors species and trait combinations that differ from those observed when sites are isolated (Figure 2b). With a higher colonization rate (*d* = 0.1), the selection pressures (captured by the *K*(*x*) and *C*(*x*) curves in Figure 2b) shift even further, and there is a wider region of trait space where populations have positive growth (Figure 2b). As a result, some species that were lost to extinction at the lower dispersal level are now retained (e.g., the species colored red in patch 1, the species colored brown in patch 2, and the species colored light blue in patch 3; Figure 2, Figure 3a, Figures S2 and S3).

The loss of or a reduction in resident species following dispersal leads to a decrease in regional γ diversity, whereas immigration causes local α diversity to increase, converging to regional γ diversity (Figure S5). Diversity is higher at *d* = 0.1 compared to *d* = 0.01. As dispersal increases the width of the region of trait space with positive population size (Figure 2b), more mutants are maintained with increasing dispersal and so intraspecific diversity increases with dispersal (Figure S5). Some species with trait values very similar to other species (i.e., the species colored brown and light blue in Figure S2) were lost to extinction, which reduced the competition for mutants of other species (i.e., the species colored green and magenta in Figure S2). However, in the absence of environmental change, where most species are pre-adapted to their environment, broad shifts in community trait composition are primarily due to species replacements and there are fewer shifts in a given species trait distribution. Dispersal leads to slight increases in SSD, as immigration pressure allows more species to persist in different patches, including those distant from the local trait optimum. The patches experience more shifts in population size with dispersal (and thus increasing CV), as the shifted impact of selection pressures leads to gradual reductions in some populations and increases in others (Figure S5).

### 3.3. Effects of Directional Environmental Change in the Absence of Dispersal

#### 3.3.1. Selection Pressures from Carrying Capacity and Competition

To understand the effects of environmental change, we first evaluated simulation results for *d* = 0 (no dispersal), σ_α_ = 0.68, and δ = 0.5 with varying levels of environmental change ∆ = 0, 10^−5^, and 4.4 × 10^−4^. Directional environmental change (increased *x_opt_* each generation) shifts the carrying capacity curve *K*(*x*) to the right at all sites, selecting for individuals with higher trait values (Figure S6). Over time, mutants with trait values higher than the rest of the community experience minimal competition, which increases the overall selection for increasingly higher trait values as environmental selection and selection for reduced competition both favor traits in the same direction. This partial niche overlap—where competition is decreased in the direction selected for by the environment—reflects the partial niche overlap described in [32] (see their Figure 6a), where the speed of evolution is actually enhanced by competition.

#### 3.3.2. Phenotypic Trajectories and Metacommunity Dynamics

In our simulations, the addition of relatively slow environmental change (*d* = 0, ∆ = 10^−5^) caused phenotypic distributions to have gradually increasing trait values over time (Figure 3a,b). Species with the lowest trait value in a patch persisted at an increasingly reduced carrying capacity until they were no longer viable in the changing environment and went extinct, as mutants in these populations with increased trait values experience intense competition from other species with similar trait values. Adaptation thus primarily occurred in the species with the highest trait value (Figure 3b, colored magenta, orange, and light green), as most of the other species in a patch continue to experience increased competition in the direction of environmental selection). Gradual environmental change thus led to an overall decrease in species diversity due to extinctions, but an increase in intraspecific diversity (seen by comparing the left column in Figure 3b,f, Figures S5 and S7). The patches showed two distinct mechanisms for intraspecific diversification. In patch 2, the species with the highest trait value experienced evolutionary branching, where two distinct phenotypes are favored and had relatively high densities (Figure 3b, middle column). In patch 1 and 3, mutants with higher trait values gradually replaced mutant lineages with lower trait value, in a process that continued to favor one distinct trait value over time (Figure 3b, left and right column). This occurs because the distance to the species with the next highest trait value in patch 2 increases after the most similar species becomes extinct (the species colored brown in Figure 3b). Slow environmental change had no strong effects on SSD (compared to no environmental change; Figures S5 and S7). The within-patch and metacommunity CV increased compared to when *d* = 0, which reflects the contrast in population sizes between the adapting species with the highest trait value and the other species with increasingly maladapted traits that are eventually lost to extinction (Figures S5 and S7).

**Figure 4:**
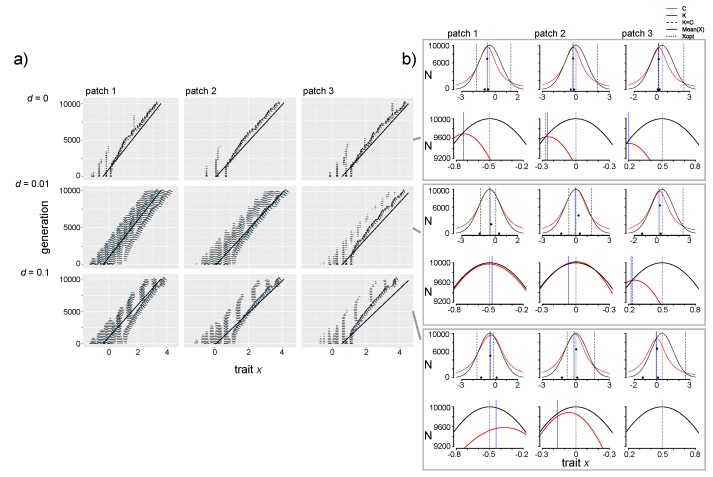
Trait evolution and competition for multispecies communities over time in a rapidly changing environment (∆ = 4 × 10^−4^, σ_α_ = 0.68, *d* = 0, 0.01, and 0.1). (**a**) Distributions of phenotypes in populations and communities over 10,000 generations of the evolving metacommunity simulation (for evolution over 50,000 generations, see Figure 3, Figures S6 and S7, third row). At all levels of dispersal, all but a single species went extinct within ~6000 generations. When *d* > 0, competition with new mutant populations leads to periodic selection for divergent trait values but these are then lost to extinction as the environment continues to change. This intraspecific diversity is always highest in patch 1, where selection for increasing traits is driven by environmental change but immigration from the other patches creates increased selection pressure for lower competition strength and thus lower trait values. (**b**) Competition strength (*C*(*x*), red line, Equation (3) and carrying capacity (*K*(*x*), black line, Equation (1) as a function of trait values (*x* axis), calculated as average values from 121 sampled generations spaced evenly from generation 20,000 to 50,000. Without dispersal, the changing environment selects for individuals with higher trait values in all patches. When patches are linked by dispersal, immigrants are introduced and in patch 1, these increase the fitness of individuals with lower trait values that experience less competition than individuals with higher trait values. These mutant populations arise periodically then become extinct as the environment continues to change. Features of all plots are as described in Figure 2.

With rapid environmental change (*d* = 0, ∆ = 4.4 × 10^−4^), all species in each patch except that with the highest trait value went extinct relatively quickly (Figure 3c and Figure 4a, top row). As was observed by Johansson [31], the single remaining species in each patch can track the changing environment and the mean trait value lags behind the optimum (e.g., Figure 4a; this is similar to models without competition, e.g., [48]; see [49] for a review of quantitative trait evolution in response to environmental change). Though interspecific diversity is lost (Figure S8), intraspecific diversity for the remaining species is higher than with slow environmental change (Figures S4 and S9, left column). This is because selection for increased trait values and decreased competition combine to create a large region of positive carrying capacity (where *K* > *C*), and many mutants with very different trait values can persist (Figure S6b and Figure 4b, top right panel). As the rate of environmental change increases from slow to fast, the overall metacommunity SSD decreases and metacommunity CV increases (because the numerous surviving mutants vary in their population size as the environment continues to change; Figures S10 and S11).

### 3.4. Effects of Dispersal and Directional Environmental Change

#### 3.4.1. Selection Pressures from Carrying Capacity and Competition

*Slow environmental change*: In the full model with dispersal and slow environmental change (∆ = 10^−5^, *d* = 0.01 and *d* = 0.1), evolutionary trajectories result from the interacting effects of a shift in the resource optimum and alteration of the fitness associated with traits due to introduction of immigrant competitors (Figure S12). Environmental change selects for individuals with higher trait values in all patches as the *K*(*x*) curve shifts to the right, while dispersal introduces competitors that increase selection against traits in the direction of the regional mean ${\bar{x}}_{opt}$ (e.g., against higher trait values in patch 1 and against lower trait values in patch 3; Figure S12). The patch-specific shift in *C*(*x*) plays a strong role in determining subsequent eco-evolutionary dynamics. In patch 1, the environment selects for individuals with higher trait values, while immigrants with high trait values from patch 2 and 3 lead to reduced competition and increased selection for individuals with lower trait values. The resulting region of trait space where *K* > *C* thus varies as a result of dispersal rate and speed of evolutionary change. Thus, with slow environmental change, in patch 1 the phenotypic region of positive growth (*x_fit_*) is shifted towards lower trait values compared to the other two patches (Figure S12). In patch 3, directional environmental trait selection and selection for reduced competition are in the same direction, favoring evolution of increasing trait values (Figure 3, Figure S3 and S12). The mean of the phenotypic distribution in patch 2 (${\bar{x}}_{2}$) is similar to the patch optimum *x_2,opt_*, indicating that species in the metacommunity are able to track the slow environmental change.

*Fast environmental change*: When environmental change is more rapid (∆ = 4 × 10^−4^), among-patch variation in the selection pressures increases with increasing dispersal (Figure 4b). When dispersal is low (*d* = 0.01, Figure 4b, middle row), in patch 1, environmental selection for individuals with increased trait values differs from the direction of selection for individuals with lower trait values and decreased competition, leading to a wide range of species with positive carrying capacity. In patch 3, selection towards increased trait values favored by the changing environment is increased further by the reduced competition these individuals experience (Figure 4b). At the highest level of dispersal, patch 1 selects more strongly for individuals with decreased trait values, while patches 2 and 3 experience selection for increased trait values (Figure 4b, bottom row; Figure S13).

This among-site variation in the direction of selection creates a dynamic feedback loop between evolutionary dynamics in Patch 3 and ecological dynamics in patch 1—the increasing environmental optimum trait value selects for individuals with increasingly higher trait values in patch 3, and these individuals immigrate into patch 1, where they increase competition for individuals with high trait values, which in turn creates selection in favor of individuals with lower trait values. These individuals then immigrate into patch 3 and combine with environmental change to increase the strength of selection for individuals with higher trait values in that patch (Figure 4b and Figure S13). As a consequence of this feedback loop, the population in patch 1 is more diverse than the population in patch 3—patch 3 selects for individuals with increasingly higher trait values, while in patch 1 the local population experiences evolutionary branching and populations with divergent trait values are sustained (and then become extinct as the environment continues to change; Figure 4a, bottom row).

#### 3.4.2. Phenotypic Trajectories and Metacommunity Dynamics

*Slow environmental change*: Observed trajectories of evolutionary and ecological properties reflect that fitness landscapes vary in time and space due to the combination of directional environmental selection and competition. With slow environmental change in the absence of dispersal (∆ = 10^−5^, *d* = 0), adaptive trait shifts in each patch primarily occurred in the resident species with the highest trait value, whereas species with low trait values declined to extinction (Figure 3b). Dispersal alters this pattern, as the increased selection for higher trait values in patch 3 creates immigrants that inhibit adaptive directional trait evolution in resident species with high trait values in patch 1 and 2 (Figure 3b,f). As a consequence, only the species with the highest trait value in the metacommunity (which resides in patch 3) shows significant adaptive evolution (and even evolutionary branching). Immigration of this species into the other patches also increases selection for individuals with lower trait values (i.e., in patch 1 and 2; and thus resident species show less of a match to the local environmental optima). In contrast to the results without dispersal, the species with the lowest trait value (i.e., the species colored green in Figure 3f, patch 1) also shows adaptive evolution for a long period of time. However, this species becomes extinct when its trait becomes similar to the species with the next higher trait value (colored orange in Figure 3f). As a result, species γ diversity is not lost with increasing dispersal levels as is the case when ∆ = 0 (Figure S8). At the highest dispersal level, adaptive directional trait evolution is similarly limited to the species with the highest trait value in patch 3, but a diverse species assemblage persists for the 50,000 generations considered here (Figure S3). SSD and CV both increased with increasing dispersal (Figures S10 and S11).

*Fast environmental change*: Dynamics under rapid environmental change (*d* > 0, ∆ = 4 × 10^−4^) differed substantially. Species diversity is rapidly lost (Figure 3g, Figure 4a and Figure S3). Only the species in the metacommunity with the highest trait value adapts, as novel mutants from other species experience increased competition and are lost to extinction. Due to dispersal, patch 3 immigrants colonize patches 1 and 2 and their competition shifts selection towards favoring individuals with lower trait values in these patches (the eco-evolutionary feedback loop described previously). However, selection against these species by the increasing environmental optimum is still stronger than for reduced competition (Figure 4b) and species with lower trait values are lost to extinction within ~5000 generations (Figure 3g, Figure 4a and Figure S3). The domination of the entire metacommunity by one species reflects a pattern of metacommunity monopolization.

The very broad region in trait space where *K* > *C* creates particular evolutionary dynamics (Figure 4b). Individuals with a broad range of trait values can persist for long periods of time, until they are outside the survivable range of *K* > *C* and become extinct. As a result, although a single species from patch 3 monopolizes the entire metacommunity (Figure S8), diverse lineages persist in patches 1 and 2 and maintain an intraspecific competitive hierarchy (Figure 4a and Figure S9). This is observed most strongly at the intermediate dispersal level (middle row of Figure 4a). With higher dispersal, competition and directional environmental change combine for a strong selection pressure favoring higher trait values in patch 3, and the monopolizing species diminishes the maintenance of genetic diversity in the other patches (though some novel lineages that are favored for transient periods of time with reduced competition develop in patches 1 and 2; Figure 4a, Figures S3 and S9). SSD increased with dispersal and its variance over time increased with rapid environmental change (Figure S10). Rapid environmental change led to strong among-patch variation in CV (Figure S11).

#### 3.4.3. Increasing Niche Width

**Figure 5:**
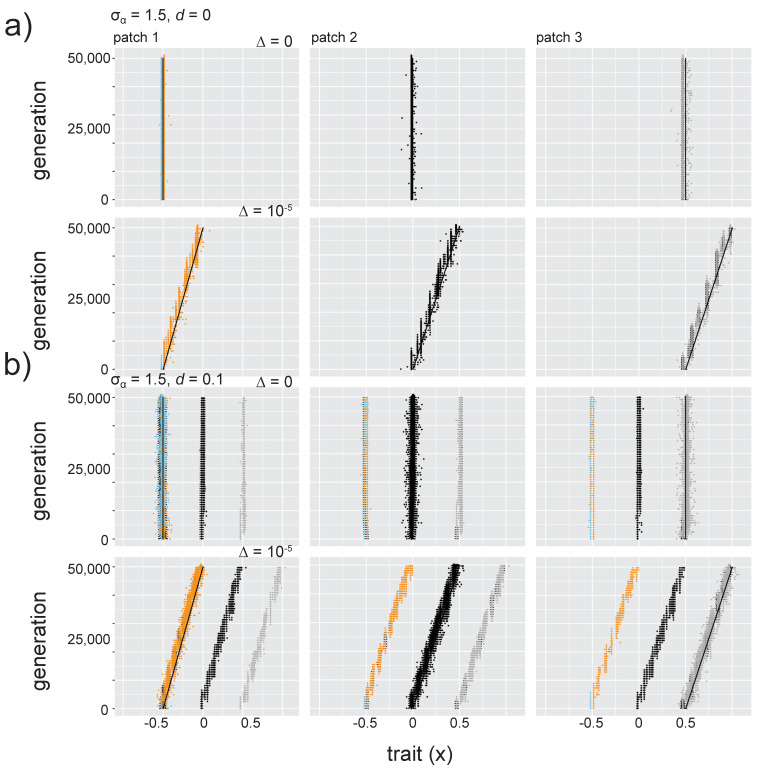
Impacts of competition strength (σ_α_ = 1.5), dispersal rate, and speed of environmental change for community trait distributions over time. Plots give phenotypic distributions (*x* axis is trait value, height of density plots is relative abundance of individuals with that trait value, *y* axis is time; the time series are thinned to show every 800 generations) and the solid black line in each plot indicates the local optimum trait value over time. (**a**) When niche width is high (σ_α_ = 1.5), in the absence of dispersal (*d* = 0) fewer species exist because individuals with similar trait values face strong competition. These species can adapt to slow environmental change (upper row: ∆ = 0; lower row: ∆ = 10^−5^) but retain little intraspecific diversity. (**b**) Dispersal (*d* = 0.1; σ_α_ = 1.5; upper row: ∆ = 0; lower row: ∆ = 10^−5^) not only introduces more interspecific diversity (as species from other patches have distinct enough trait values that they can persist in other patches but at lower population sizes), it also increases the amount of intraspecific diversity, as competition with other species increases the range of viable mutants that can persist with positive population size away from the local environmental optimum.

Increasing niche width (σ_α_ = 0.85, 1.5) means that mutants with increasingly divergent trait values experience stronger competition than when σ_α_ = 0.68 (Figure 1b). As a result, fewer species coexist in a patch: two species at relatively high abundance and one at low abundance in the absence of dispersal when σ_α_^2^ = 0.85, and only one to two (the second at low abundance) species, per patch can coexist for σ_α_^2^ = 1.5 (see *d* = ∆ = 0; Figure 5a, Figures S14–S17 and S18–S21). However, dispersal introduces individuals with very different trait values and as a result the curves depicting selection pressures (of *K*(*x*) and *C*(*x*)) have a wider area for more diverse mutants to persist (the *x_fit_* region; Figure 5b and Figure S22), meaning that intraspecific diversity for resident species is higher when *d* > 0 (i.e., increased width of the phenotypic distribution for resident species; Figure 5a,b, Figures S23 and S24). As was observed for σ_α_ = 0.68, intraspecific diversity was highest at the intermediate dispersal level (*d* = 0.01), caused by the wide range of trait values with positive growth rate (Figure S22). Patches had highest local α diversity at the highest dispersal level (Figures S17 and S21).

With the intermediate niche width (σ_α_ = 0.85), slow environmental change (∆ = 10^−5^) caused species with similar trait values to be lost to extinction, but after that, both remaining species were able to adaptively track environmental change (Figure S14). There was enough phenotypic space between the two remaining species that the species in each patch with the highest trait value was able to undergo branching, where a population with trait values lower than the adaptive optimum but with reduced competition intensity developed and persisted (Figures S25 and S14). However, when dispersal was introduced (*d* > 0, ∆ = 10^−5^), immigrants with higher trait values colonized each patch and inhibited adaptive diversification of both of the resident species (Figures S15 and S16). Despite this inhibition of evolution towards the environmental optimum, however, the species with the lowest trait value in patch 1 maintained genetically diverse populations when *d* = 0.01, ∆ = 10^−5^ (seen in the width of the phenotypic distribution for species colored orange in Figure S15). This resulted because the combination of selection pressures for competition from immigrants and for directional environmental change left a very wide region of trait space with positive carrying capacity values (Figure S25). At the fast speed of environmental change (∆ = 4 × 10^−4^), species diversity was again reduced to a single species (which maintains high genetic diversity, with the highest level at intermediate dispersal level; Figures S14–S16 and S23; for SSD, see Figure S26; for CV, see Figure S27).

Dynamics differed when σ_α_ = 1.5 because fewer species were able to coexist in the system. However, some similar general patterns were observed: environmental change and dispersal introduced increased genetic diversity, and the species were able to track the adaptive optimal trait value. This tracking of the environmental optimum was uninhibited by interspecific competition (Figure 5 and Figures S18–S20; for SSD, see Figure S28; for CV, see Figure S29).

## 4. Discussion

### 4.1. Overview

Species that inhabit changing environments do not live in isolation. Interactions with other species can alter adaptive trajectories [50], and phenotypic traits that influence fitness in the context of the abiotic environment can also be influenced by biotic interactions [51]. Incorporating phenotypic evolution, species interactions, and dispersal in spatially variable landscapes are critical components of models needed to predict how biodiversity will respond to environmental change in the Anthropocene [52]. We used a simulation model to evaluate how competition and spatial structure influence species responses to directional environmental change. We found three main results. First, dispersal among heterogeneous patches introduced among-site variation in the magnitude and direction of selection. In some sites trait evolution towards the adaptive optimum (i.e., the resource maximum) was accelerated as selection from the directionally changing environment and from competition worked in the same direction, while at other sites competition and local environmental adaptation favored different trait values. Second, metacommunity patterns such as species sorting (the match between species traits and the local environment) and monopolization (where diversity is lost and one genotype or species dominates the landscape) differ from those observed in previous models that do not treat phenotype fitness as a function of both the local environment and competition with other individuals. Species sorting can be diminished even at a low dispersal rate, as selection favors species with lower carrying capacity but reduced competition, meaning that not all species will evolve towards the environmental optimum trait value. Species sorting also depended on the strength of intra- and interspecific competition. Monopolization occurs at a fast rate of environmental change, but intraspecific phenotypic diversity was maintained even in this situation and thus genotypic monopolization was not observed. Third, we observed an eco-evolutionary feedback loop between local phenotypic evolution at one site and competition at another site. This feedback loop was maintained by dispersal and indicates that eco-evolutionary dynamics in communities operate at a landscape scale.

### 4.2. Dynamic Variation in Selection Pressures over Time and Space

Our results at the local scale reflect the findings of two previous studies that modeled trait evolution and competition using an adaptive-dynamics framework, and builds on them by considering evolution and competition in a spatially structured landscape. Johansson [31] showed that multiple competing species are retained under slow environmental change and that species are increasingly lost to extinction under fast environmental change. Our model confirms these results (Figure 3). We also show that the selection pressure due to rapid environmental change selects for individuals with increasingly higher trait values (and therefore species with lower trait values are lost even though they experience decreased competition; Figure 4, *d* = 0). By introducing dispersal among patches, we found that persistent immigration of competitors also shifts the fitness landscape, in a way that increases selection for individuals with low trait values in patch 1 and high trait values in patch 3 (e.g., Figure 4). The evolutionary dynamics observed in the presence of both dispersal and environmental change are therefore a product of variation in the fitness landscape and selection pressures over time and space. Osmond and de Mazancourt [32] showed how variation in the shapes and positions of the carrying capacity function *K*(*x*) and the total competition function *C*(*x*) determines the speed and direction of phenotypic evolution. Our results simply introduce spatial structure as one mechanism by which the direction and magnitude of selection varies among populations. In our model, the competition selection curve is not fixed but frequency-dependent, and therefore shifts in response to immigrant individuals with different trait values (Figure S13). Our study presents a first step towards characterizing how landscape features and connectivity patterns can drive eco-evolutionary dynamics in multispecies communities.

### 4.3. Metacommunity Patterns for Trait and Species Diversity

Previous theoretical studies have considered how environmental variation influences trait evolution in communities of competitors in a spatially explicit landscape. In these studies, phenotypic traits were assumed to evolve in response to environmental variation (among sites: [12,16,17,18]; across sites and over time: [19]). Species assemblages emerged as a result of environmental heterogeneity and site connectivity. Maladaptation (a mismatch between species traits and the local environment) could result from spatial mass effects, low levels of genetic variance reducing adaptive evolution, or combinations of dispersal and amounts of genetic variation (i.e., the ‘global monopolization’ of one or a few species due to rapid adaptation or evolutionary priority effects). Our model differs from these studies by considering that competition depends on the phenotypic distance between interacting individuals (using competition coefficients α*_ij_* instead of lottery competition for microsites within patches) and by modeling evolution in a phenotypic trait that influences both local carrying capacity and strength of competition (instead of in a trait that influences only a component of population dynamics such as recruitment, survival, or growth). Considering evolution of traits that reflect fitness tradeoffs and facilitate coexistence (e.g., [53,54]) is an important step to better understand the dynamics of trait and species composition in nature.

Modeling competition as a function of the trait that also determines carrying capacity revealed some differences compared to previous studies of metacommunities, including those that consider phenotypic evolution. In studies of community ecology, the pattern of species sorting (where species traits show a strong match to their local environment; [13,14]) is evaluated by considering the proportion of species composition that is explained by environmental variables (e.g. [55,56]). In metacommunity theory, the degree of species sorting is influenced by spatial scale and species dispersal ability [57,58], and models of metacommunities that consider phenotypic evolution demonstrated that local adaptation alters expected patterns of species sorting. These studies generally indicated that the replacement of locally maladapted species by immigrants from other patches (the ecological process of species sorting) and the evolutionary process of local adaptation trade off in their relative importance for trait and species composition, depending on relative connectivity and evolution rates [12,16,17,18,19]. However, these studies don’t take into account that competition can also be a direct selection pressure that causes local adaptation towards trait values that diverge from the local environmental optimum (e.g., [27,31]).

When competition strength and carrying capacity are selection pressures that can lead to divergent trait values, the observed pattern of species sorting depends on niche width and the consequent strength of competition. When niche width is low (i.e., σ_α_ = 0.68 in our model), species with similar trait values can coexist (e.g., Figure 2 and Figure 3). Local adaptation is inhibited by immigrant competitors (Figure 3), but the release from competition with other species can also promote evolutionary diversification (branching), where mutant populations are selected not only for adaptation to the local environment but also for divergent trait values that experience less intraspecific competition (e.g., Figure 4). As niche width increases (i.e., σ_α_ = 0.85 and 1.5 in our model), local adaptation is observed for more species in the metacommunity, even at increasing dispersal levels (i.e., the species with the lowest trait value in the metacommunity for σ_α_ = 0.85, Figures S14–S16 for σ_α_ = 0.85; all species for σ_α_ = 1.5, Figures S18–S20). These results suggest that the metacommunity pattern of species sorting is dependent not only on dispersal and site connectivity levels [56] and the speed of evolution (e.g., [16,17,18]), but also on the strength of intra- and interspecific competition.

Our study also showed the pattern of global monopolization observed in previous studies of eco-evolutionary dynamics in metacommunities, where one evolving species dominates the landscape under some conditions [16,17,18]. In our model this depended on the speed of environmental change, and thus on the magnitude of the directional environmental selection pressure (Figure S6; as was observed in Johansson [31]; see [49]). Loeuille and Leibold [16] observed global monopolization of the metacommunity by a single species at a combination of high dispersal and high mutation rates (and they considered a fluctuating environment, instead of a directional change). The studies of Urban and De Meester [17] and Vanoverbeke et al. [18] observed global monopolization in more combinations of dispersal and mutation rates, but they explicitly model scenarios with variable initial patch occupancy and thus consider priority effects in more detail. Our model places communities at an initial state of equilibrium, with no unexplored niche space. Our model also explored in more detail how evolutionary dynamics, independent of the presence of diverse species with distinct trait values, lead to differential maintenance of phenotypic diversity (Figure 3, Figure 4 and Figure S3), and our results suggest that future models of eco-evolutionary dynamics can benefit from exploring a more diverse array of models for quantitative trait evolution. Additional factors to consider are the evolution of multiple correlated traits [59], considering distinct genetic variances for species [60], and considering response to multiple environmental stressors [61].

### 4.4. Eco-Evolutionary Feedback Loops and Adaptive Dynamics

Trait evolution in species that compete for resources and inhabit a metacommunity is influenced by intriguing mechanisms that were previously detailed in the fields of adaptive dynamics and eco-evolutionary dynamics. Eco-evolutionary feedback loops are cyclical interactions between ecology and evolution, where changes in ecological interactions drive evolutionary change in traits that, in turn, feed back to alter the ecological interactions in a continuing process [62]. These feedback loops have been demonstrated empirically for population, host-parasite and predator-prey, and ecosystem dynamics in closed systems (e.g., [63,64,65,66]) and for evolution of dispersal and metapopulation dynamics in spatially structured populations [67,68]. There is some evidence that eco-evolutionary feedbacks can facilitate coexistence [69], but these are not well-studied at the metacommunity scale (but see [70] for a model of predator-prey interactions in a spatially structured system). We identified an eco-evolutionary feedback loop in our system when we considered dispersal and directional environmental change simultaneously—evolution in patch 3 selects for individuals with higher trait values, immigrants from patch 3 with high trait values shift the local selection pressure from competition in patch 1 and select for individuals with lower trait values, and then immigrants from patch 1 with lower trait values move to patch 3, where they complete the feedback loop, increasing selection for higher trait values. This effect was observed at all combinations of dispersal and environmental change rate >0 but is easiest to visualize at the highest levels of both (Figure 4b and Figure S13). This is a novel mechanism for an eco-evolutionary feedback that involves dispersal maintaining dynamic reinforcement between competition and phenotypic evolution. Dispersal therefore has an effect not only of causing some resident species to go extinct, but also of altering the local fitness landscape as novel selection pressure from competition is introduced (e.g., Figure 2b). The feedback loop is responsible for maintaining an increased amount of genetic diversity in patch 1 relative to the other patches (observed when comparing *d* = 0 to *d* = 0.01 when σ_α_ = 0.68 and ∆ > 0; for ∆ = 10^−5^; see Figure 3; for ∆ = 4 × 10^−4^, see Figure 4).

Adaptive dynamics theory is well suited to model eco-evolutionary dynamics because the equations explicitly link trait evolution to ecological processes and have been used to understand the process of evolutionary rescue (where initially maladapted populations adapt to the environment and avoid extinction [33,71]). Our simulation model reproduced expected elements observed in previous adaptive dynamics models, such as the branching process that produced evolutionarily stable trait values for species generated in our initialization step. The same branching process produced the divergence of populations into multiple coexisting subpopulations within a site observed in some conditions, such as in patch 3 when the rate of environmental change is slow and sites are linked by dispersal (final 10,000 generations in Figure 3). Adaptive dynamics models have also revealed processes where populations can evolve towards either their own extinction (evolutionary suicide [72,73,74]) or between two equilibria, one at low and one at high population density (evolutionary collapse [75]). In both instances, selection can produce mutants with traits that inhabit a trait space beyond the extinction boundary, where the mutant population cannot persist even though it can be maintained for long periods of time. In some conditions, our model reflected the long-term persistence but eventual extinction of mutants in the population. For example with σ_α_ = 0.68, *d* = 0.1, and ∆ = 4 × 10^−4^ (Figure 4), a primary resident population in patch 3 tracked the local environmental optimum, but immigrants from patch 3 shifted the local competition curve in other patches in a way that permitted mutant individuals with a very wide range of trait values to persist for long periods of time before declining to extinction. Figure 4 shows that populations branch from the main resident population and survive, decline to extinction, and the process repeats itself. Although this process may not be the same as the mechanism that produces suicide and collapse, it indicates that maladaptive diversity can be maintained for relatively long periods of time.

## 5. Conclusions

As species encounter changing climates, it is critical to understand whether they will adapt, move to more suitable habitats, or instead decline to extinction. Models of species range shifts are increasingly considering genetic variation, evolution, and species interactions (e.g. [76]), and species distribution models increasingly include species association matrices as well as local adaptation and phenotypic plasticity [77,78,79]. Accurate representations of these more complex processes will be necessary to forecast how biodiversity will track climate change [52]. Our study considers how trait evolution in metacommunities can respond to environmental change and it adds to previous studies by considering phenotypic evolution in a trait that represents a balance between two selection pressures—the changing environment (which is density independent) and the strength of competition with other populations and species consuming a similar range of resources (which is density dependent). The placement of the evolving community in a landscape introduced feedbacks between eco-evolutionary processes that are driven by dispersal and that maintain biodiversity despite the increased connectivity of species among sites. One possible consequence of habitat fragmentation or loss may be the disruption of these spatial eco-evolutionary feedbacks and a resulting loss of biodiversity.

## Supplementary Materials

The following are available online at https://www.mdpi.com/2073-4425/11/12/1433/s1, Supplementary Information, Figures S1–S29. Supplementary information for eco-evolutionary feedbacks and the maintenance of metacommunity diversity in a changing environment. Figure S1: The evolutionary history of a randomly chosen sample of initial communities, across different initial trait values *x*_0_ (two randomly chosen runs for each combination of σ_α_ and *x*_0_ = −2, 0, and 2 are shown). In the plot, points represent trait values for all populations present at generation *t* (i.e., at least one individual exists with that trait value, population size is not represented), and a horizontal line at the optimal trait value is shown. These are not the initial communities that were used in the simulation (see *Methods,* *Metacommunity initialization* and Figure 1c), but instead represent how evolutionary trajectories can vary in the time of branching and metastable positions of phenotypic clusters. Trajectories are shown for 40,000 generations (note that *y* axis positions differ, to better compare the similarities between repetitions). Pictured are trajectories for σ_α_ = 0.68: *x*_0_ = −2 (a), *x*_0_ = 0 (b), *x*_0_ = 2 (c); σ_α_ = 0.85: *x*_0_ = −2 (d), *x*_0_ = 0 (e), *x*_0_ = 2 (f); and σ_α_ = 1.5: *x*_0_ = −2 (h), *x*_0_ = 0 (i), *x*_0_ = 2 (j). Figure S2: Phenotypic distributions over time when competition strength is low (σ_α_ = 0.68) and there is intermediate dispersal (*d* = 0.01), across a range of rates of environmental change (∆ = 0, 10^−5^, and 4 × 10^−4^ from top to bottom), for 50,000 generations (the time series are thinned to show every 800 generations). Columns represent three patches, and features of the plots are otherwise as described in Figure 2. The nine plots with color (columns 4–6) have the same information as the nine plots without color (columns 1–3), and both are shown so that both intra- and interspecific diversity are visible. Note that the first row (∆ = 0) corresponds to the second column in Figure 2 (but showing 50,000 instead of 5000 generations). Figure S3: Phenotypic distributions over time when competition strength is low (σ_α_ = 0.68) and there is high dispersal (*d* = 0.1), across a range of rates of environmental change (∆ = 0, 10^−5^, and 4 × 10^−4^ from top to bottom), for 50,000 generations (the time series are thinned to show every 800 generations). Columns represent three patches, and features of plots are otherwise as described in Figure 2. The nine plots with color (columns 4–6) have the same information as the nine plots without color (columns 1–3), and both are shown so that both intra- and interspecific diversity are visible. Note that the first row (∆ = 0) corresponds to the third column in Figure 2 (but showing 50,000 instead of 5000 generations). Figure S4: Phenotypic distributions over time when competition strength is low (σ_α_ = 0.68) and there is no dispersal (*d* = 0), across a range of rates of environmental change (∆ = 0, 10^−5^, and 4 × 10^−4^ from top to bottom), for 15,000 generations (the time series are thinned to show every 250 generations). This figure contains the same results as Figure 3a–c, but the limits of the *x*-axes are the same across all the levels of environmental change, to better visualize the intraspecific diversity across conditions. Columns represent three patches. Features of plots are as described in Figure 2. Figure S5: Inter- and intraspecific diversity, deviation from optimum trait value, and coefficient of variation over time in an unchanging environment (∆ = 0, σ_α_ = 0.68, *d* = 0, 0.01, and 0.1). Row 1 is interspecific diversity (grey: α, black: γ, orange: β). Row 2 is intraspecific diversity, where every color is a species (corresponding to the colors in Figure 2). Row 3 is the sum of squared deviations of trait values from the local environmental optimum trait value for all individuals in a patch (black: patch 1, red: patch 2, blue: patch 3). Row 4 is the coefficient of variation of total population size across all species at the local (patch 1: grey, patch 2: light blue, patch 3: orange) and metacommunity (black) level. Figure S6: Competition, carrying capacity, and population trait values in a changing environment. In all plots, σ_α_ = 0.68 and *d* = 0, in (a) ∆ = 10^−5^, and in (b) ∆ = 4 × 10^−4^. Competition functions (*C*(*x*), red), carrying capacity functions (*K*(*x*), black), population (black points), and all other features are as given in Figure 2. Figure S7: Inter- and intraspecific diversity, deviation from optimum trait value, and coefficient of variation over time in a slowly changing environment (∆ = 10^−5^, σ_α_ = 0.68) across a range of dispersal levels (from left to right, *d* = 0, 0.01, and 0.1). Features of all plots are as described in Figure S5. Figure S8: Interspecific diversity over time when competition strength is low (σ_α_ = 0.68) across a range of dispersal levels and rates of environmental change (grey: α diversity, black: γ diversity, orange: β diversity). Rows are increasing levels of environmental change (∆ = 0, 10^−5^, and 4 × 10^−4^ from top to bottom) and columns are increasing levels of dispersal (*d* = 0, 0.01, and 0.1 from left to right). Figure S9: Intraspecific diversity over time when competition strength is low (σ_α_ = 0.68) across a range of dispersal levels and rates of environmental change. Each color represents genetic diversity of a different species. Rows are increasing levels of environmental change (∆ = 0, 10^−5^, and 4 × 10^−4^ from top to bottom) and columns are increasing levels of dispersal (*d* = 0, 0.01, and 0.1 from left to right). Figure S10: Sum of squared deviations of trait values from local environmental optimum trait value for all individuals in patches over time when competition strength is low (σ_α_ = 0.68), across a range of dispersal levels and rates of environmental change (black: patch 1, red: patch 2, blue: patch 3). Rows are increasing levels of environmental change (∆ = 0, 10^−5^, and 4 × 10^−4^ from top to bottom) and columns are increasing levels of dispersal (*d* = 0, 0.01, and 0.1 from left to right). Figure S11: Coefficient of variation of total population size across all species at the local (patch 1: grey, patch 2: light blue, patch 3: orange) and metacommunity (black) level over time when competition strength is low (σ_α_ = 0.68), across a range of dispersal levels and rates of environmental change. Rows are increasing levels of environmental change (∆ = 0, 10^−5^, and 4 × 10^−4^ from top to bottom) and columns are increasing levels of dispersal (*d* = 0, 0.01, and 0.1 from left to right). Figure S12: Competition, carrying capacity, and population trait values in a slowly changing environment. In all plots, σ_α_ = 0.68 and ∆ = 10^−5^. Competition functions (*C*(*x*), red), carrying capacity functions (*K*(*x*), black), population (black points), and all other features are as given in Figure 2 and vary across levels of dispersal (*d* = 0, 0.01, 0.1).Figure S13: The impacts of selection by competition and carrying capacity as a function of trait value within a given generation. Results are shown for σ_α_ = 0.68, *d* = 0.1, and ∆ = 4 × 10^−4^, for *t* = 1, 2, 1000, 5000, 10,000, 25,000, and 50,000. Plots (a–g) show the full distribution, and plots (h–n) are the same distributions with the *y* axis truncated to N = 7000−10,000. Within each set of conditions, plots show patch 1–3 from left to right, respectively. The selection pressure from competition is depicted as *C*(*x*) (Equation (3)) and the selection pressure from carrying capacity is shown as *K*(*x*) (Equation (1)). Figure S14: Phenotypic distributions over time when competition strength is intermediate (σ_α_ = 0.85) and there is no dispersal (*d* = 0), across a range of rates of environmental change (∆ = 0, 10^−5^, and 4 × 10^−4^ from top to bottom), for 50,000 generations (the time series are thinned to show every 800 generations). Columns represent three patches, and features of plots are as described in Figure 2. The nine plots with color (columns 4–6) have the same information as the nine plots without color (columns 1–3), and both are shown so that both intra- and interspecific diversity are visible. Figure S15: Phenotypic distributions over time when competition strength is intermediate (σ_α_ = 0.85) and there is intermediate dispersal (*d* = 0.01), across a range of rates of environmental change (∆ = 0, 10^−5^, and 4 × 10^−4^ from top to bottom), for 50,000 generations (the time series are thinned to show every 800 generations). Columns represent three patches, and features of plots are as described in Figure 2. The nine plots with color (columns 4–6) have the same information as the nine plots without color (columns 1–3), and both are shown so that both intra- and interspecific diversity are visible. Figure S16: Phenotypic distributions over time when competition strength is intermediate (σ_α_ = 0.85) and there is high dispersal (*d* = 0.1), across a range of rates of environmental change (∆ = 0, 10^−5^, and 4 × 10^−4^ from top to bottom), for 50,000 generations (the time series are thinned to show every 800 generations). Columns represent three patches, and features of plots are as described in Figure 2. The nine plots with color (columns 4–6) have the same information as the nine plots without color (columns 1–3), and both are shown so that both intra- and interspecific diversity are visible. Figure S17: Interspecific diversity over time when competition strength is intermediate (σ_α_ = 0.85) across a range of dispersal levels and rates of environmental change (grey: α diversity, black: γ diversity, orange: β diversity). Rows are increasing levels of environmental change (∆ = 0, 10^−5^, and 4 × 10^−4^ from top to bottom) and columns are increasing levels of dispersal (*d* = 0, 0.01, and 0.1 from left to right).Figure S18: Phenotypic distributions over time when competition strength is high (σ_α_ = 1.5) and there is no dispersal (*d* = 0), across a range of rates of environmental change (∆ = 0, 10^−5^, and 4 × 10^−4^ from top to bottom), for 50,000 generations (the time series are thinned to show every 800 generations). Columns represent three patches, and features of plots are as described in Figure 2. The nine plots with color (columns 4–6) have the same information as the nine plots without color (columns 1–3), and both are shown so that both intra- and interspecific diversity are visible. Figure S19: Phenotypic distributions over time when competition strength is high (σ_α_ = 1.5) and there is intermediate dispersal (*d* = 0.01), across a range of rates of environmental change (∆ = 0, 10^−5^, and 4 × 10^−4^ from top to bottom), for 50,000 generations (the time series are thinned to show every 800 generations). Columns represent three patches, and features of plots are as described in Figure 2. The nine plots with color (columns 4–6) have the same information as the nine plots without color (columns 1–3), and both are shown so that both intra- and interspecific diversity are visible. Figure S20: Phenotypic distributions over time when competition strength is high (σ_α_ = 1.5) and there is high dispersal (*d* = 0.1), across a range of rates of environmental change (∆ = 0, 10^−5^, and 4 × 10^−4^ from top to bottom), for 50,000 generations (the time series are thinned to show every 800 generations). Columns represent three patches, and features of plots are as described in Figure 2. The nine plots with color (columns 4, 5, and 6) have the same information as the nine plots without color (columns 1–3), and both are shown so that both intra- and interspecific diversity are visible. Figure S21: Interspecific diversity over time when competition strength is high (σ_α_ = 1.5) across a range of dispersal levels and rates of environmental change (grey: α diversity, black: γ diversity, orange: β diversity). Rows are increasing levels of environmental change (∆ = 0, 10^−5^, and 4 × 10^−4^ from top to bottom) and columns are increasing levels of dispersal (*d* = 0, 0.01, and 0.1 from left to right). Figure S22: Competition, carrying capacity, and population trait values in an unchanging environment. In all plots, σ_α_ = 0.85 and ∆ = 0. Competition functions (*C*(*x*), red), carrying capacity functions (*K*(*x*), black), population (black points), and all other features are as given in Figure 2 and vary across levels of dispersal (d = 0, 0.01, 0.1). Figure S23: Intraspecific diversity over time when competition strength is intermediate (σ_α_ = 0.85) across a range of dispersal levels and rates of environmental change. Each color represents genetic diversity of a different species. Rows are increasing levels of environmental change (∆ = 0, 10^−5^, and 4 × 10^−4^ from top to bottom) and columns are increasing levels of dispersal (*d* = 0, 0.01, and 0.1 from left to right).Figure S24: Intraspecific diversity over time when competition strength is high (σ_α_ = 1.5) across a range of dispersal levels and rates of environmental change. Each color represents genetic diversity of a different species. Rows are increasing levels of environmental change (∆ = 0, 10^−5^, and 4 × 10^−4^ from top to bottom) and columns are increasing levels of dispersal (*d* = 0, 0.01, and 0.1 from left to right).Figure S25: Competition, carrying capacity, and population trait values in a slowly changing environment. In all plots, σ_α_ = 0.85 and ∆ = 10^−5^. Competition functions (*C*(*x*), red), carrying capacity functions (*K*(*x*), black), population (black points), and all other features are as given in Figure 2 and vary across levels of dispersal (d = 0, 0.01, 0.1). Figure S26: Sum of squared deviations of trait values from local environmental optimum trait value for all individuals in a patch over time when competition strength is intermediate (σ_α_ = 0.85), across a range of dispersal levels and rates of environmental change (black: patch 1, red: patch 2, blue: patch 3). Rows are increasing levels of environmental change (∆ = 0, 10^−5^, and 4 × 10^−4^ from top to bottom) and columns are increasing levels of dispersal (*d* = 0, 0.01, and 0.1 from left to right). Figure S27: Coefficient of variation of total population size across all species at the local (patch 1: grey, patch 2: light blue, patch 3: orange) and metacommunity (black) level over time when competition strength is intermediate (σ_α_ = 0.85), across a range of dispersal levels and rates of environmental change. Rows are increasing levels of environmental change (∆ = 0, 10^−5^, and 4 × 10^−4^ from top to bottom) and columns are increasing levels of dispersal (*d* = 0, 0.01, and 0.1 from left to right). Figure S28: Sum of squared deviations of trait values from local environmental optimum trait value for all individuals in a patch over time when competition strength is high (σ_α_ = 1.5), across a range of dispersal levels and rates of environmental change (black: patch 1, red: patch 2, blue: patch 3). Rows are increasing levels of environmental change (∆ = 0, 10^−5^, and 4 × 10^−4^ from top to bottom) and columns are increasing levels of dispersal (*d* = 0, 0.01, and 0.1 from left to right). Figure S29: Coefficient of variation of total population size across all species at the local (patch 1: grey, patch 2: light blue, patch 3: orange) and metacommunity (black) level over time when competition strength is high (σ_α_ = 1.5), across a range of dispersal levels and rates of environmental change. Rows are increasing levels of environmental change (∆ = 0, 10^−5^, and 4 × 10^−4^ from top to bottom) and columns are increasing levels of dispersal (*d* = 0, 0.01, and 0.1 from left to right). Supplementary Code: Code for simulation and results of 27 simulation conditions available on the GitHub page of J.H. Pantel (https://github.com/jhpantel/EcoEvoMC).
