# Supplementary material for: Eco-Evolutionary Feedbacks and the Maintenance of Metacommunity Diversity in a Changing Environment
Source: Genes (Basel). 2020 Nov 28;11(12):1433. doi: 10.3390/genes11121433 (PMC7761218; doi:10.3390/genes11121433)
Supplement: Supplementary file 1 [file genes-11-01433-s001.zip › Figure_S1.pdf]

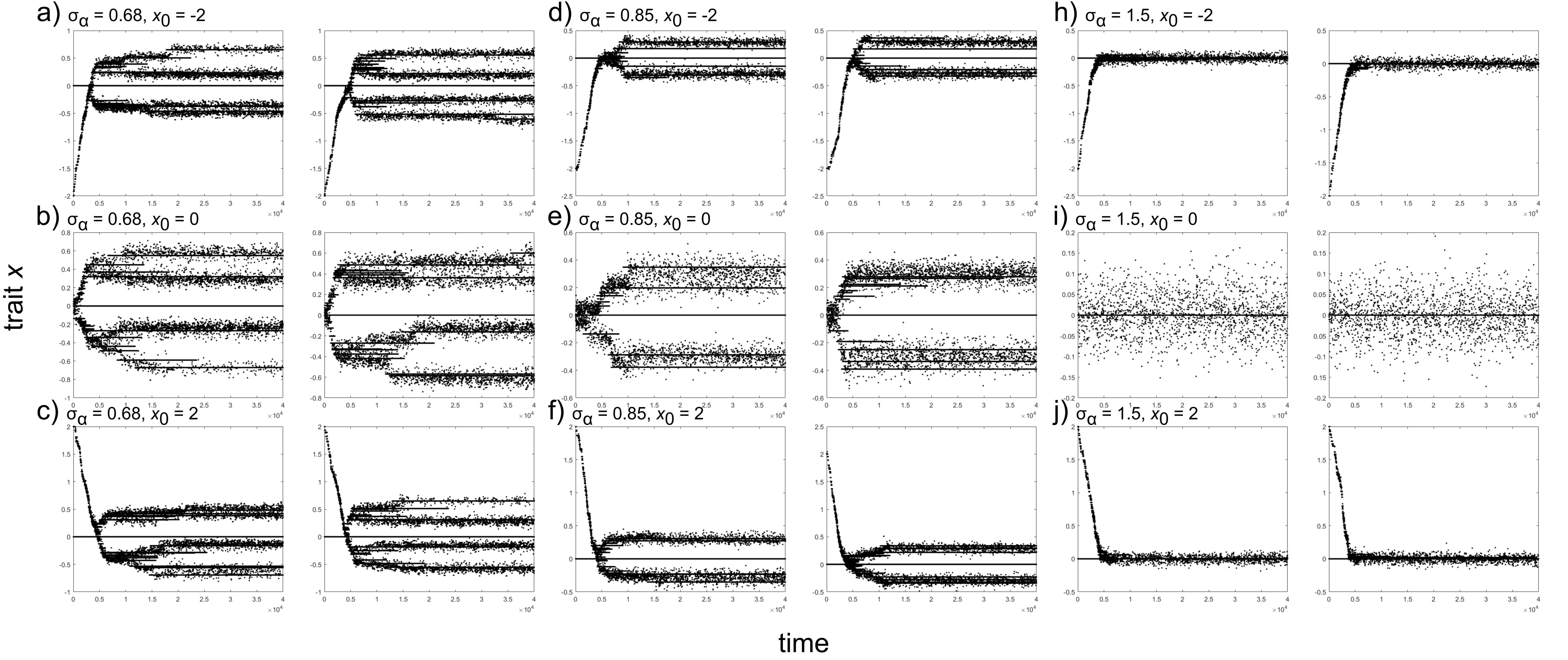

a) σ_α = 0.68, x_0 = -2
b) σ_α = 0.68, x_0 = 0
c) σ_α = 0.68, x_0 = 2
d) σ_α = 0.85, x_0 = -2
e) σ_α = 0.85, x_0 = 0
f) σ_α = 0.85, x_0 = 2
h) σ_α = 1.5, x_0 = -2
i) σ_α = 1.5, x_0 = 0
j) σ_α = 1.5, x_0 = 2
trait x
time
