# Supplementary figures and images for: Eco-Evolutionary Feedbacks and the Maintenance of Metacommunity Diversity in a Changing Environment
Source: Genes (Basel). 2020 Nov 28;11(12):1433. doi: 10.3390/genes11121433 (PMC7761218; doi:10.3390/genes11121433)

$\Delta=0$   
 $\sigma_\alpha=0.68, d=0$

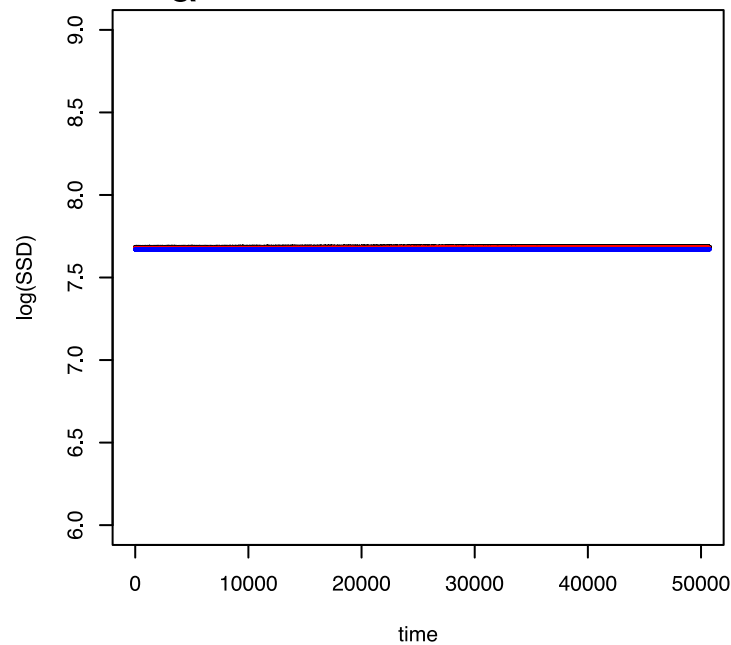

$d=0.01$

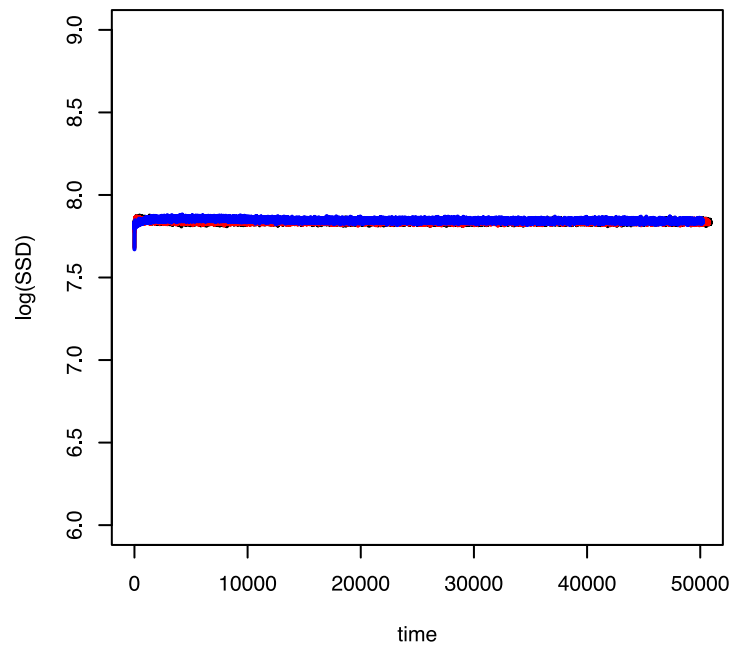

$d=0.1$

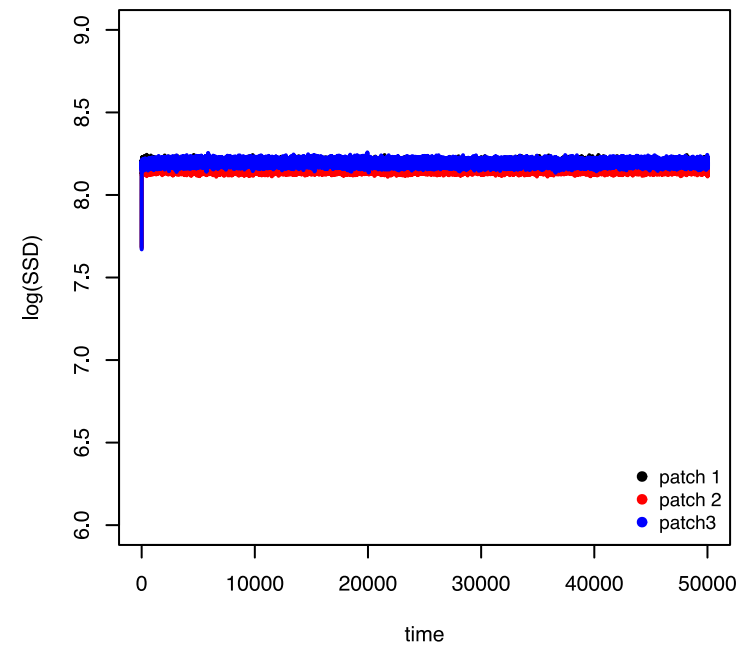

$\Delta=10^{-5}$

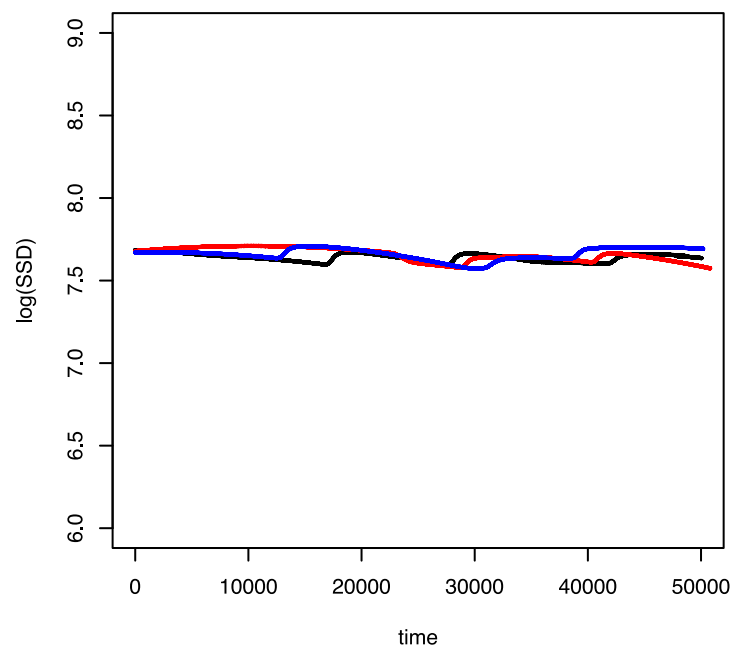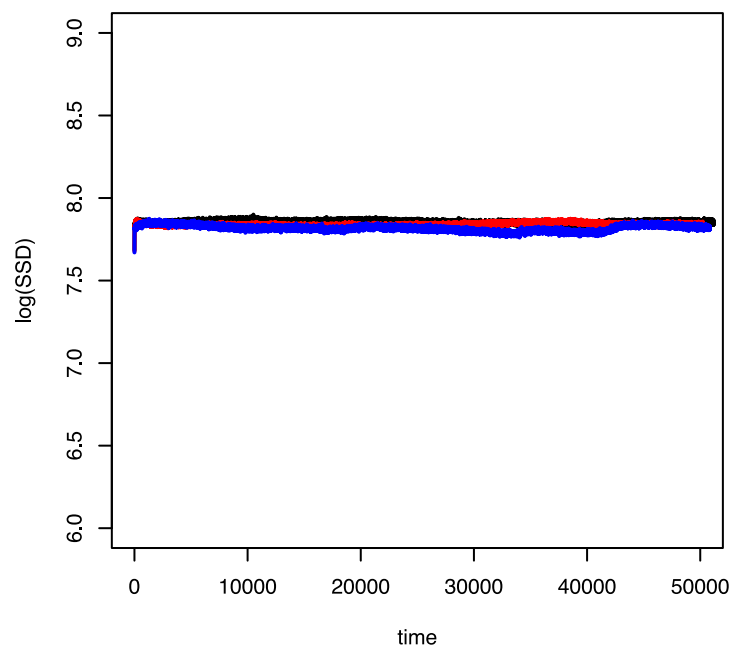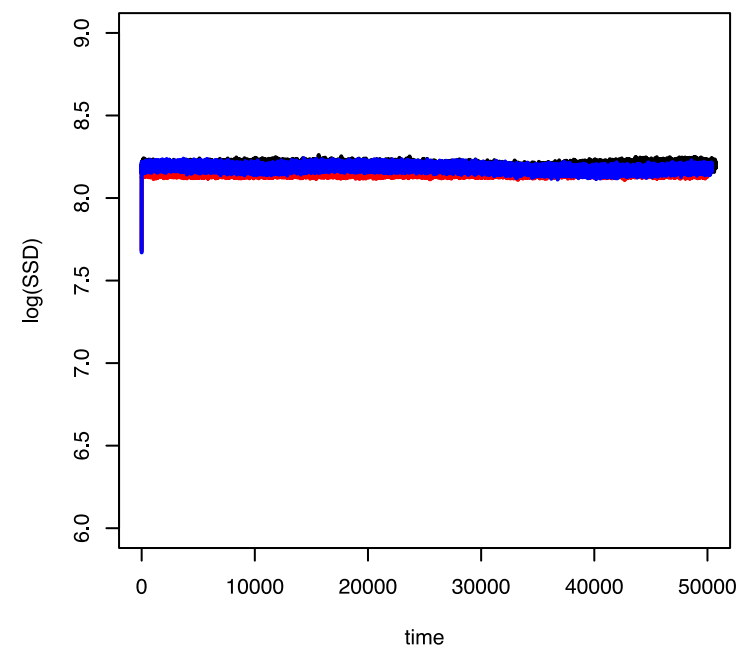

$\Delta=4.4 \times 10^{-4}$

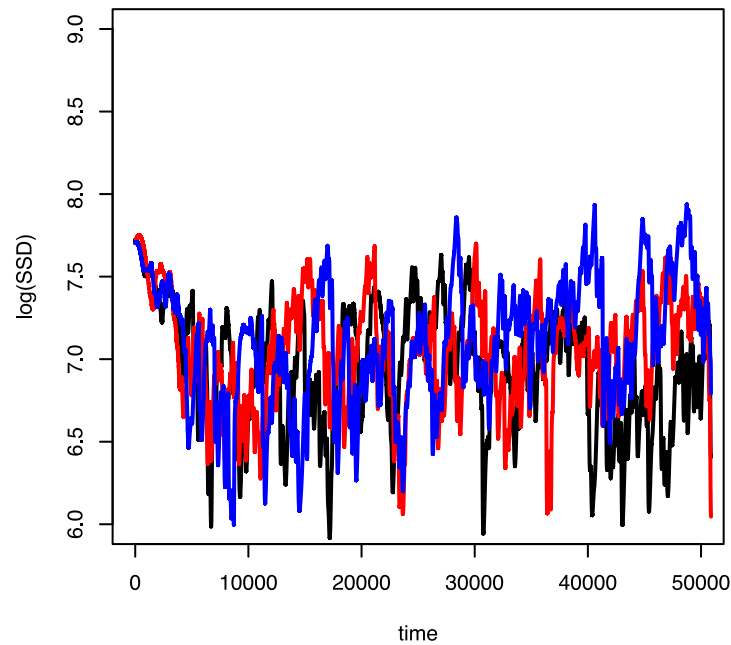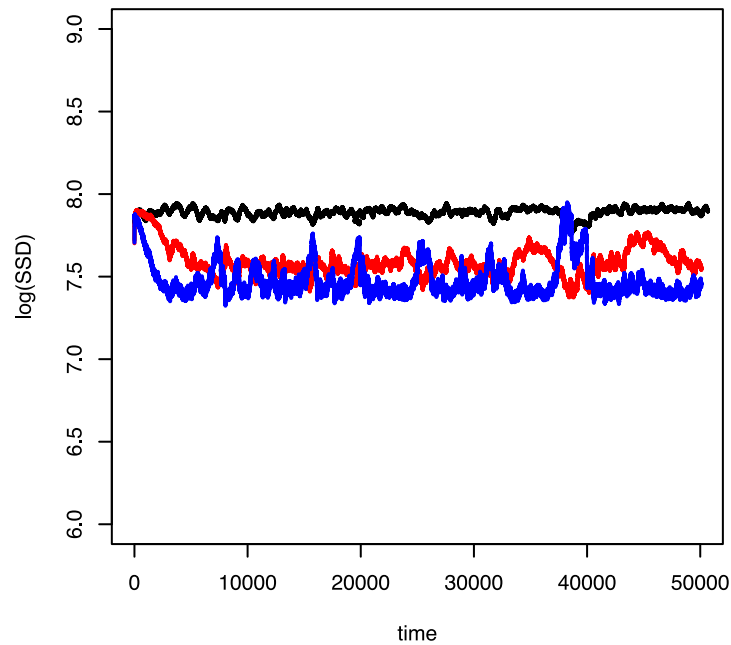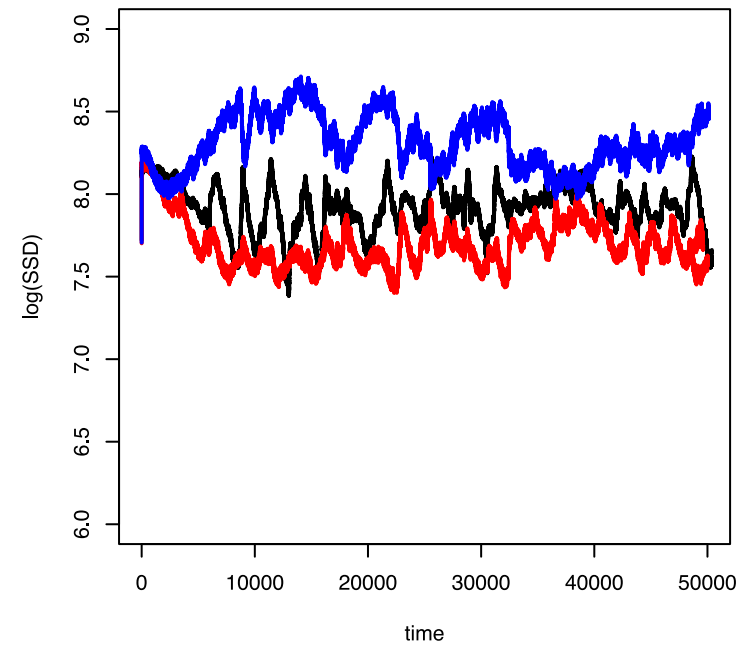

Supplement: Supplementary file 1 [file genes-11-01433-s001.zip › Figure_S10.pdf]

$\Delta=0$   
 $\sigma_\alpha=0.68, d=0$

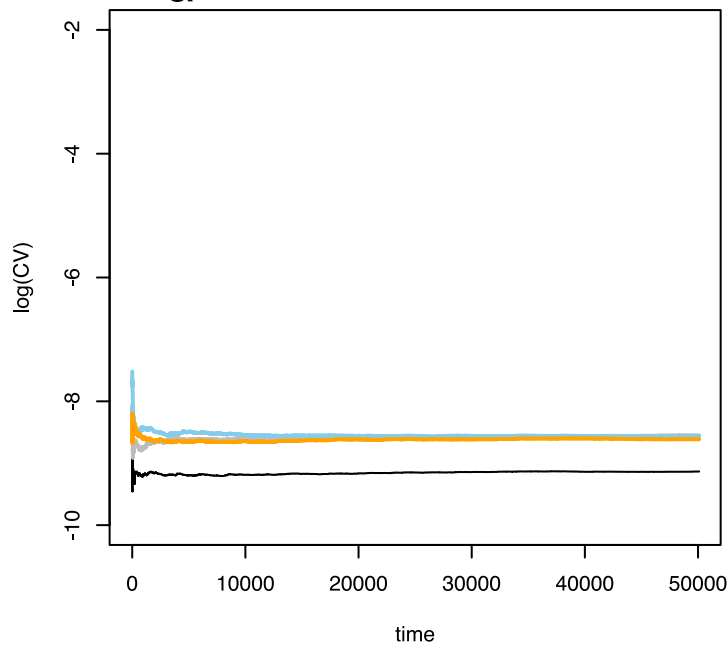

$d=0.01$

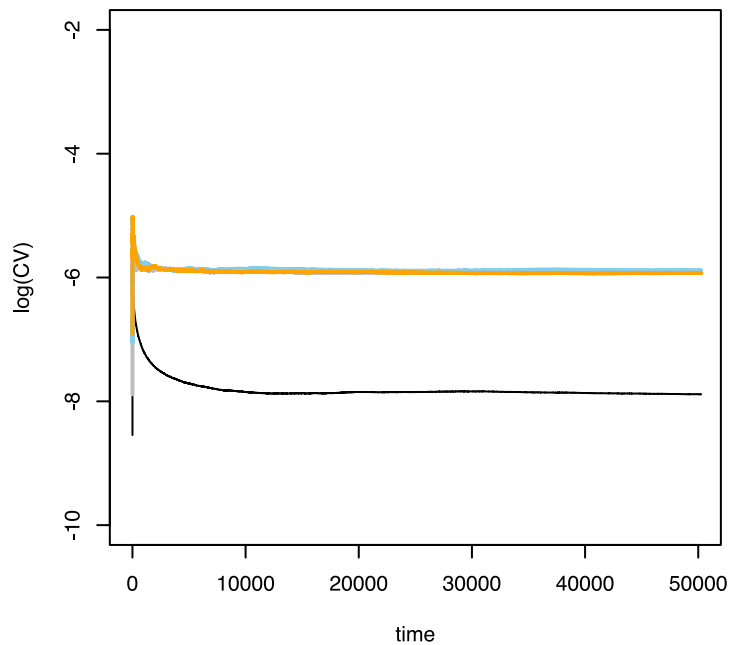

$d=0.1$

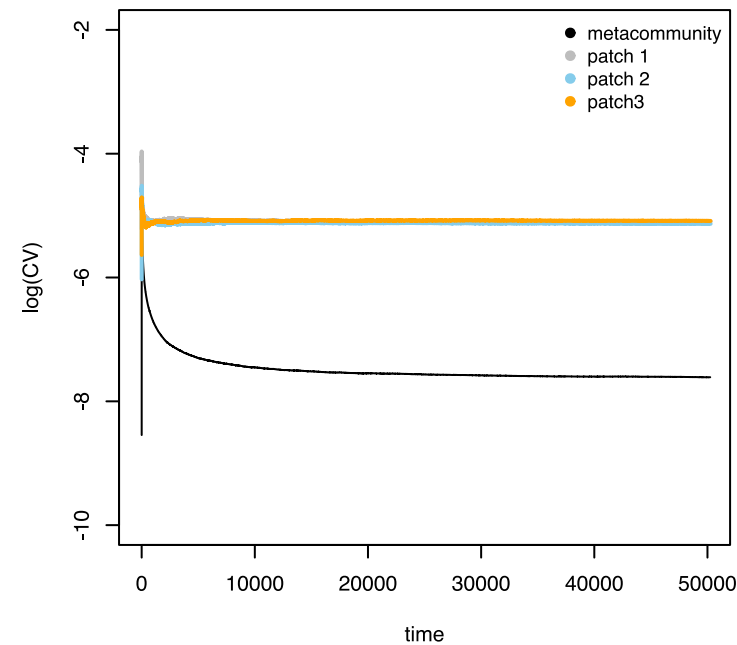

$\Delta=10^{-5}$

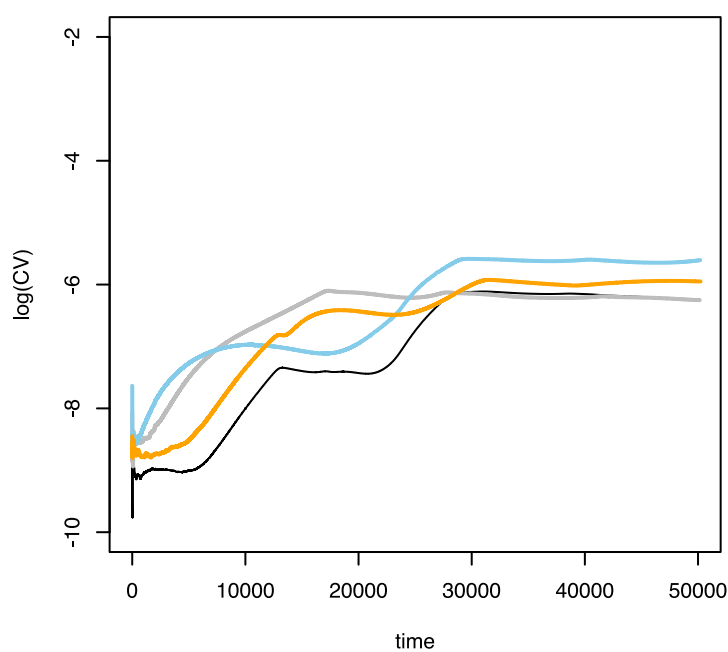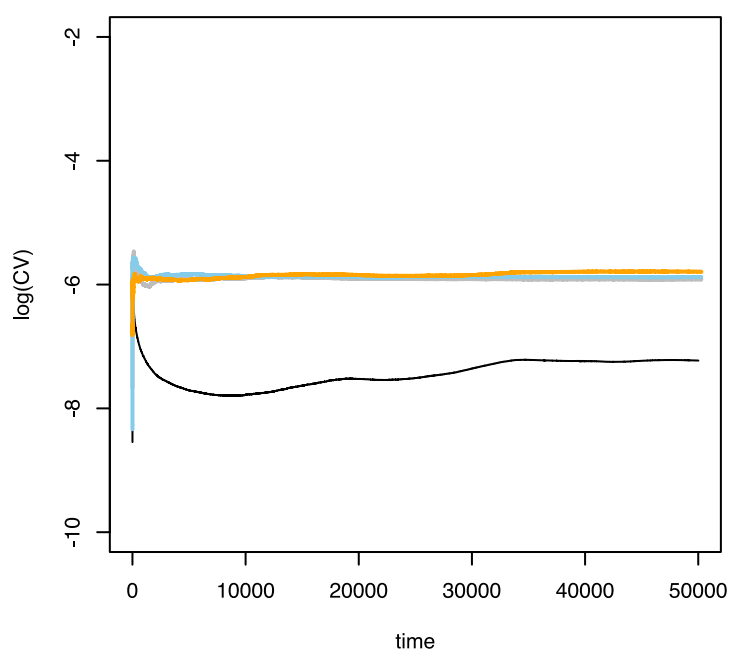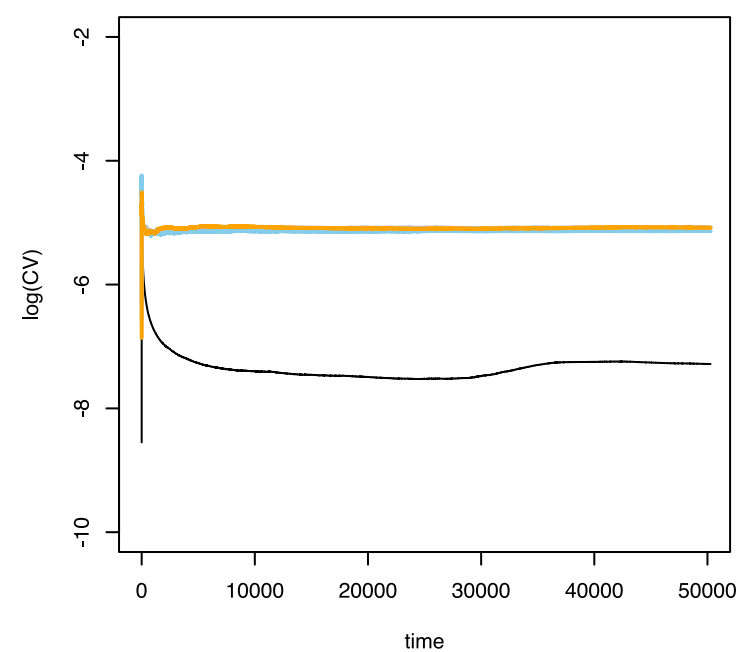

$\Delta=4.4 \times 10^{-4}$

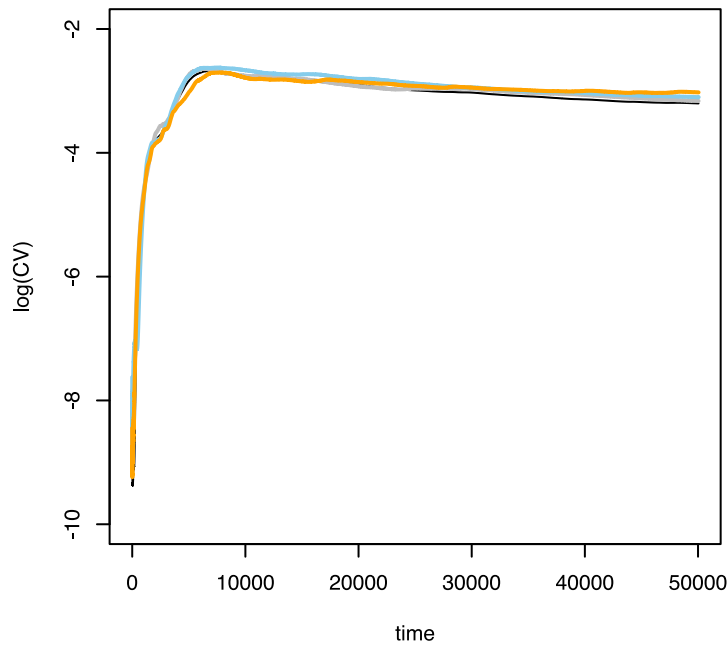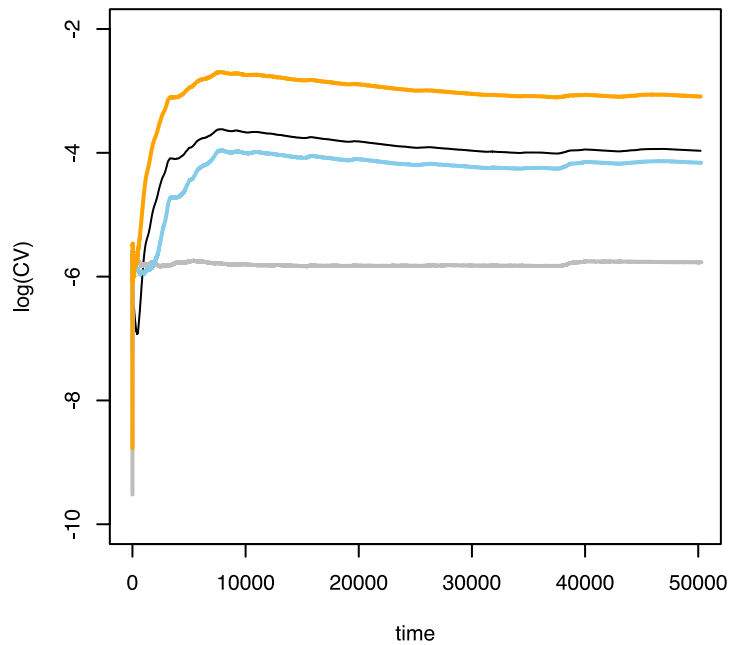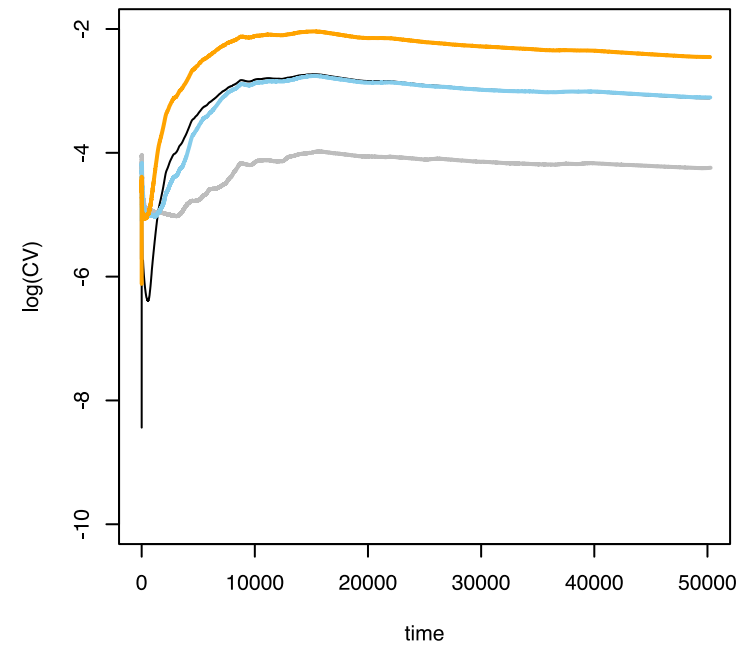

Supplement: Supplementary file 1 [file genes-11-01433-s001.zip › Figure_S11.pdf]

$d = 0$

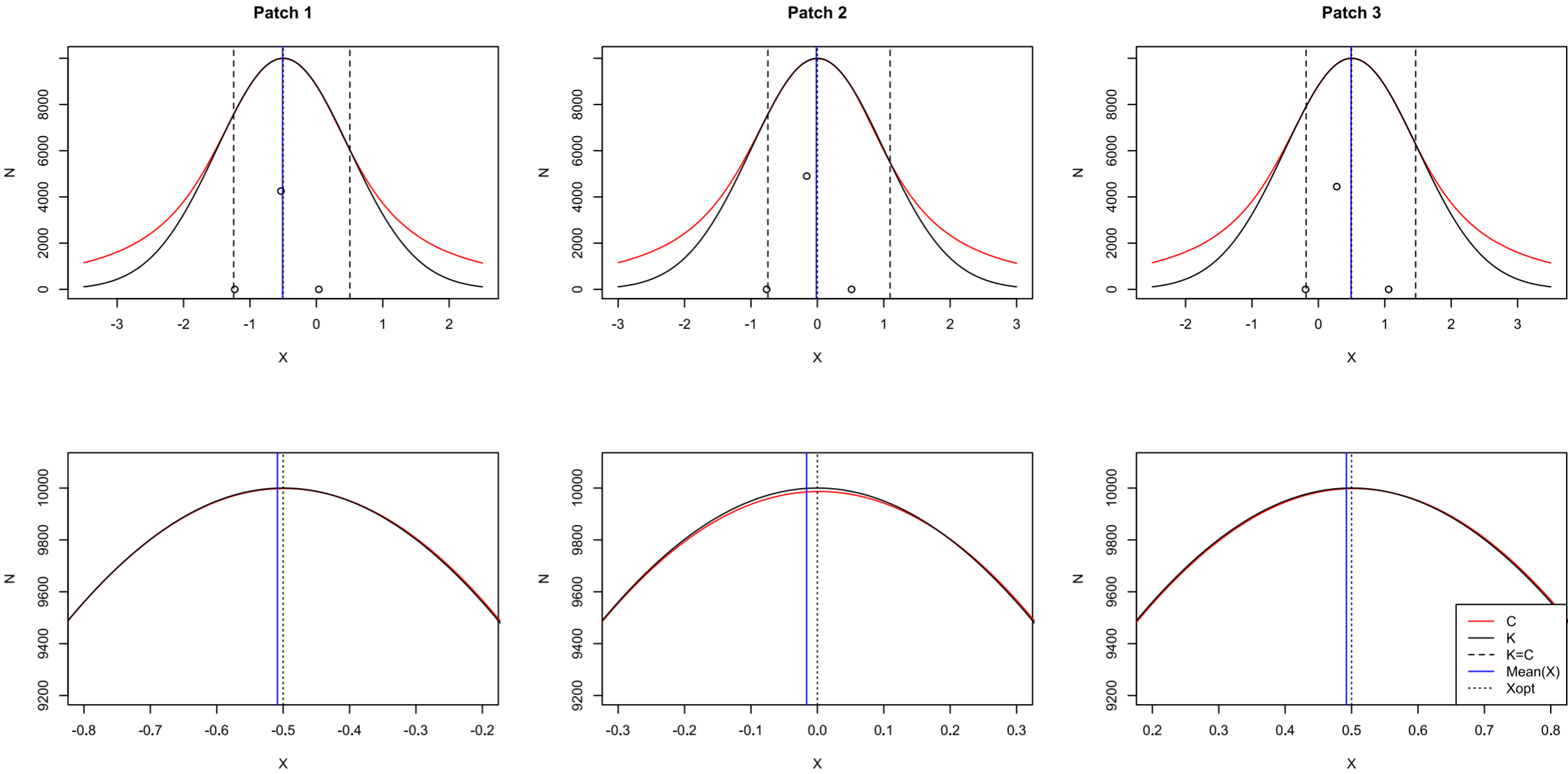

$d = 0.01$

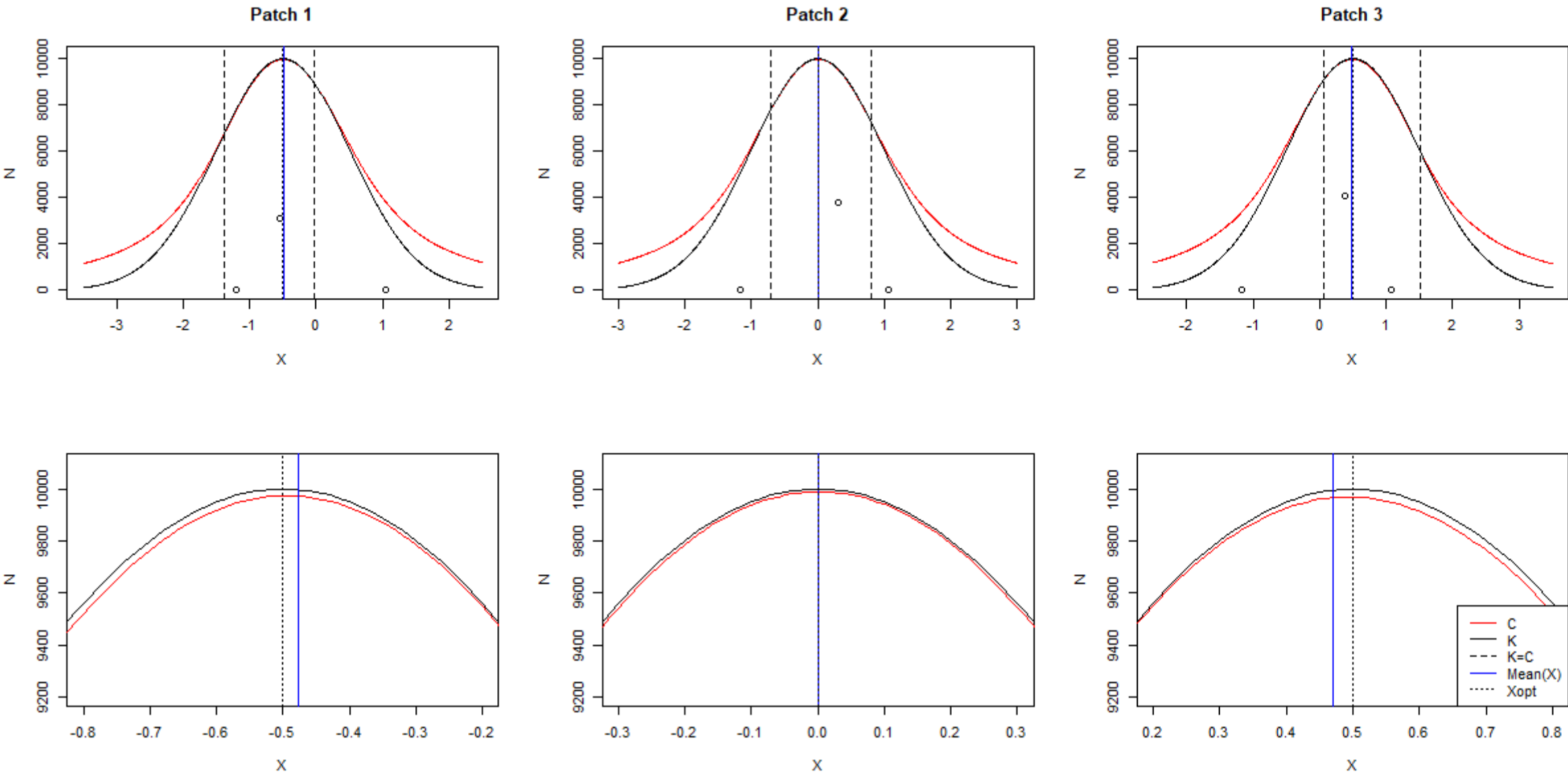

$d = 0.1$

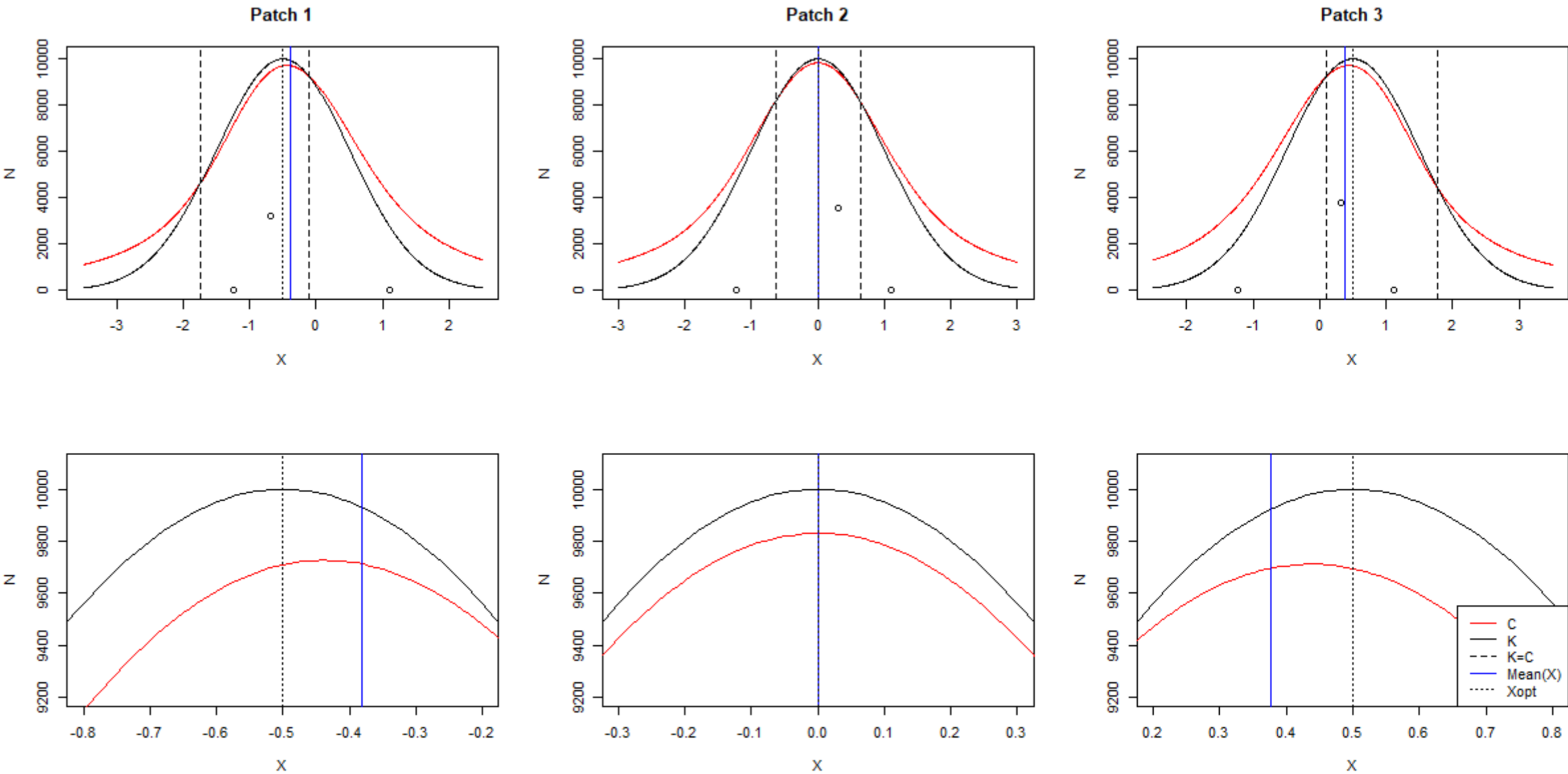

Supplement: Supplementary file 1 [file genes-11-01433-s001.zip › Figure_S12.pdf]

$$\sigma_\alpha = 0.68, d = 0.1, \Delta = 10 \times 10^{-4}$$

N

a)  $t = 1$

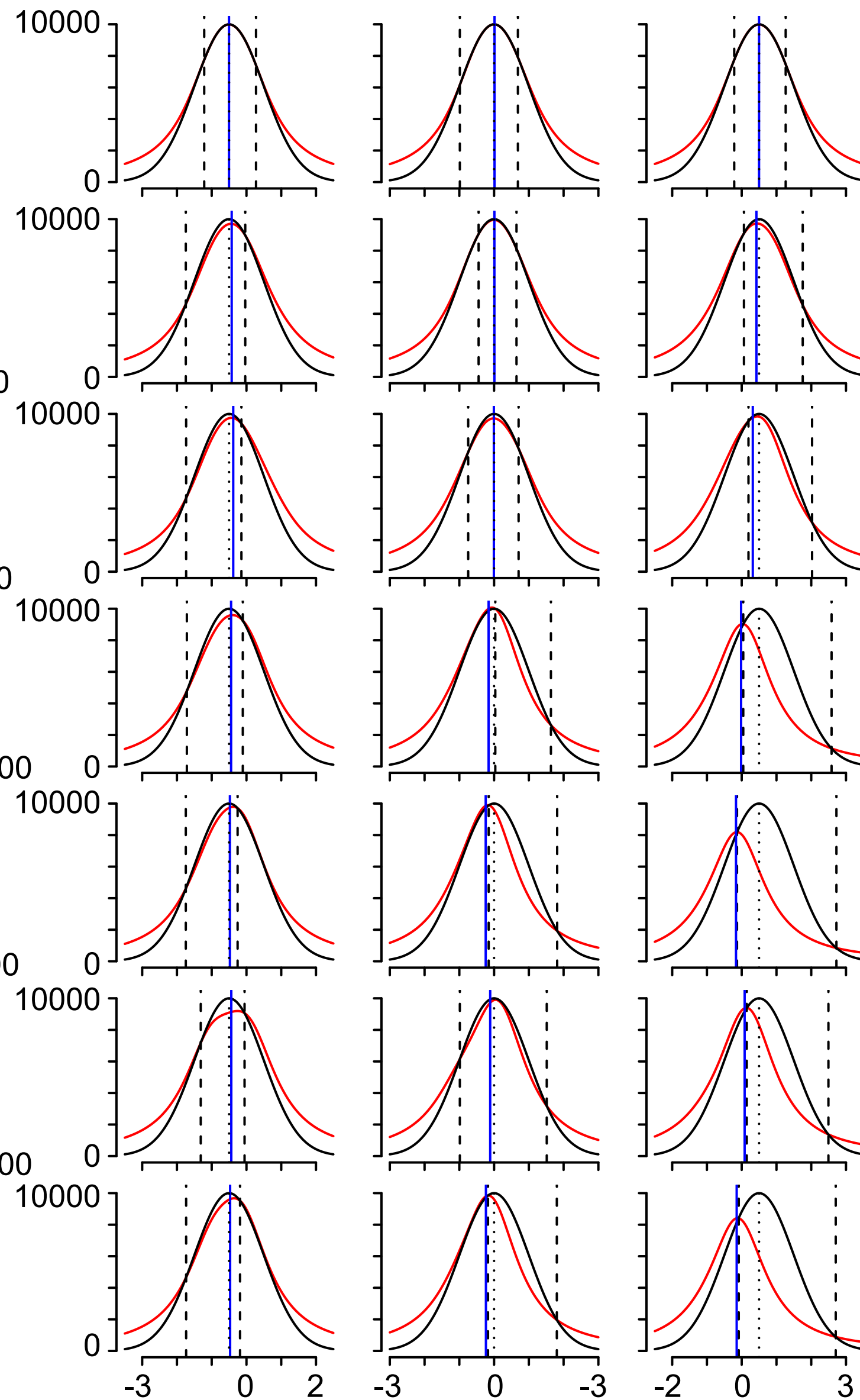

b)  $t = 2$

c)  $t = 1000$

d)  $t = 5000$

e)  $t = 10000$

f)  $t = 25000$

g)  $t = 50000$

h)

i)

j)

k)

l)

m)

n)

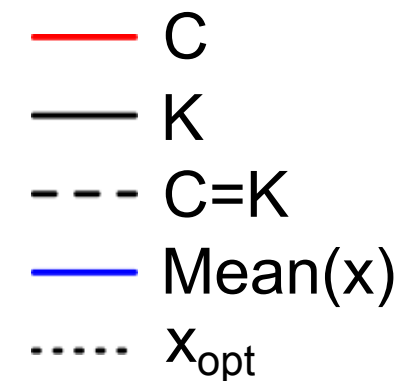

trait x

Supplement: Supplementary file 1 [file genes-11-01433-s001.zip › Figure_S13.pdf]

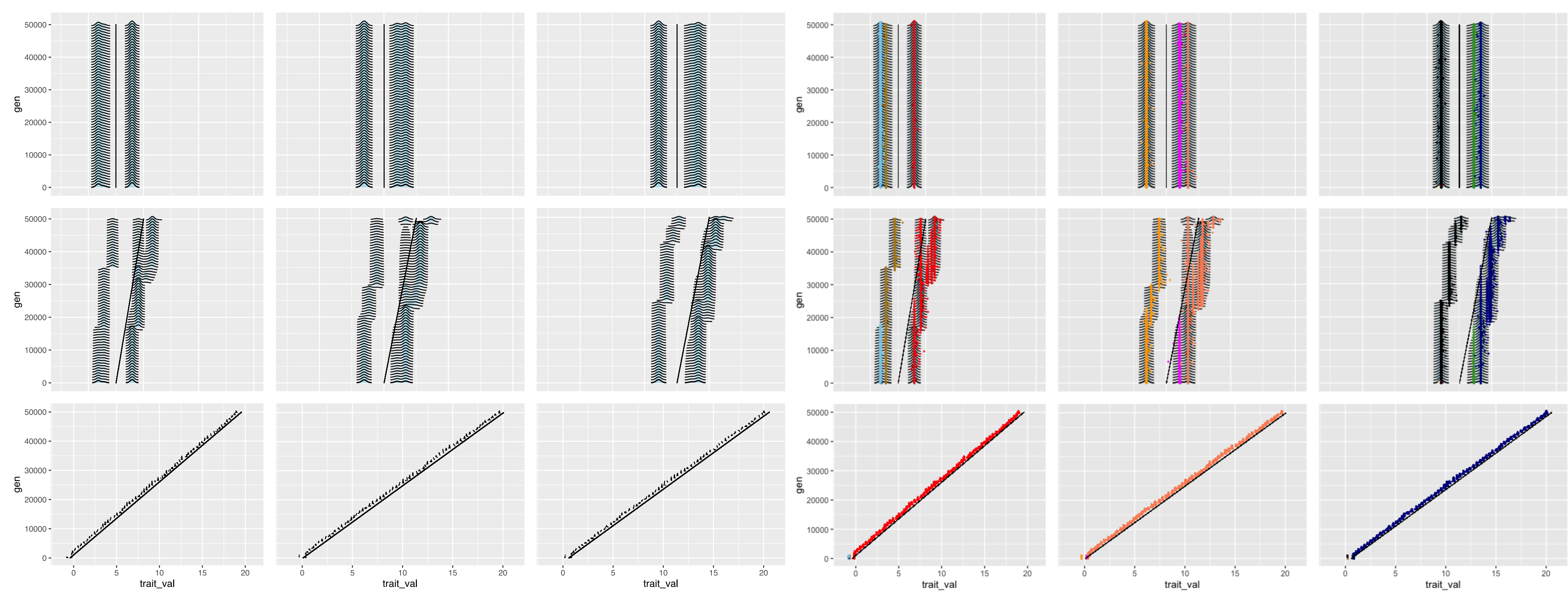

Supplement: Supplementary file 1 [file genes-11-01433-s001.zip › Figure_S14.pdf]

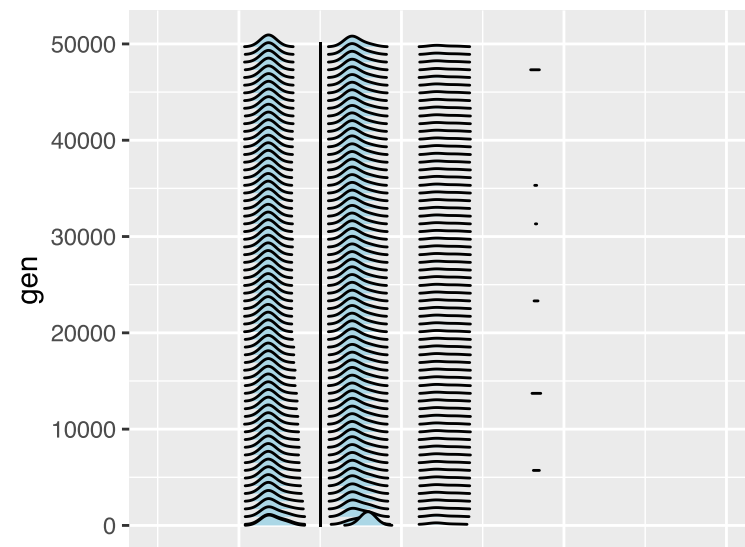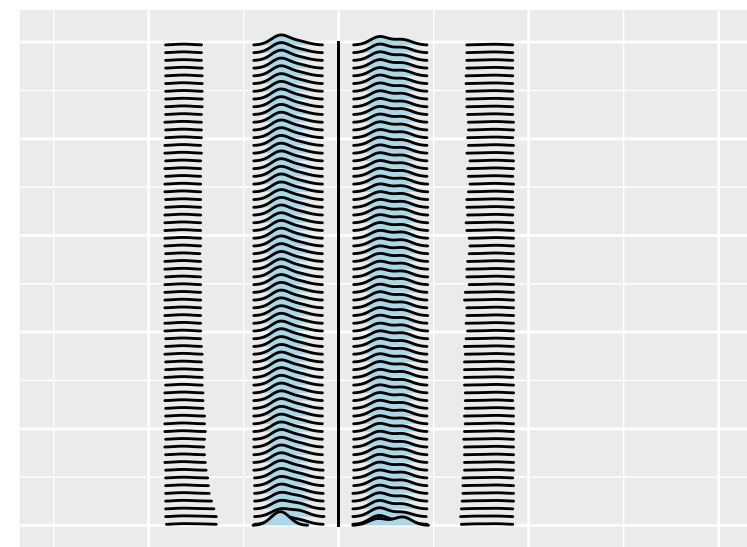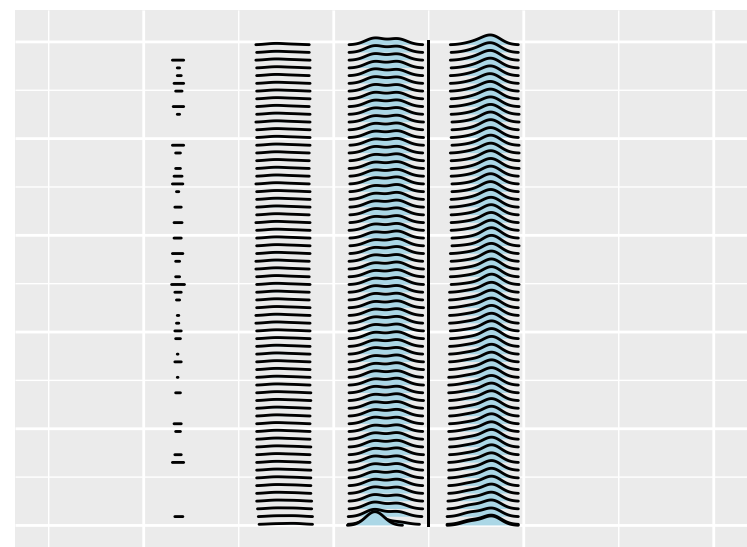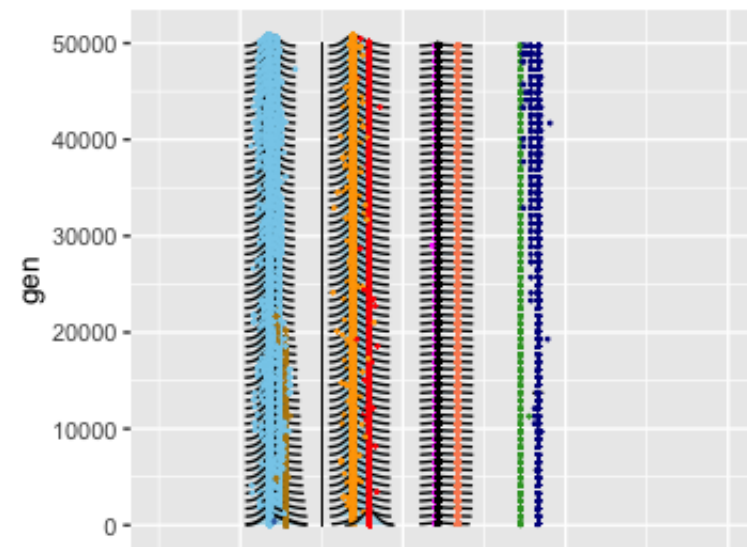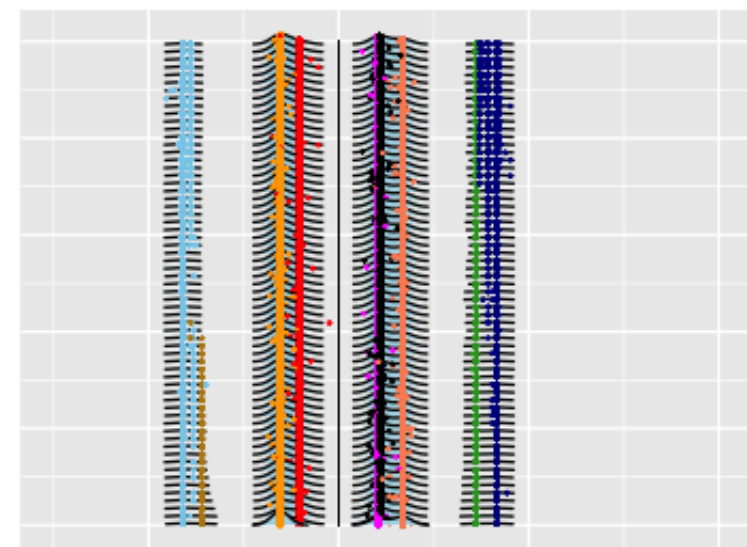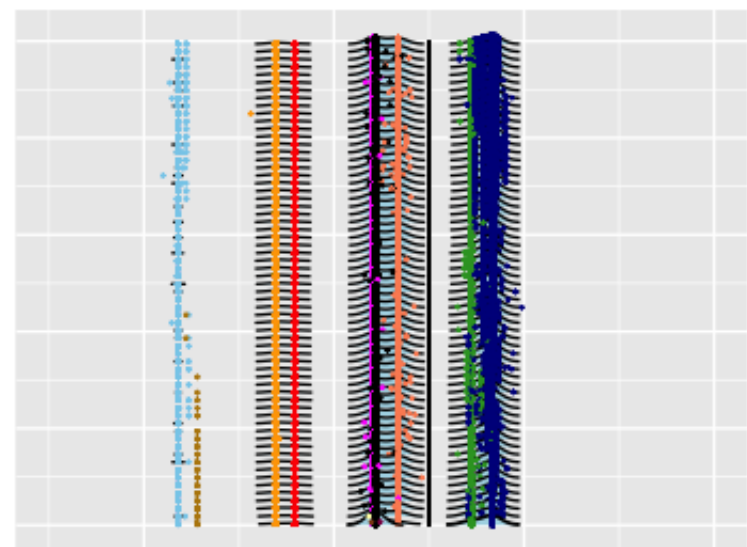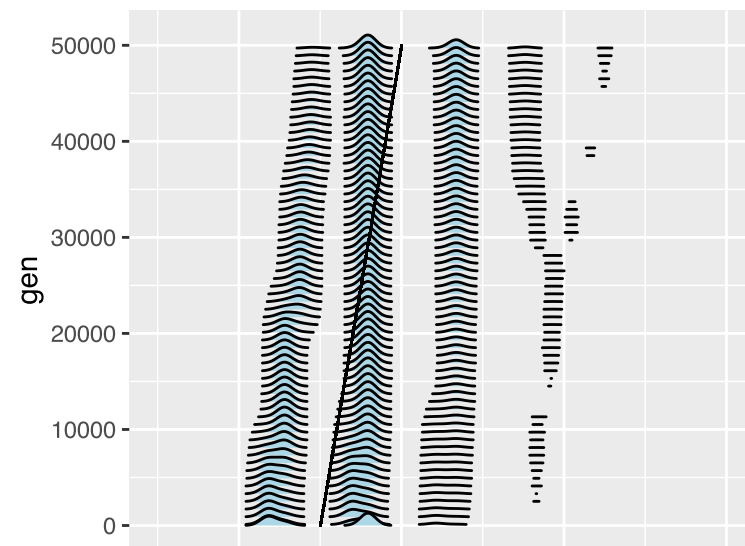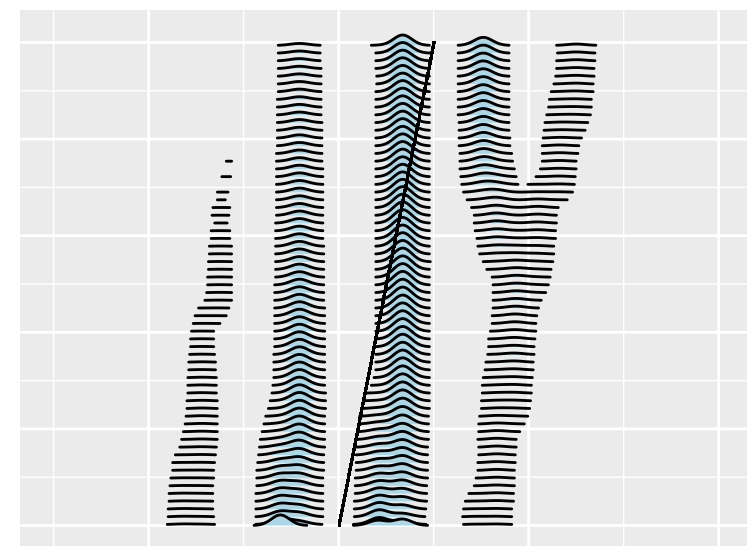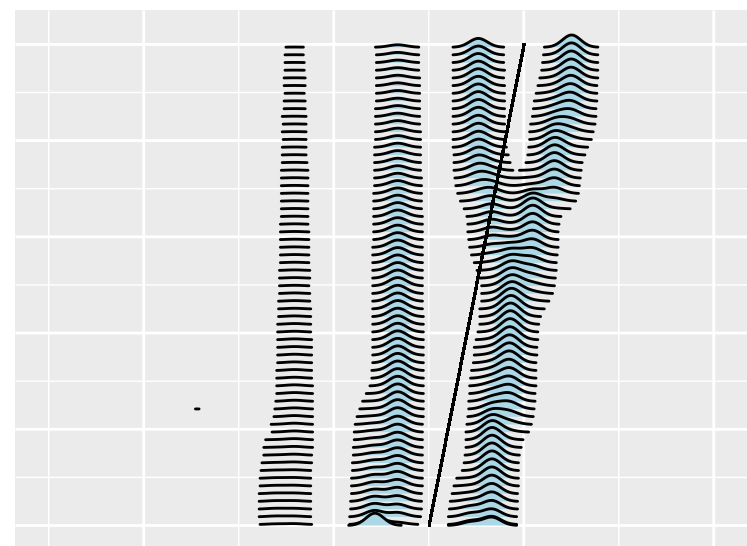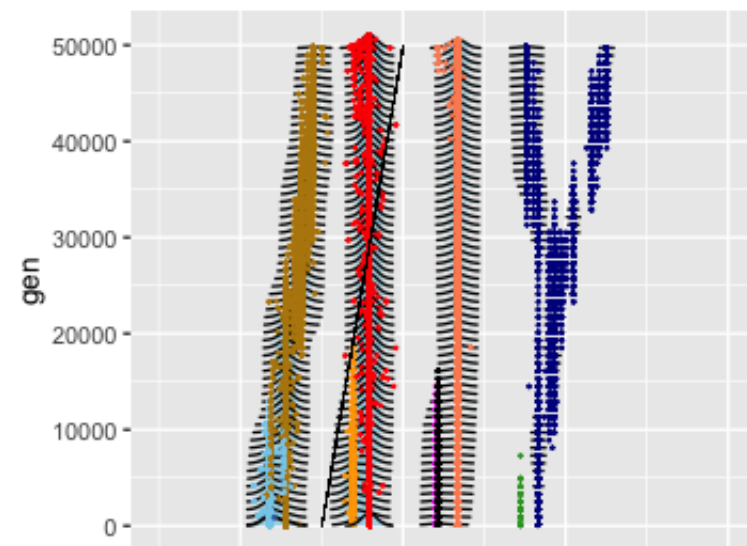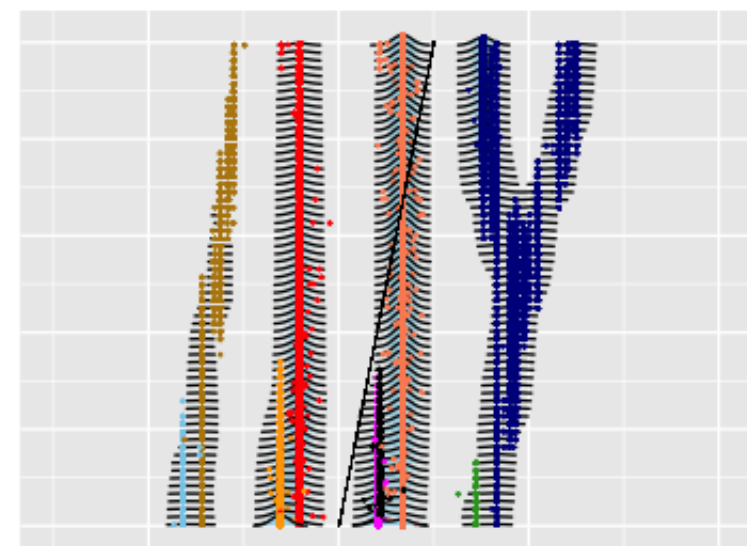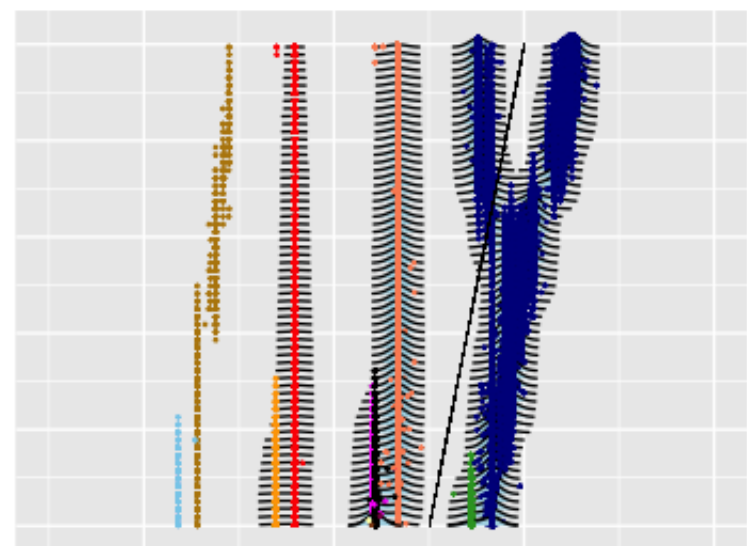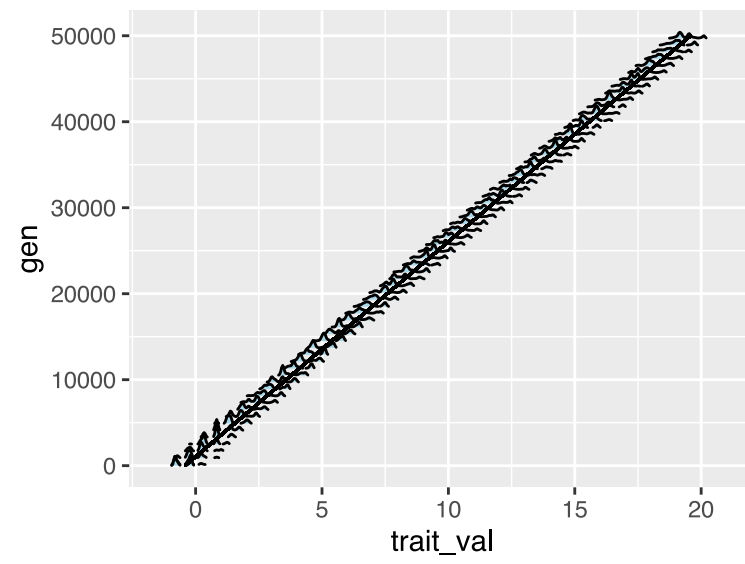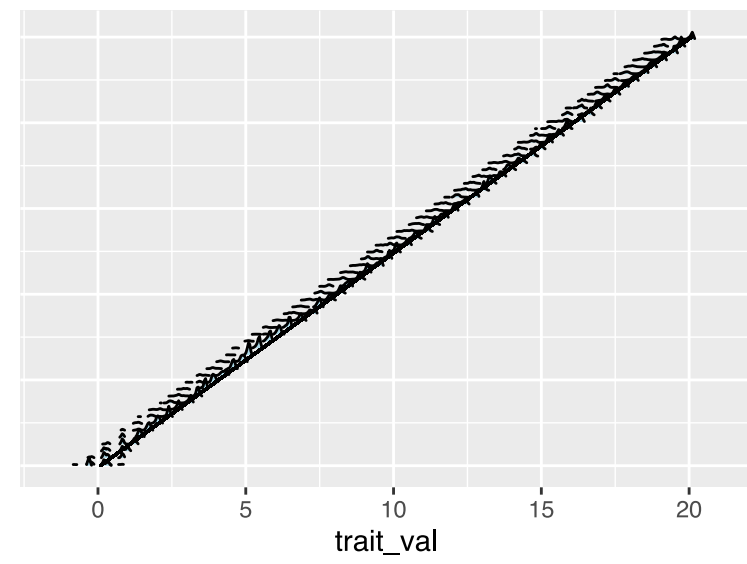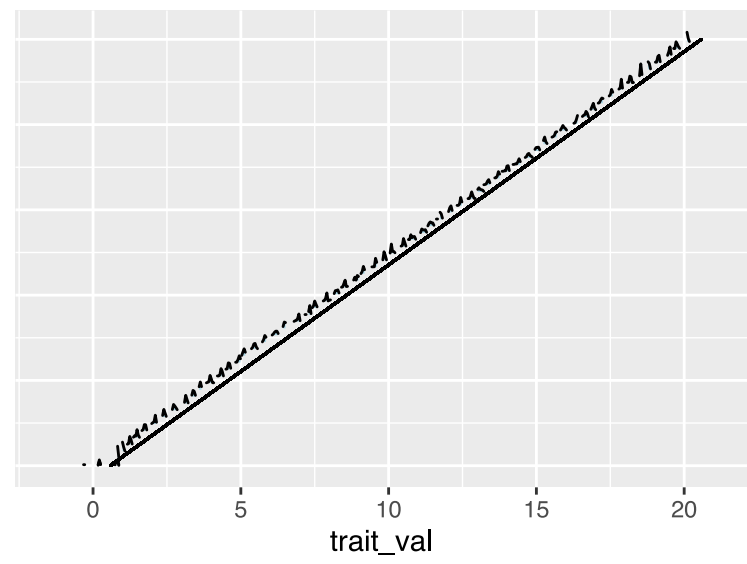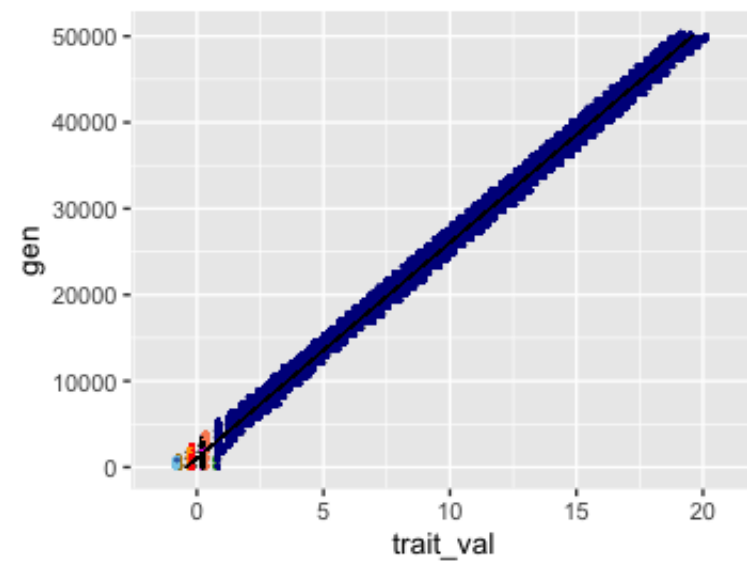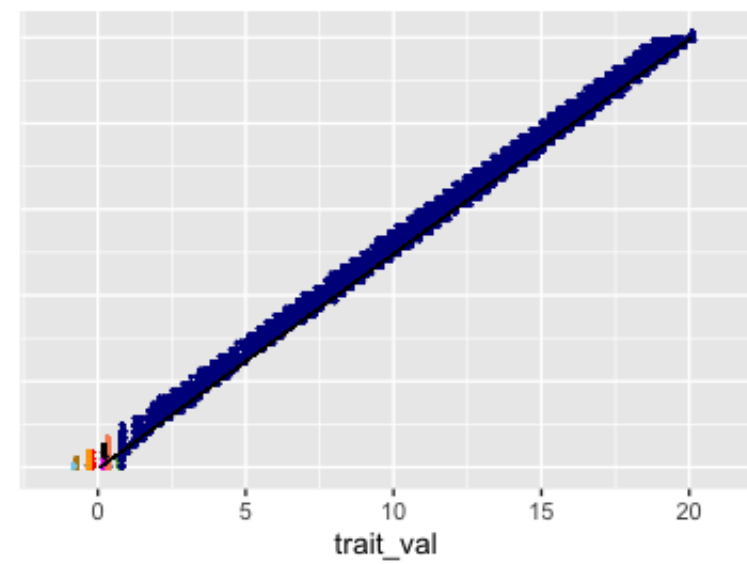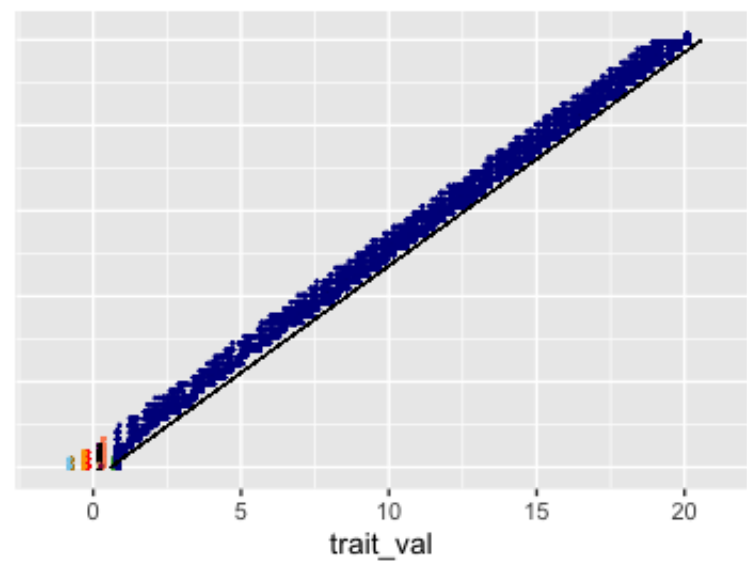

Supplement: Supplementary file 1 [file genes-11-01433-s001.zip › Figure_S15.pdf]

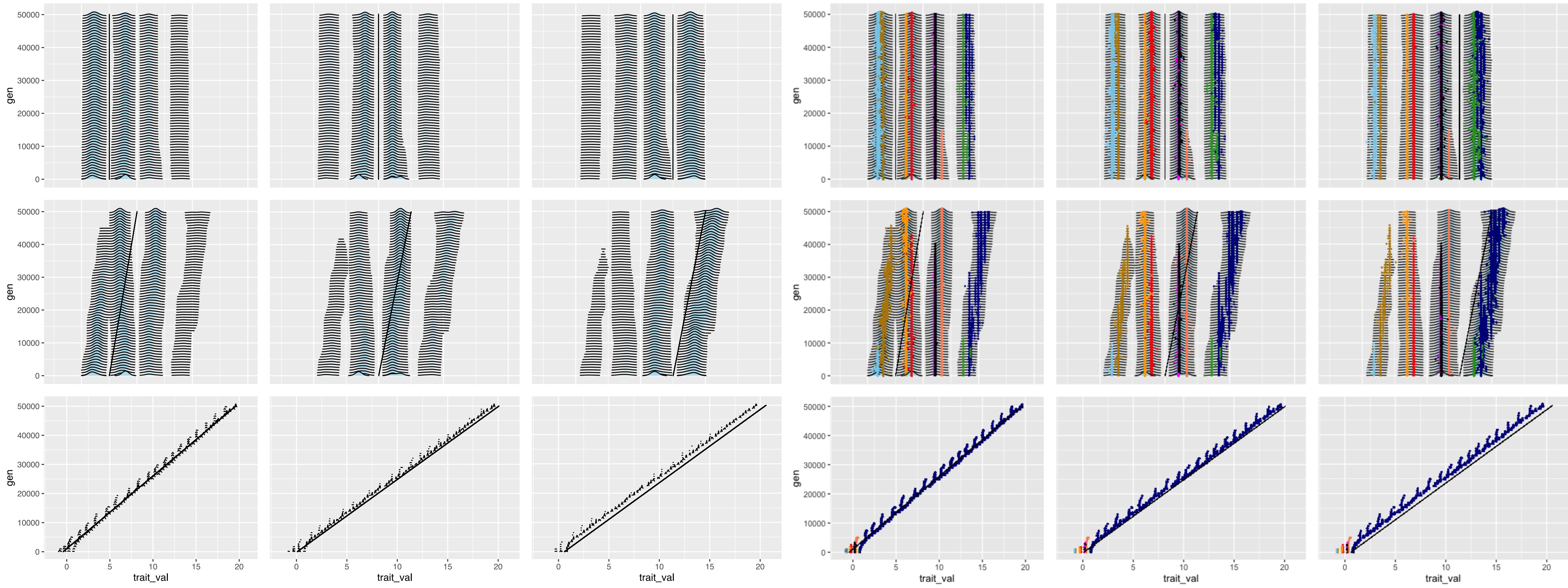

Supplement: Supplementary file 1 [file genes-11-01433-s001.zip › Figure_S16.pdf]

$\Delta=0$   
 $\sigma_\alpha=0.85, d=0$

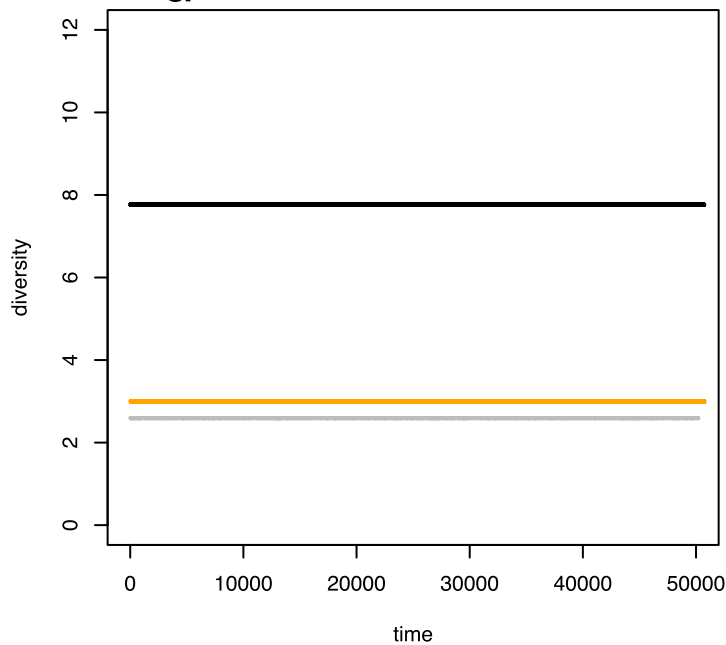

$d=0.01$

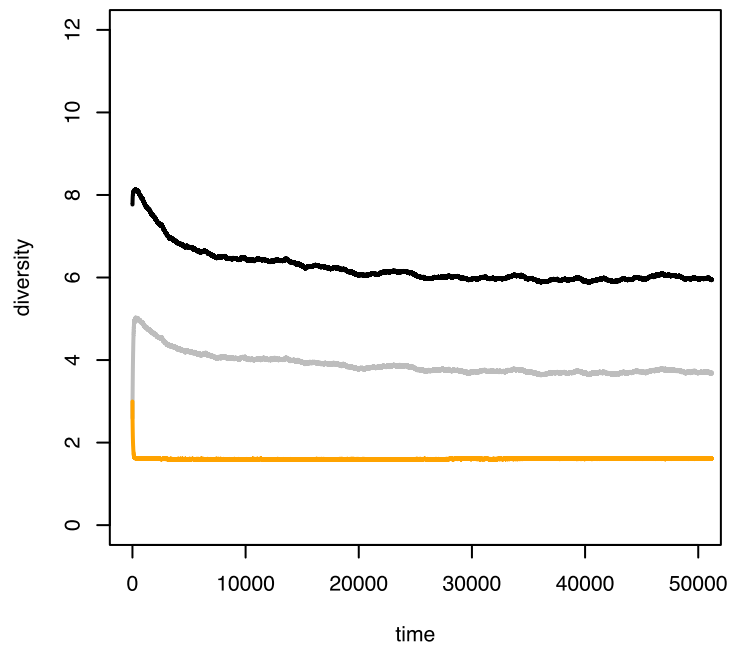

$d=0.1$

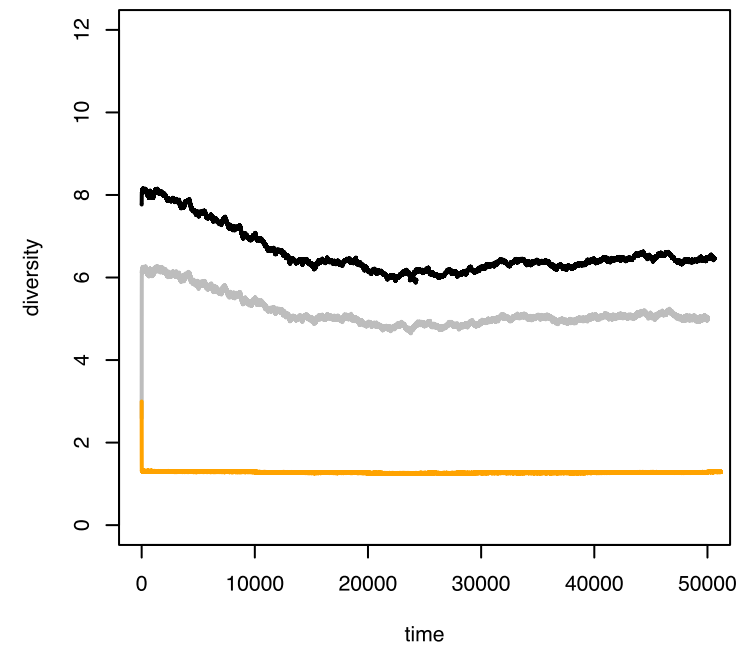

$\Delta=10^{-5}$

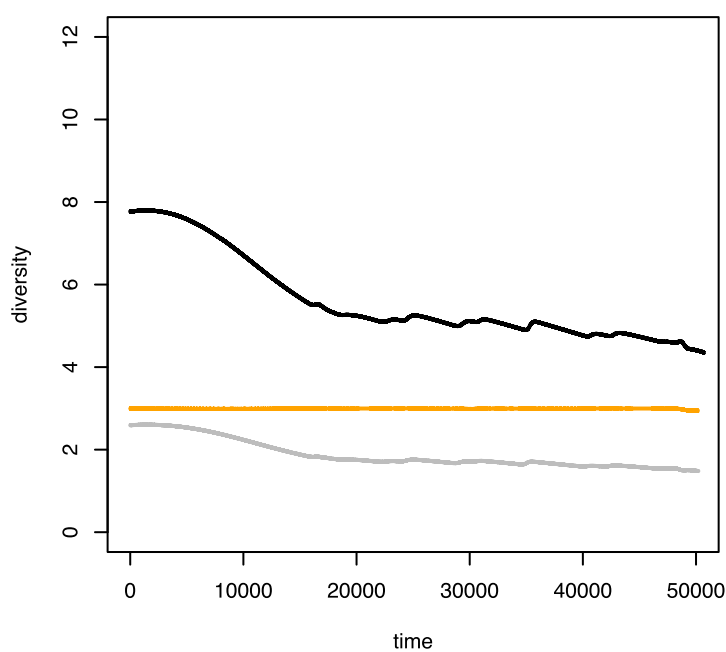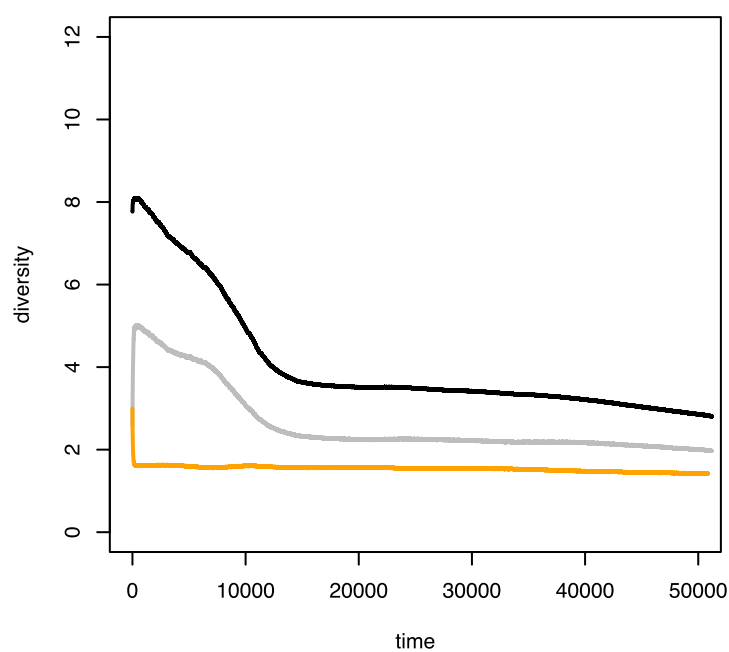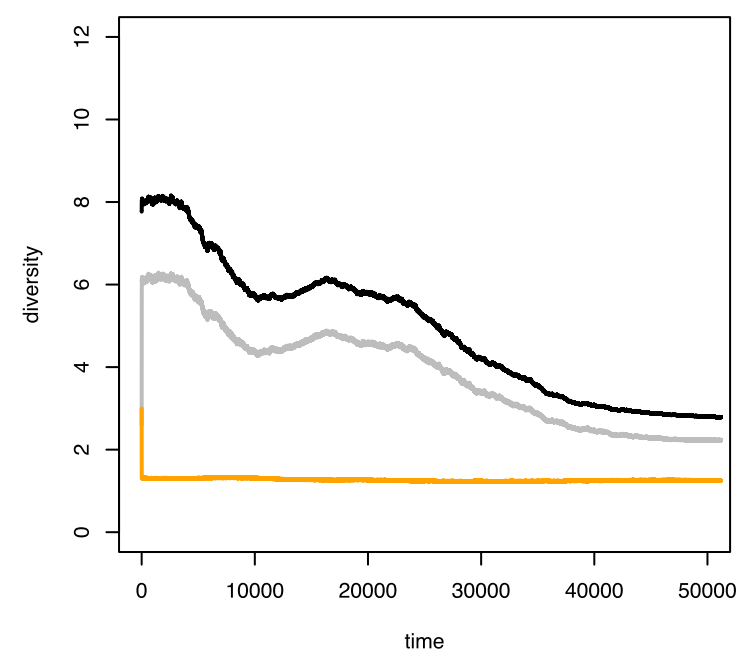

$\Delta=4.4 \times 10^{-4}$

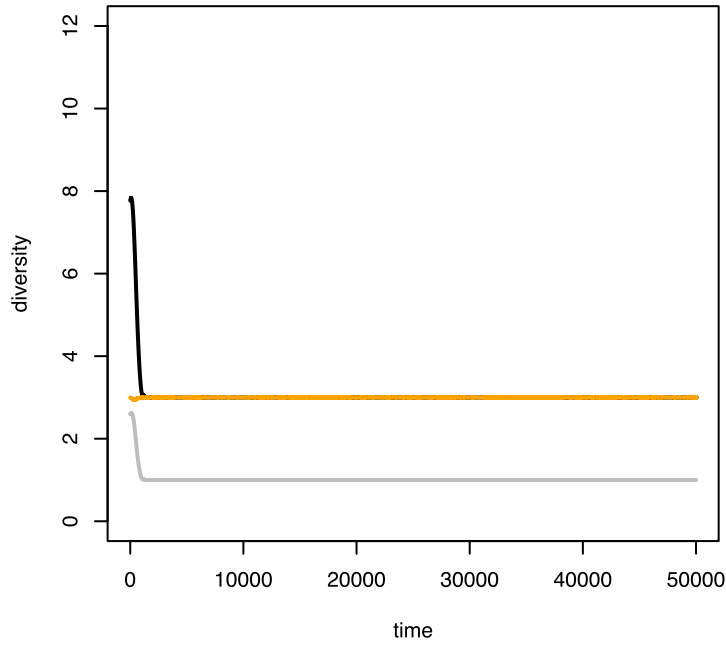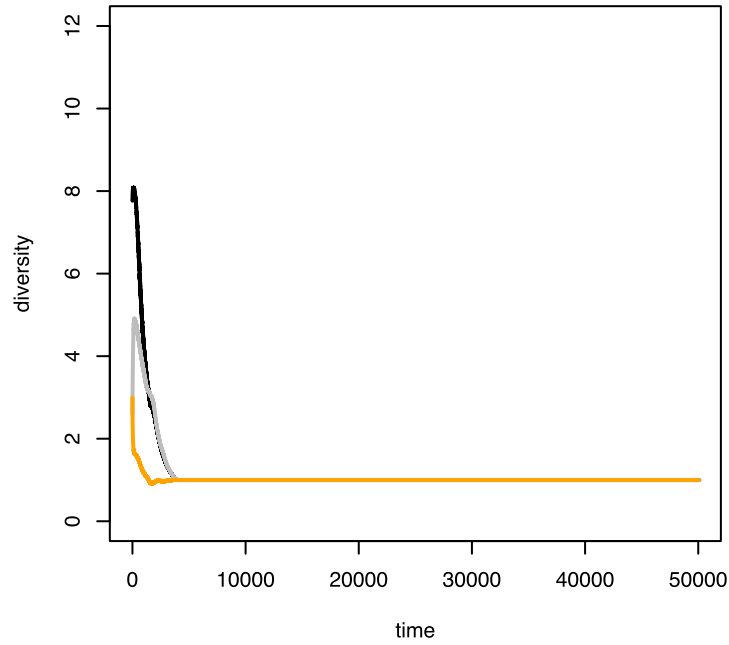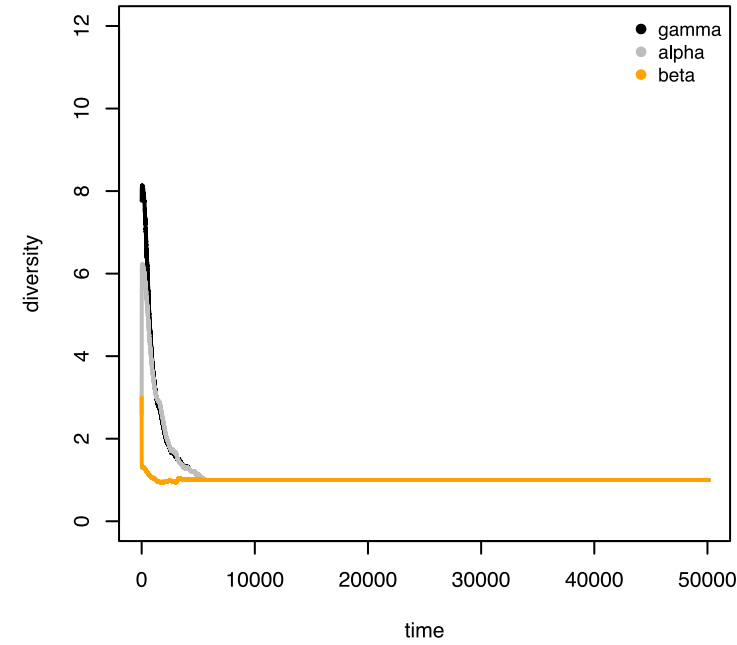

● gamma  
● alpha  
● beta

Supplement: Supplementary file 1 [file genes-11-01433-s001.zip › Figure_S17.pdf]

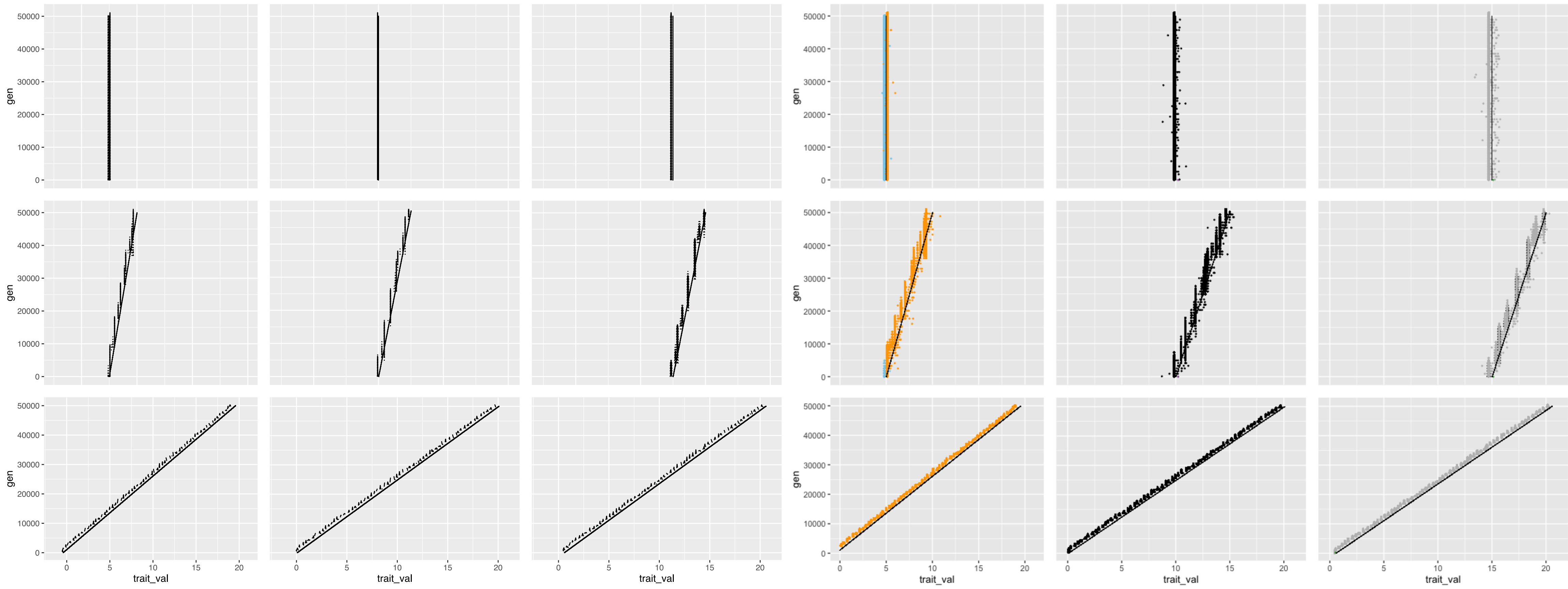

Supplement: Supplementary file 1 [file genes-11-01433-s001.zip › Figure_S18.pdf]

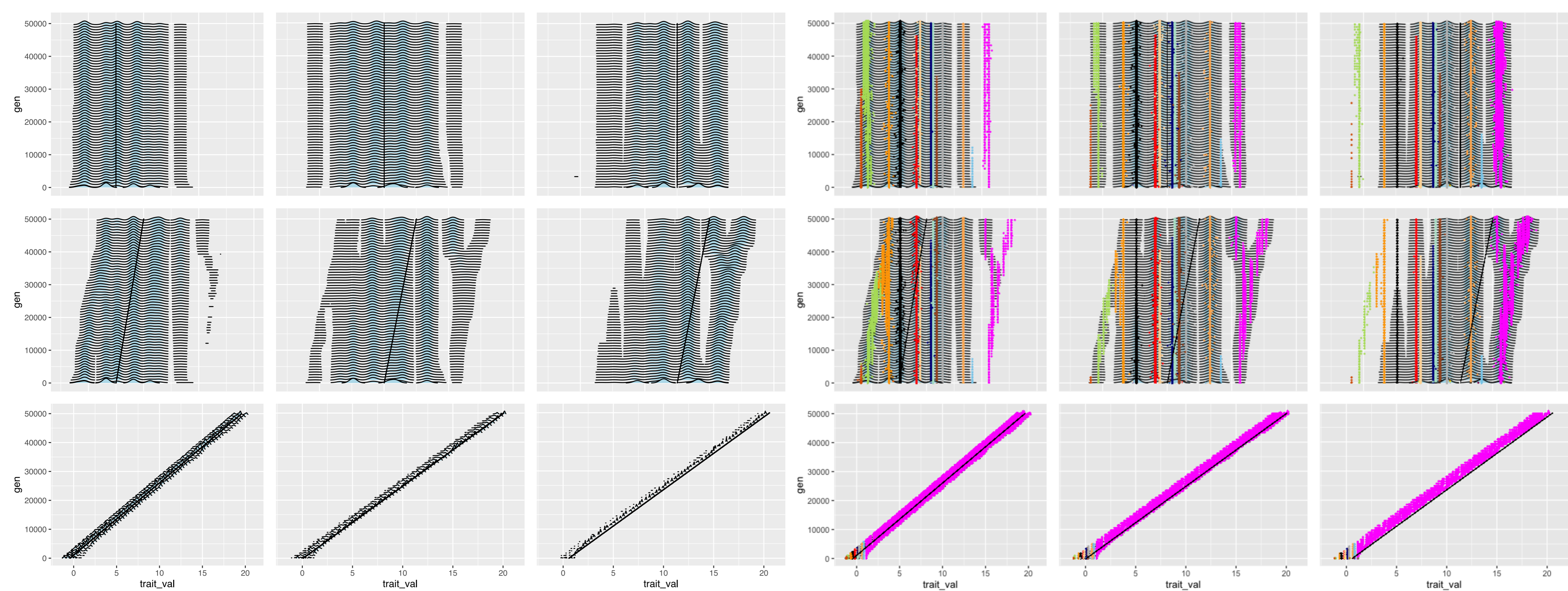

Supplement: Supplementary file 1 [file genes-11-01433-s001.zip › Figure_S2.pdf]

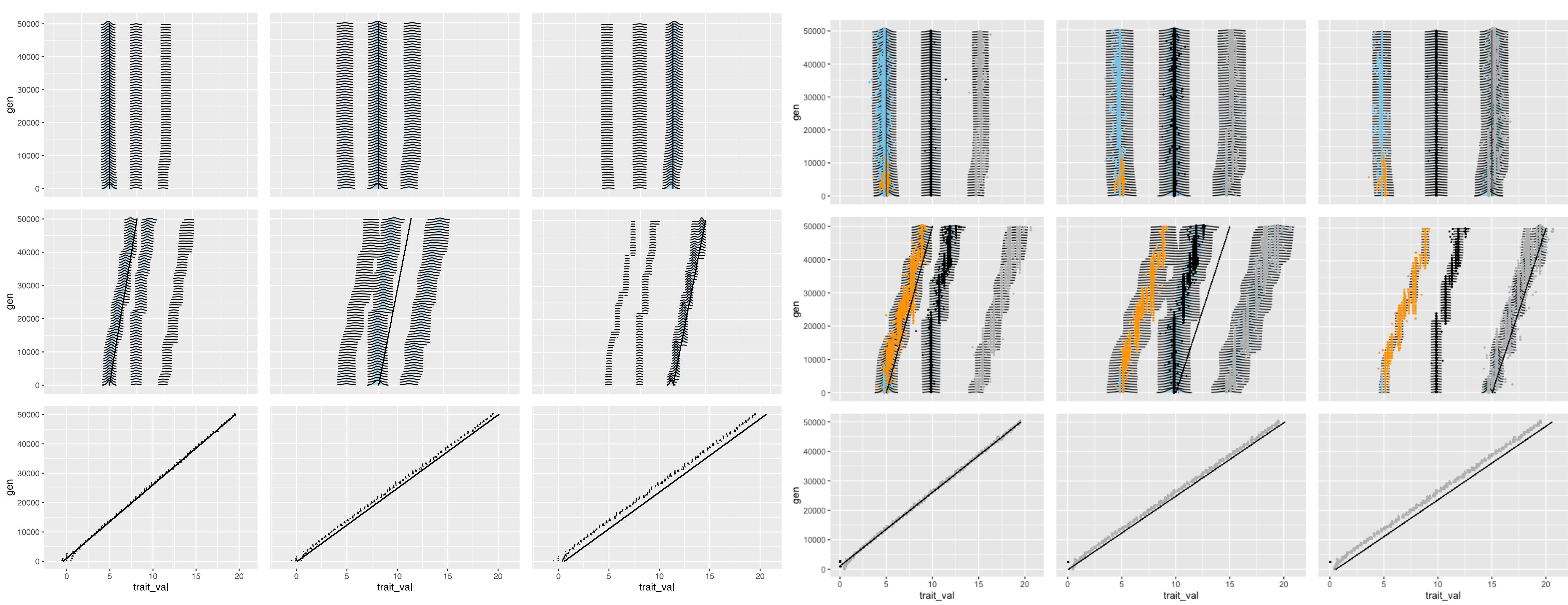

Supplement: Supplementary file 1 [file genes-11-01433-s001.zip › Figure_S20.pdf]

$\Delta=0$   
 $\sigma_\alpha=1.5, d=0$

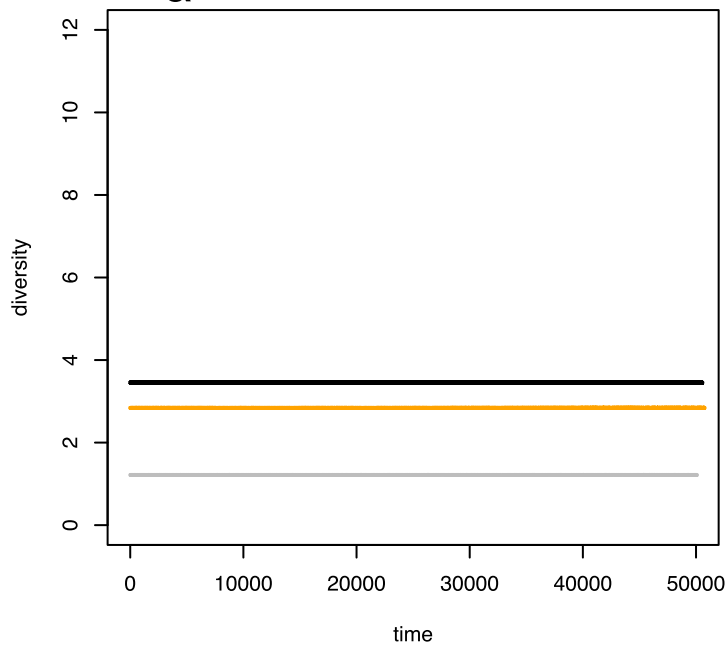

$d=0.01$

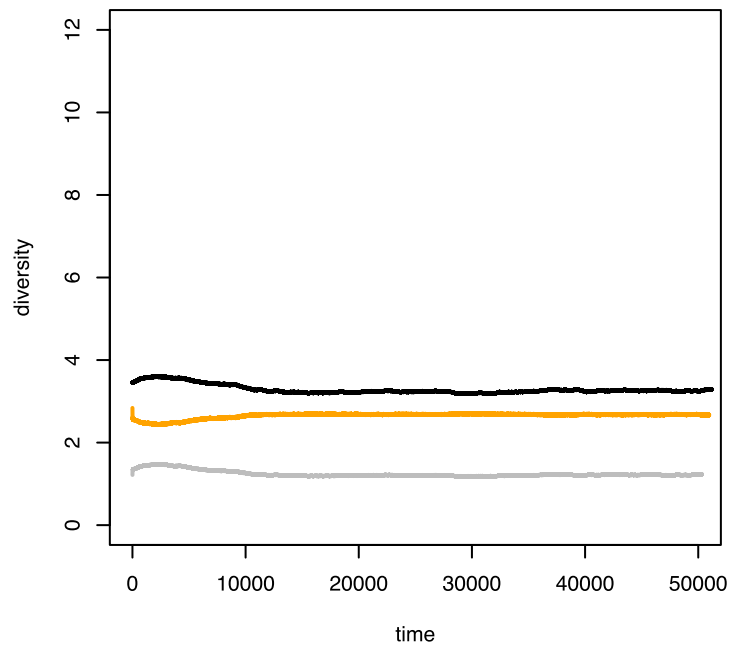

$d=0.1$

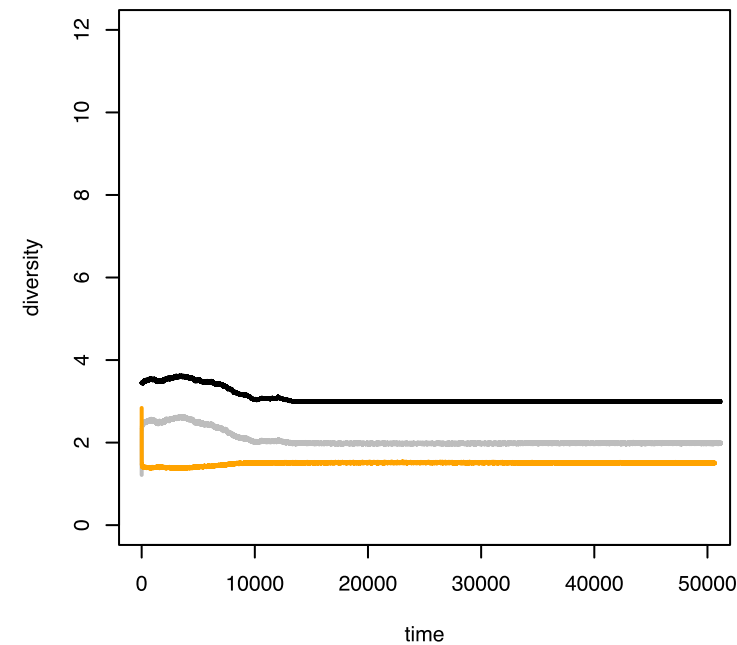

$\Delta=10^{-5}$

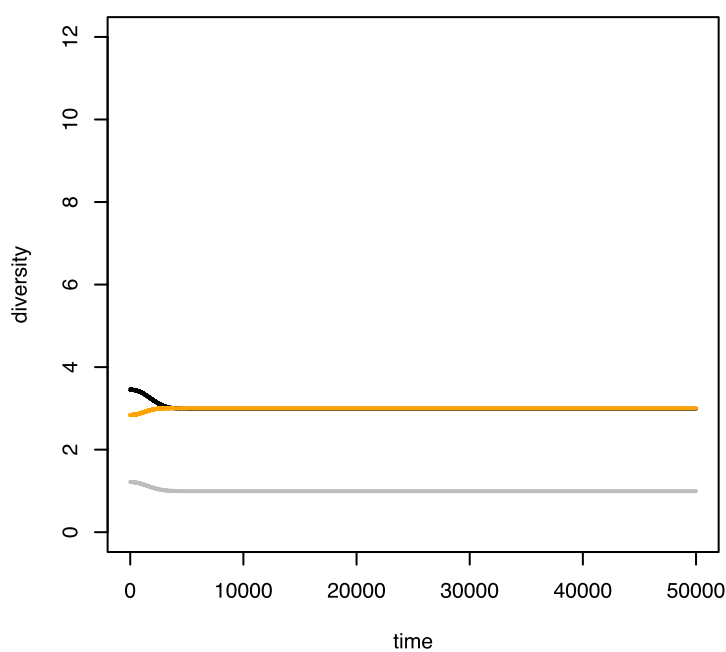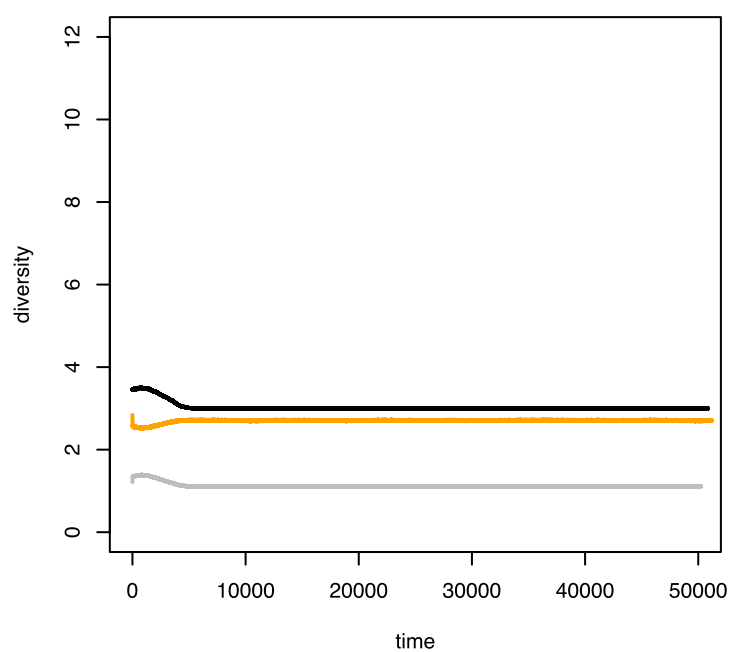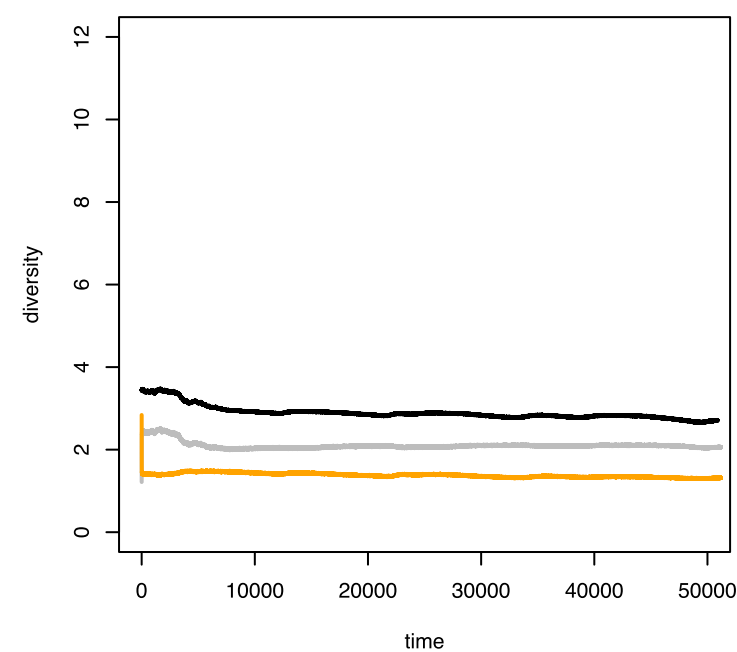

$\Delta=4.4 \times 10^{-4}$

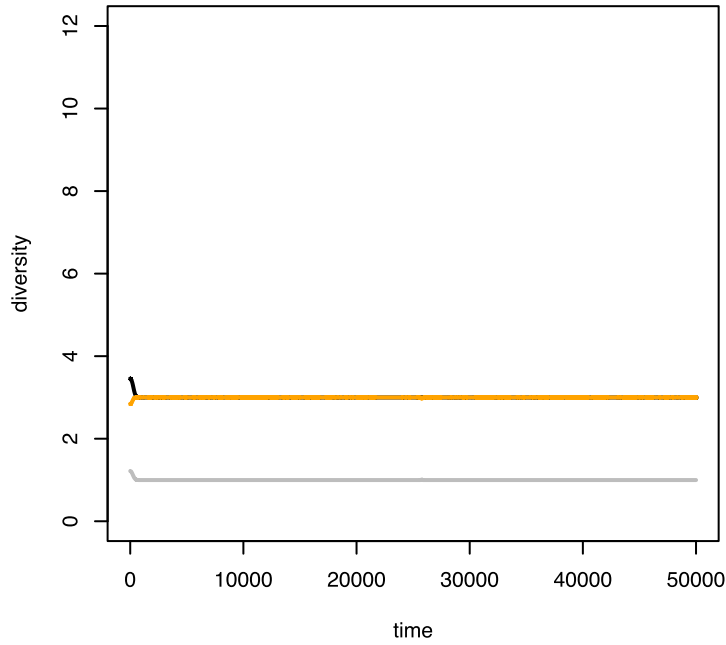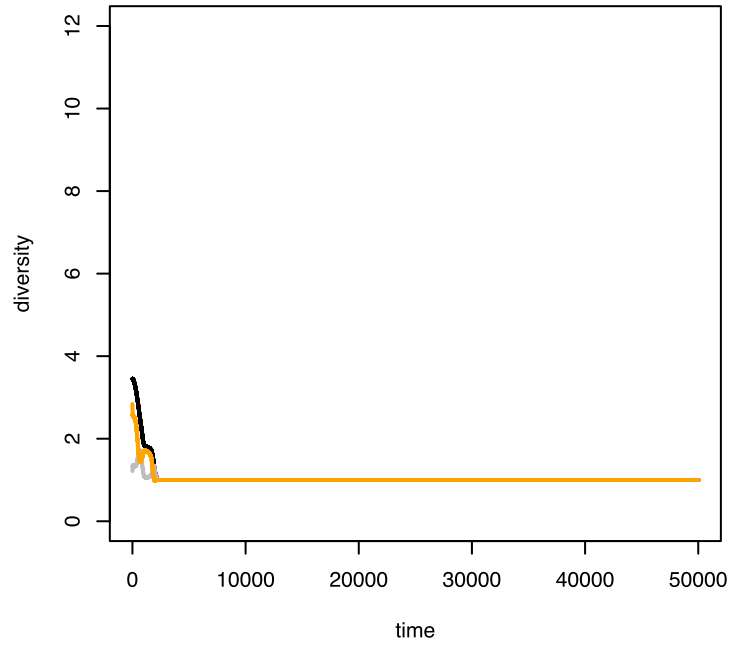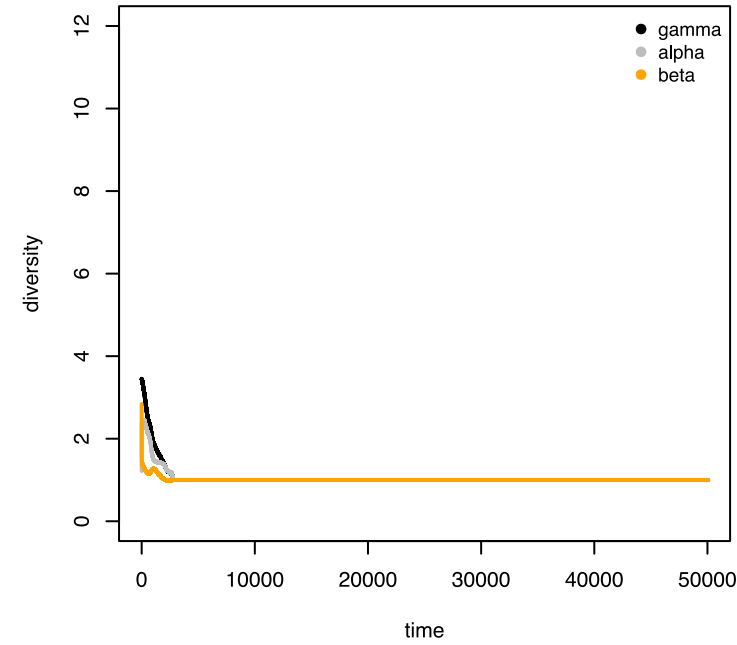

Supplement: Supplementary file 1 [file genes-11-01433-s001.zip › Figure_S21.pdf]

$d = 0$

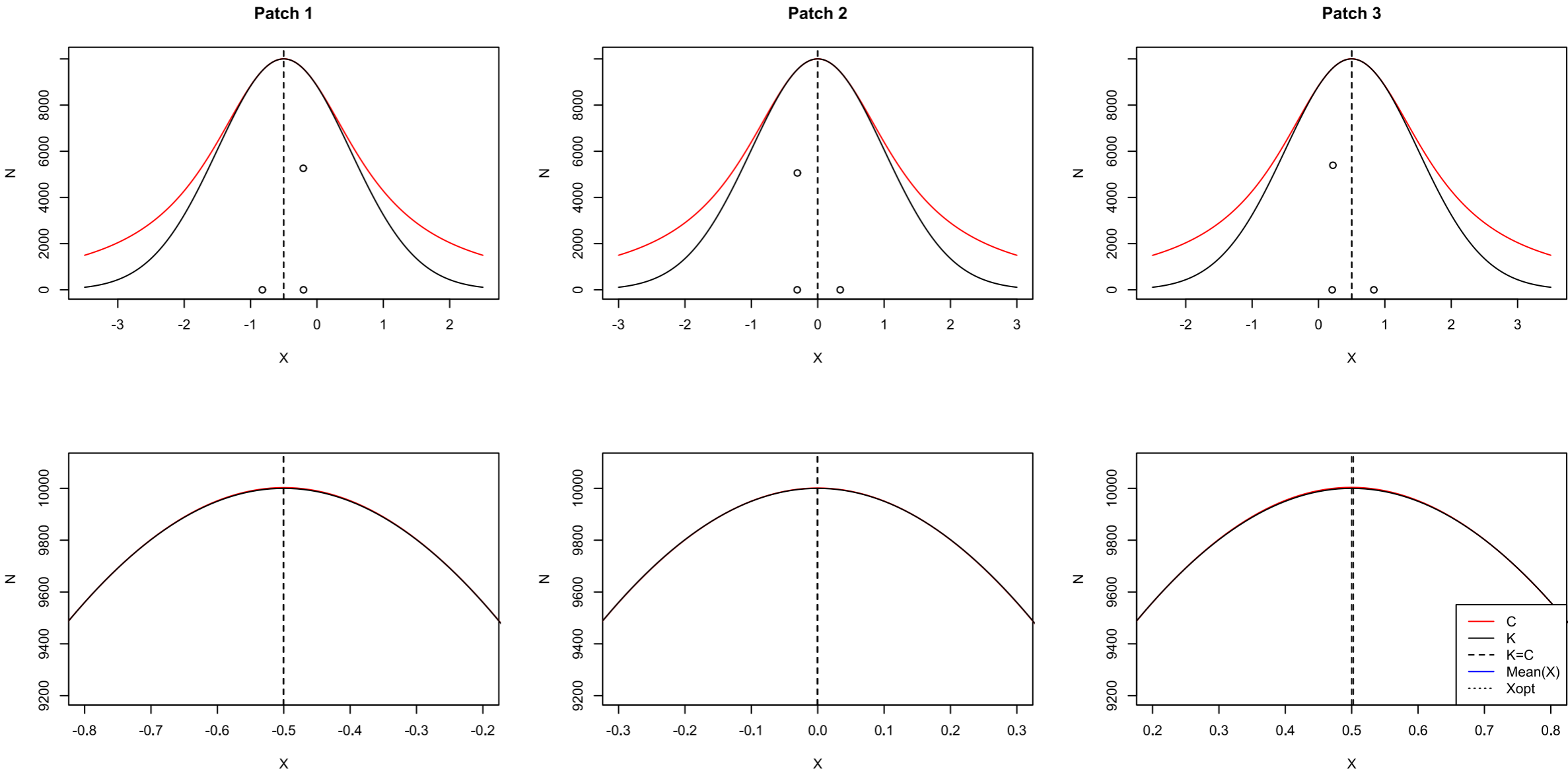

$d = 0.01$

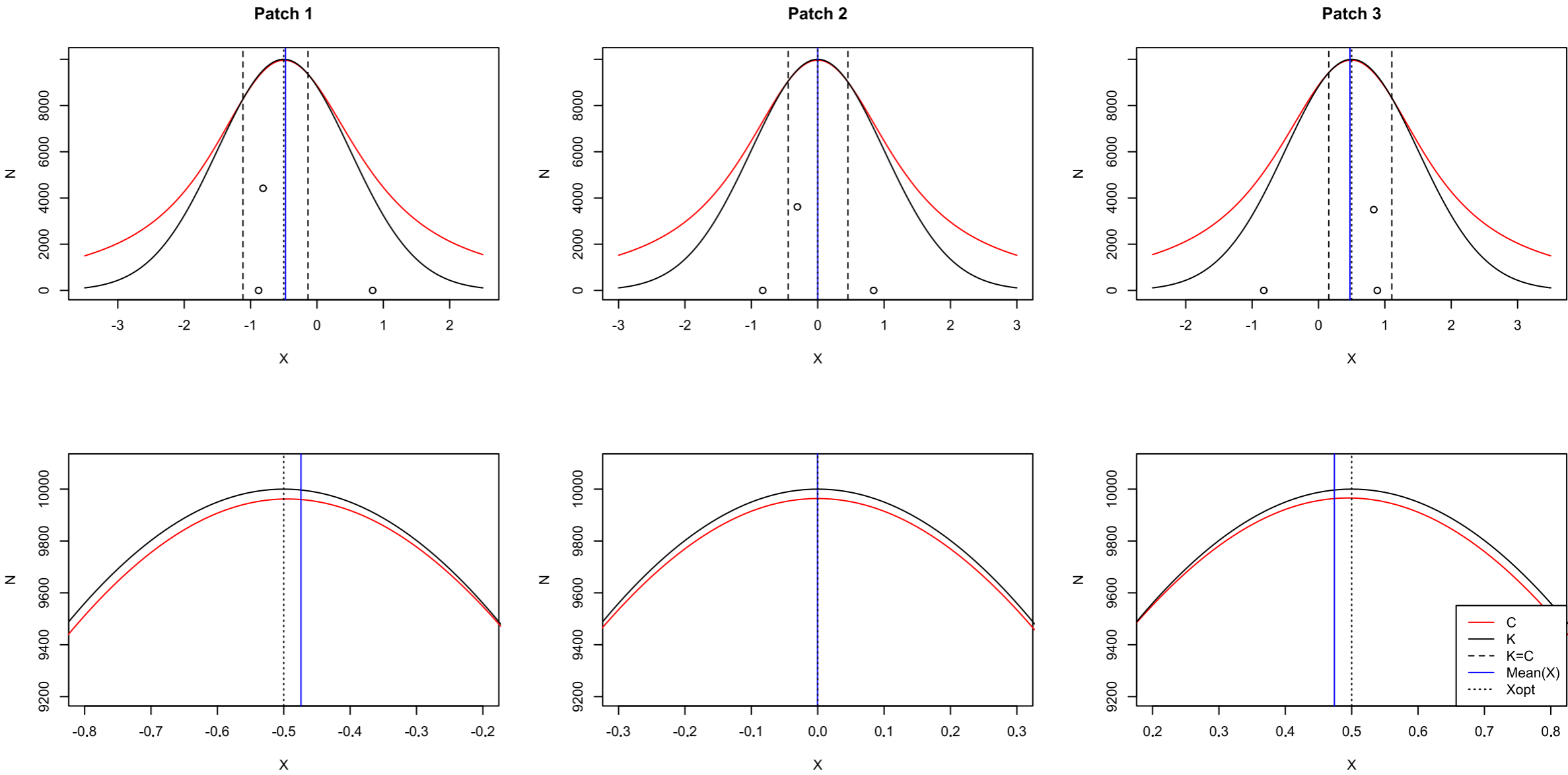

$d = 0.1$

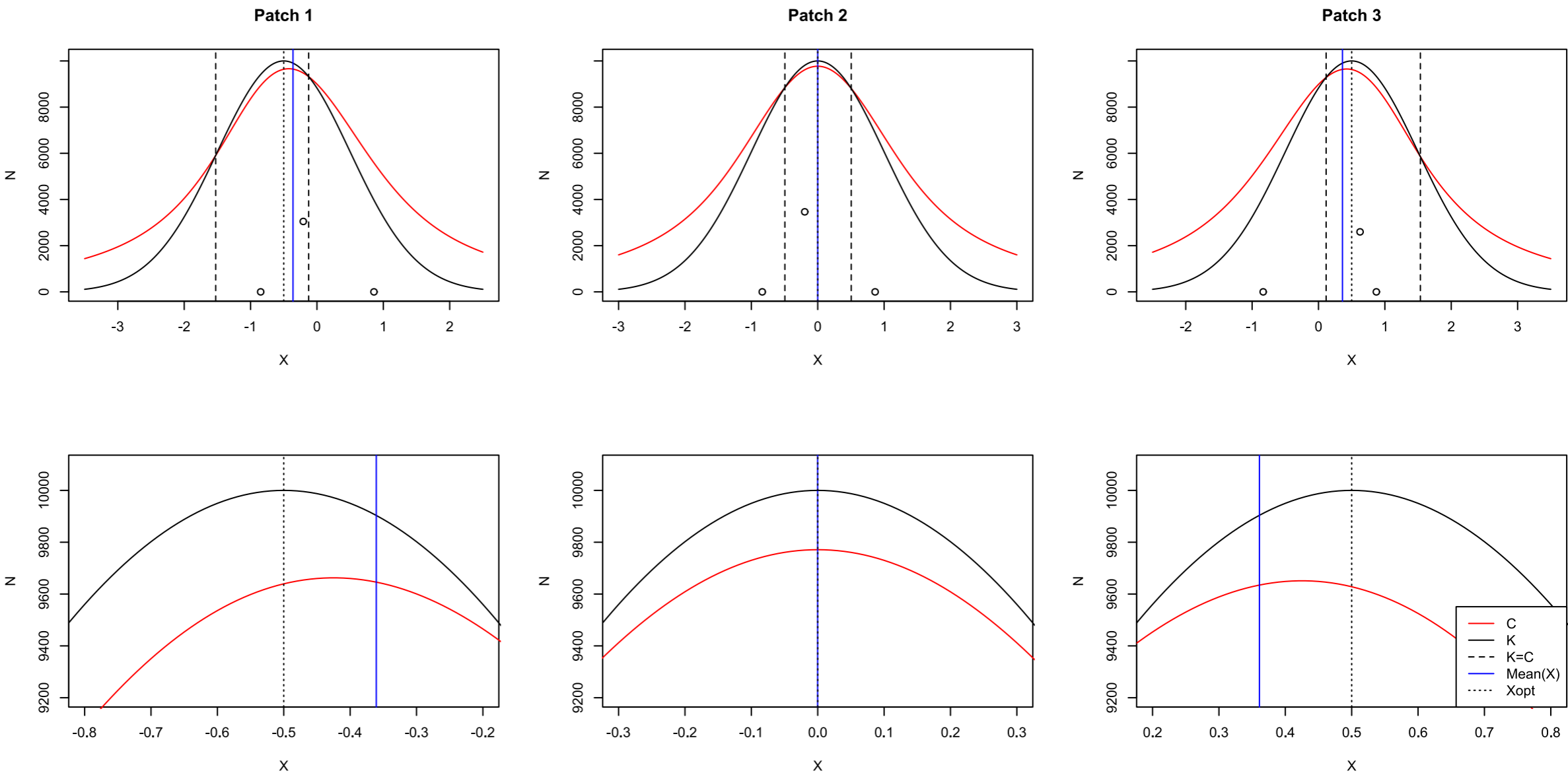

Supplement: Supplementary file 1 [file genes-11-01433-s001.zip › Figure_S22.pdf]

$\Delta=0$   
 $\sigma_\alpha=0.85, d=0$

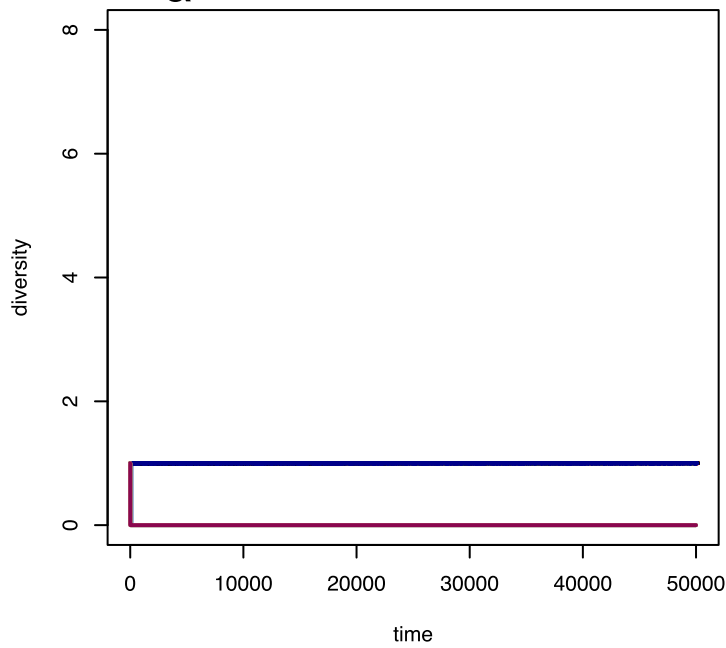

$d=0.01$

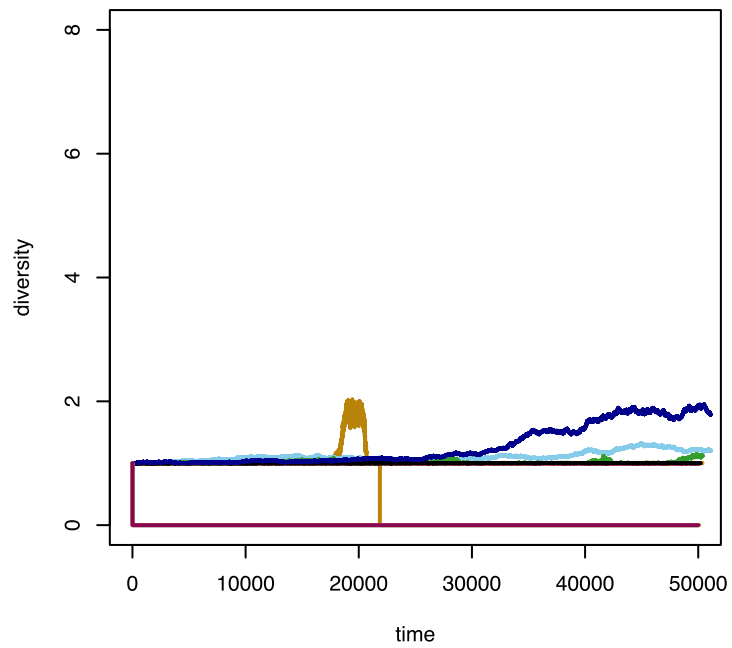

$d=0.1$

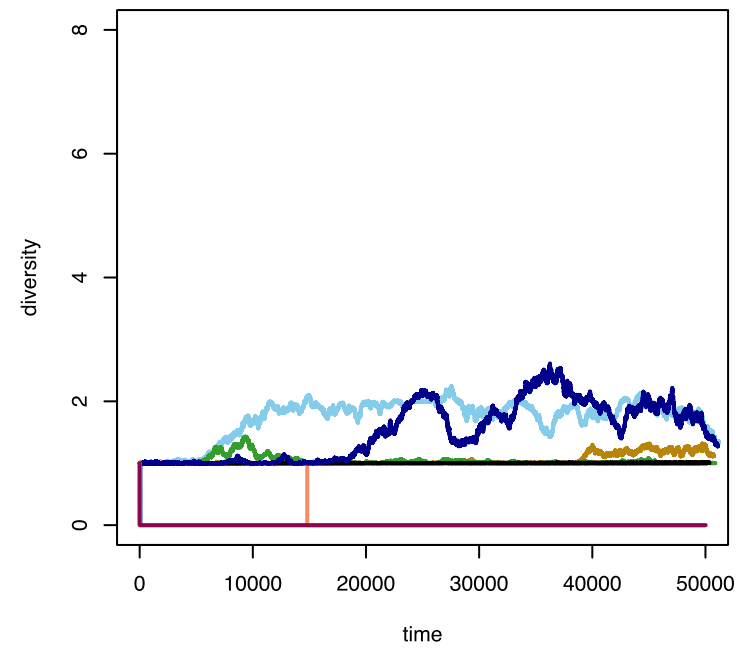

$\Delta=10^{-5}$

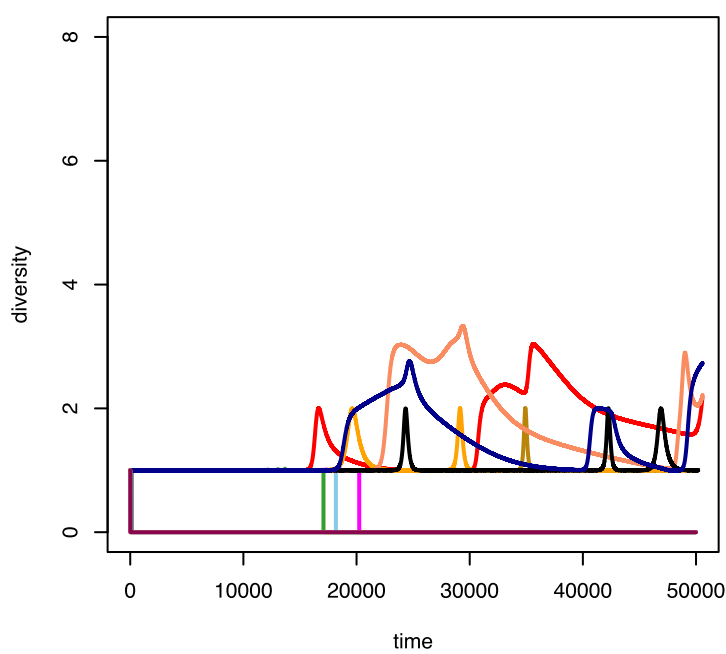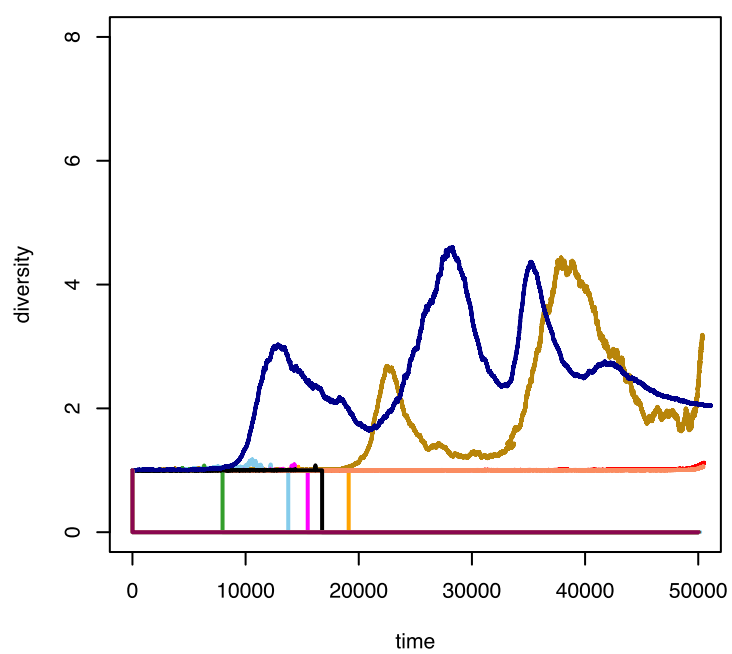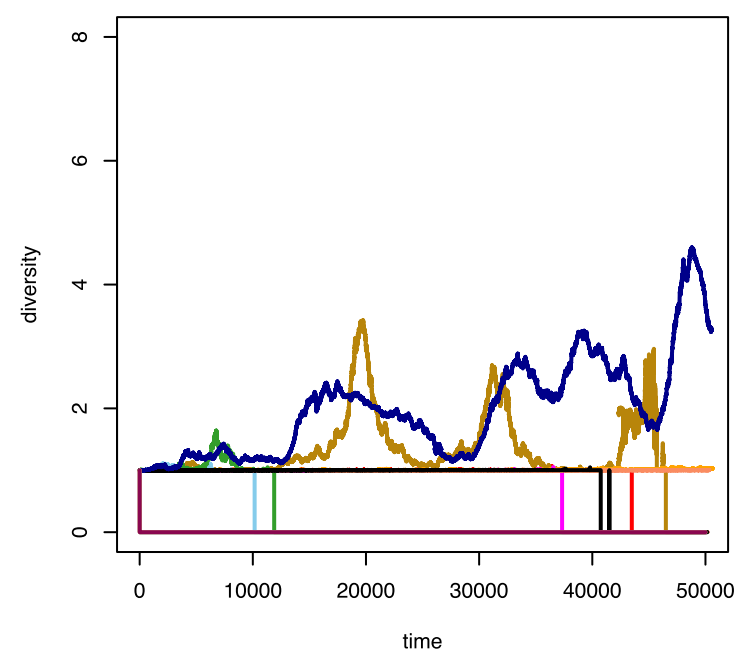

$\Delta=4.4 \times 10^{-4}$

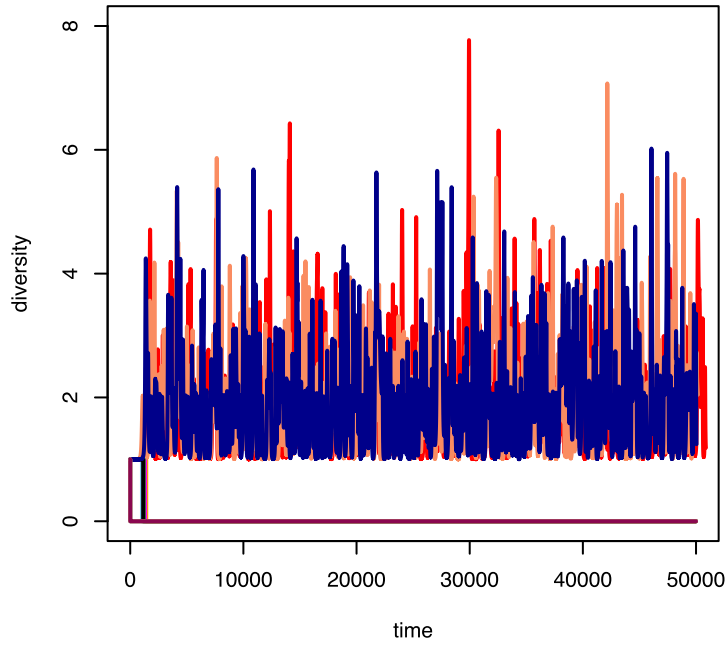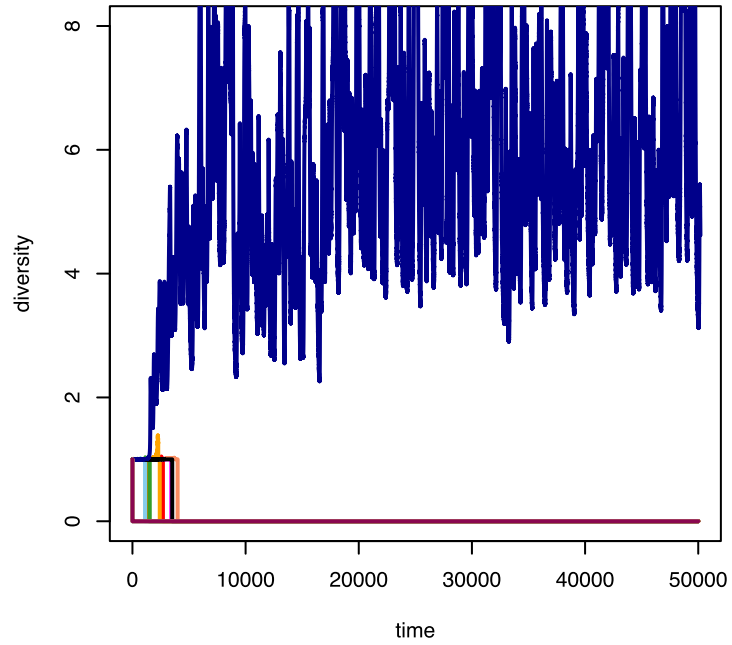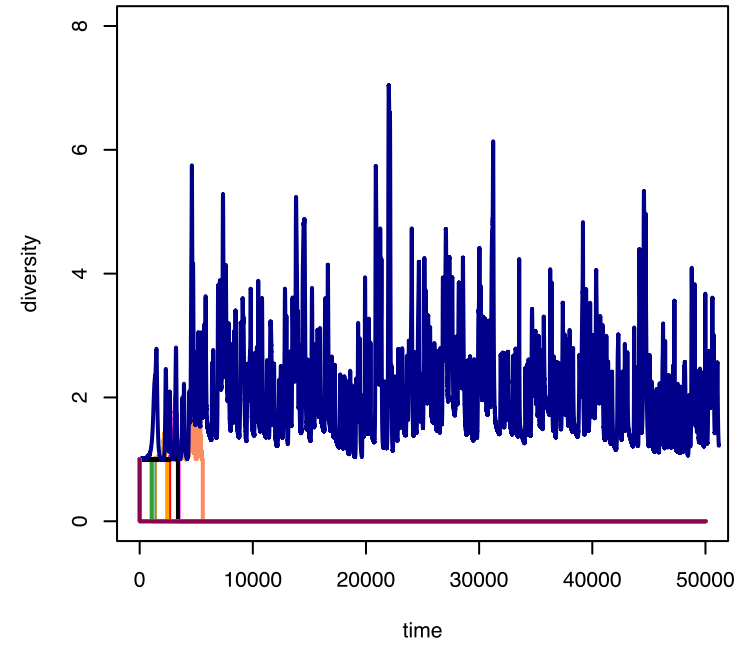

Supplement: Supplementary file 1 [file genes-11-01433-s001.zip › Figure_S23.pdf]

$\Delta=0$   
 $\sigma_\alpha=1.5, d=0$

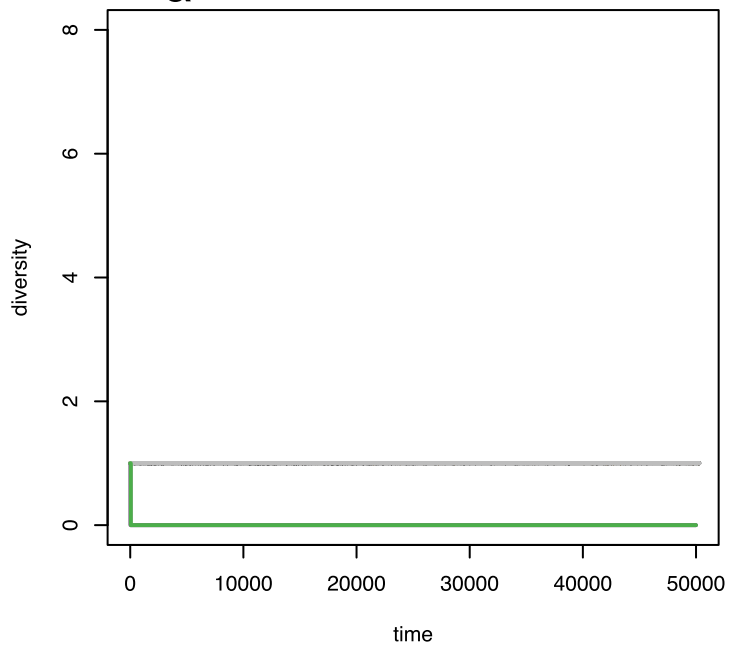

$d=0.01$

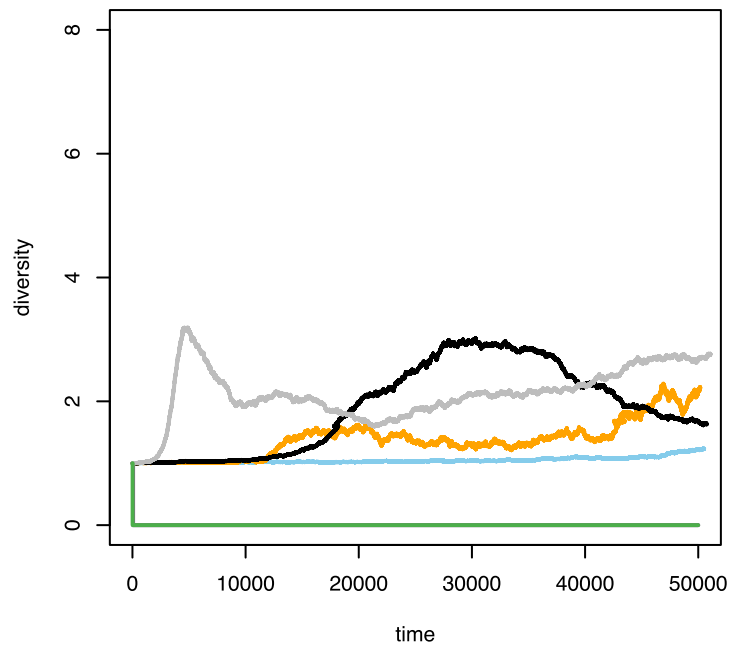

$d=0.1$

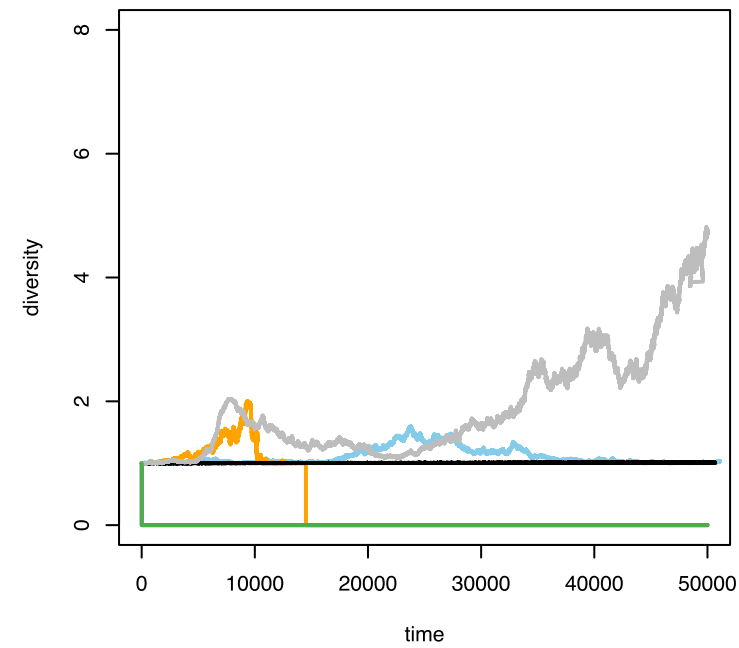

$\Delta=10^{-5}$

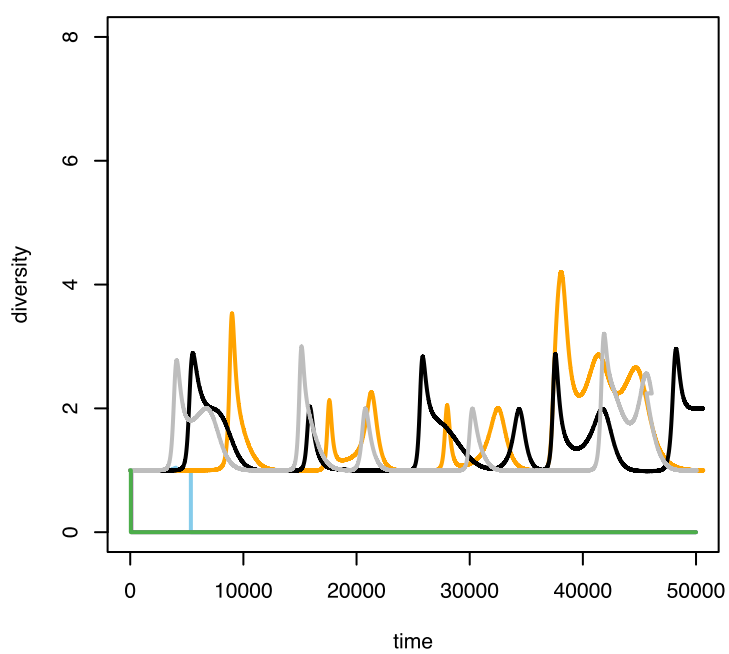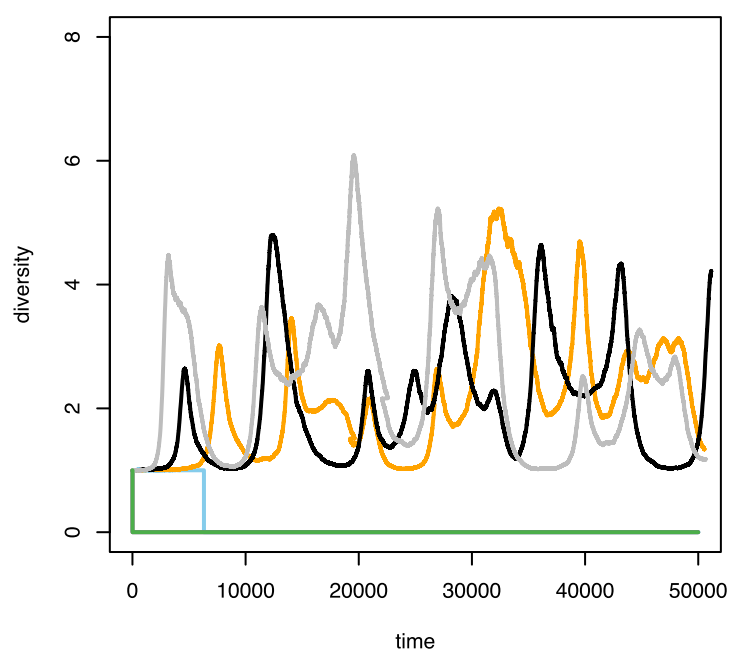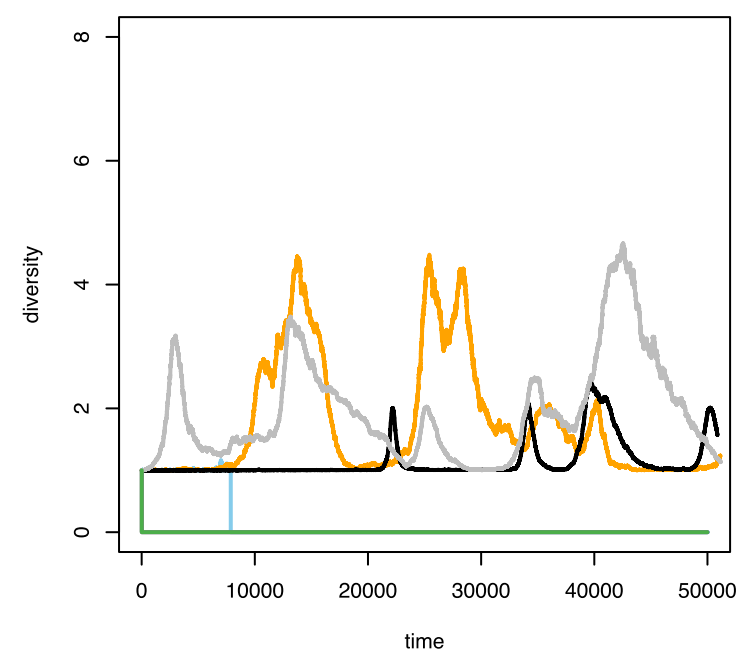

$\Delta=4.4 \times 10^{-4}$

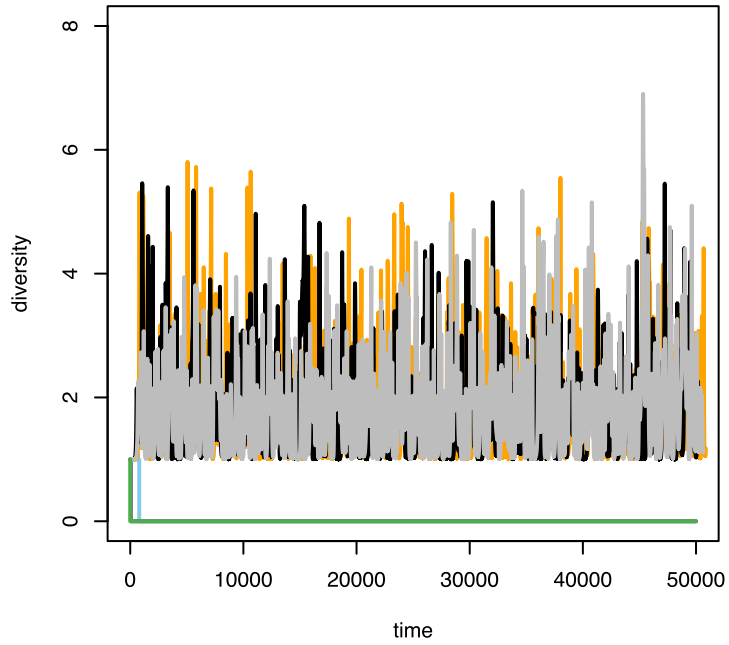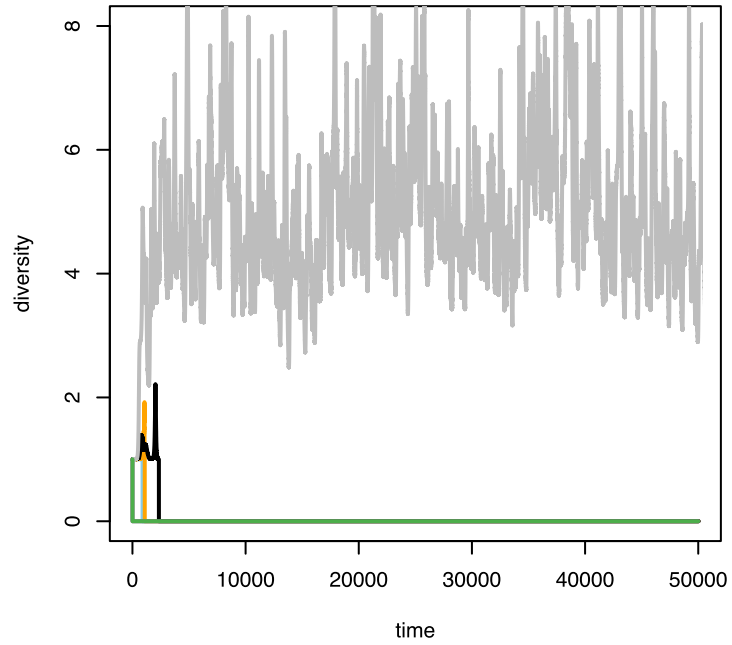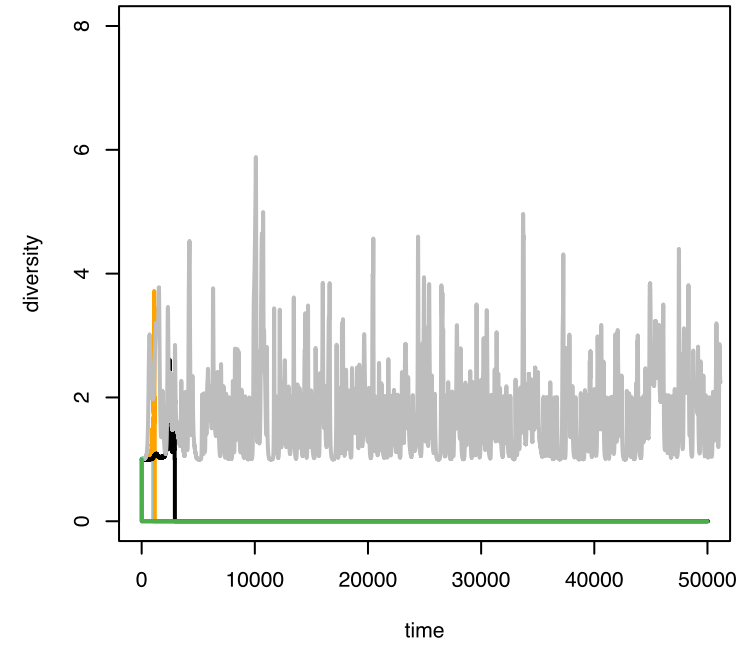

Supplement: Supplementary file 1 [file genes-11-01433-s001.zip › Figure_S24.pdf]

$d = 0$

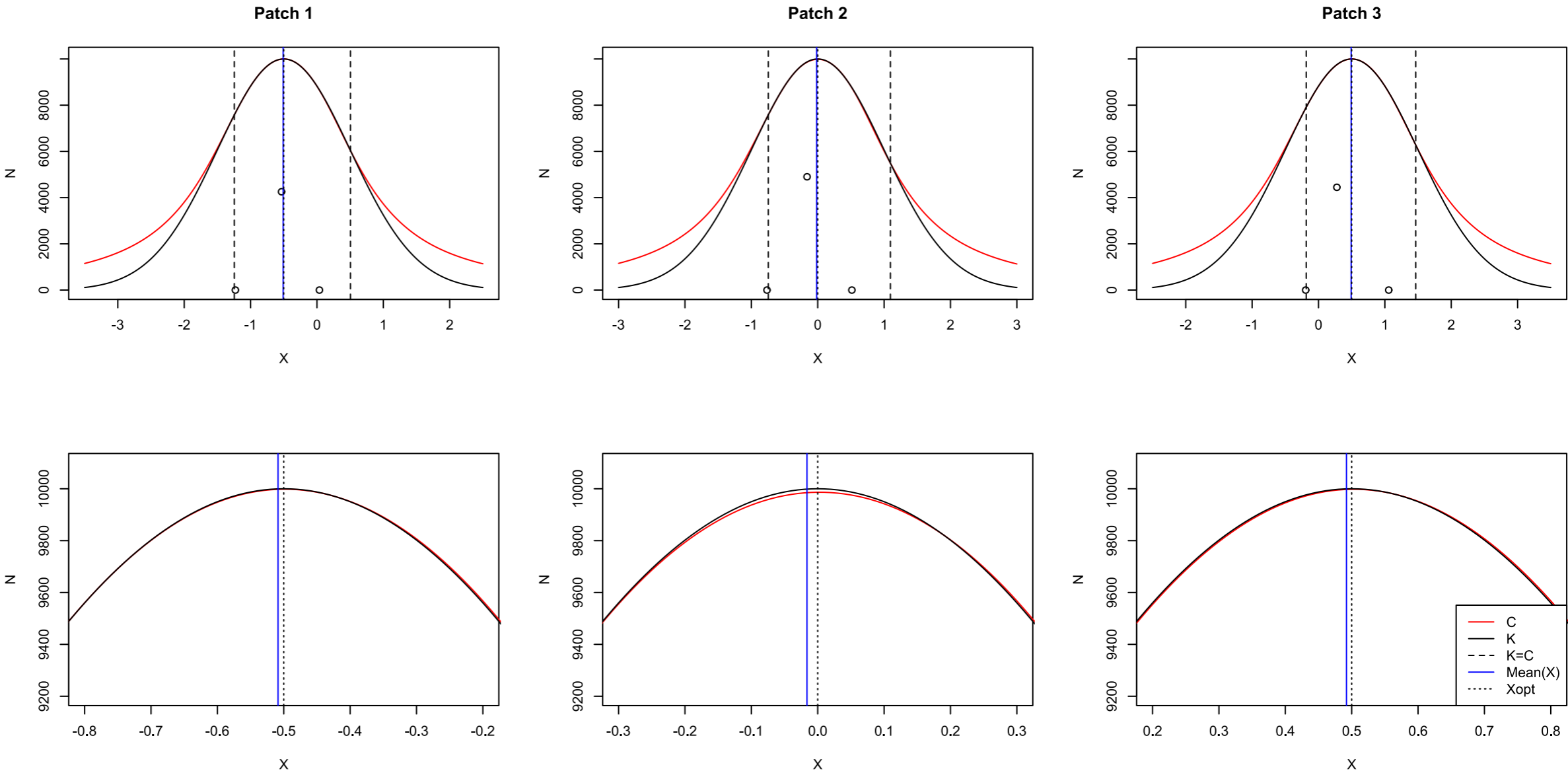

$d = 0.01$

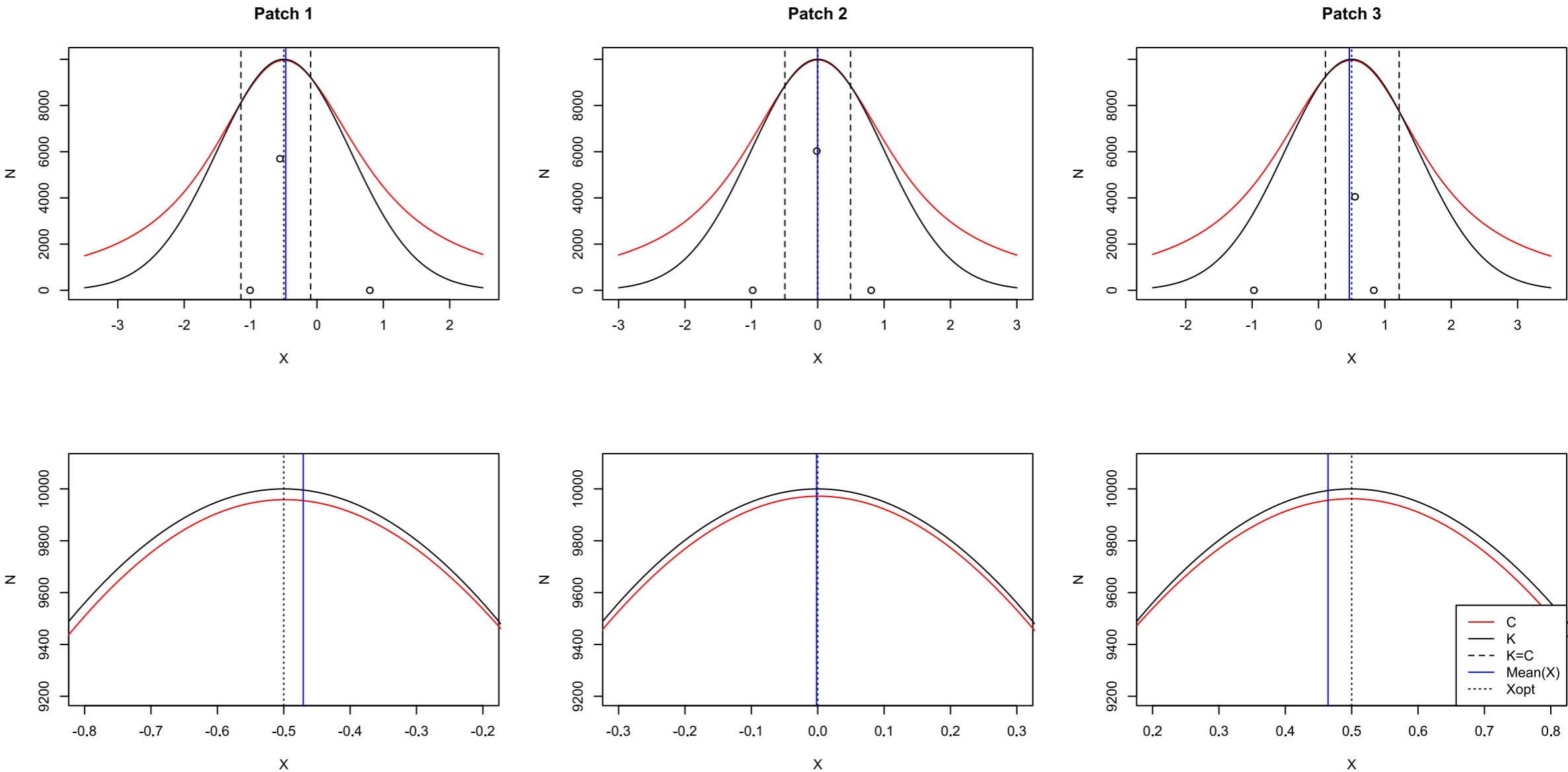

$d = 0.1$

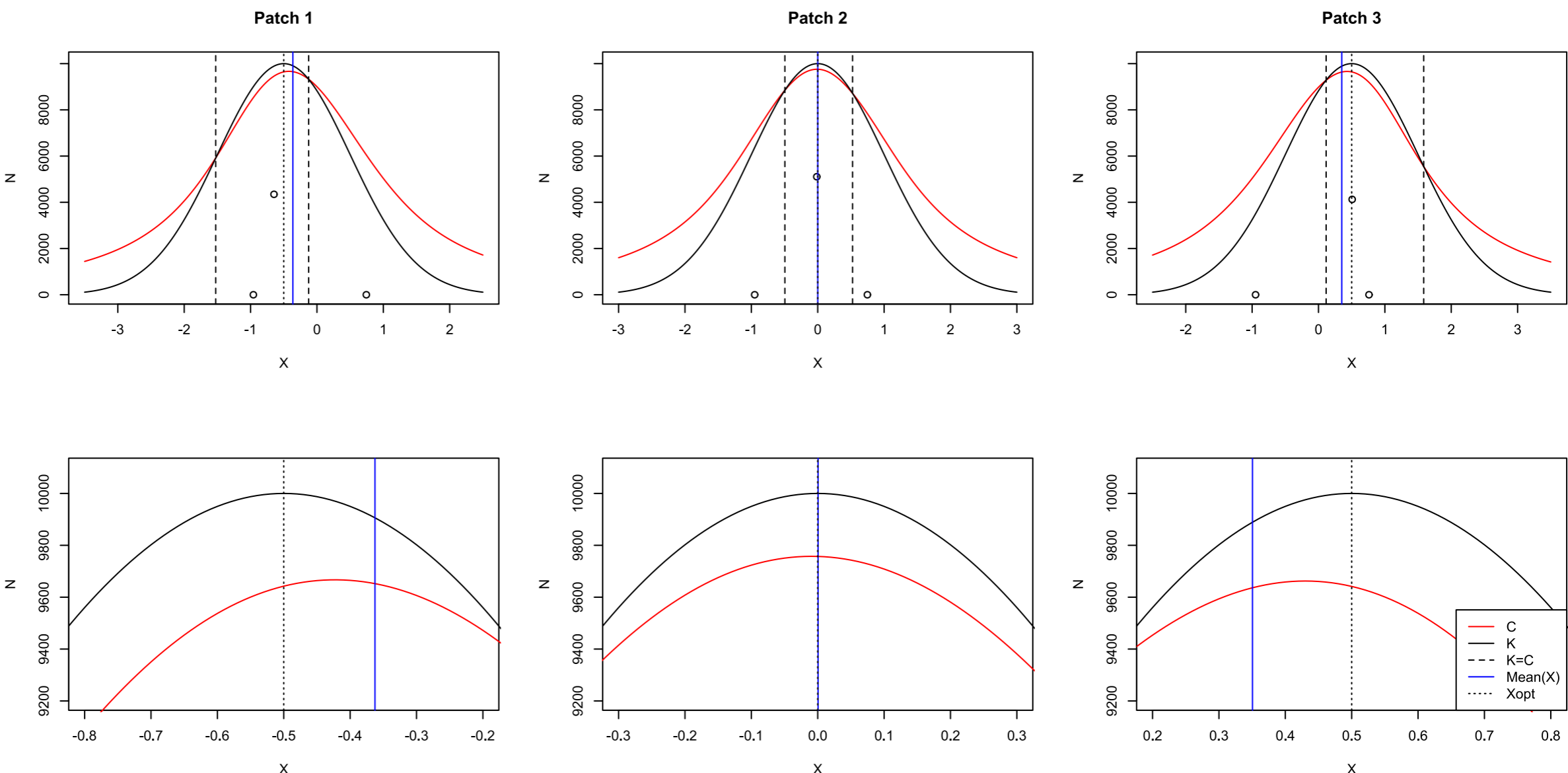

Supplement: Supplementary file 1 [file genes-11-01433-s001.zip › Figure_S25.pdf]

$\Delta=0$   
 $\sigma_\alpha=0.85, d=0$

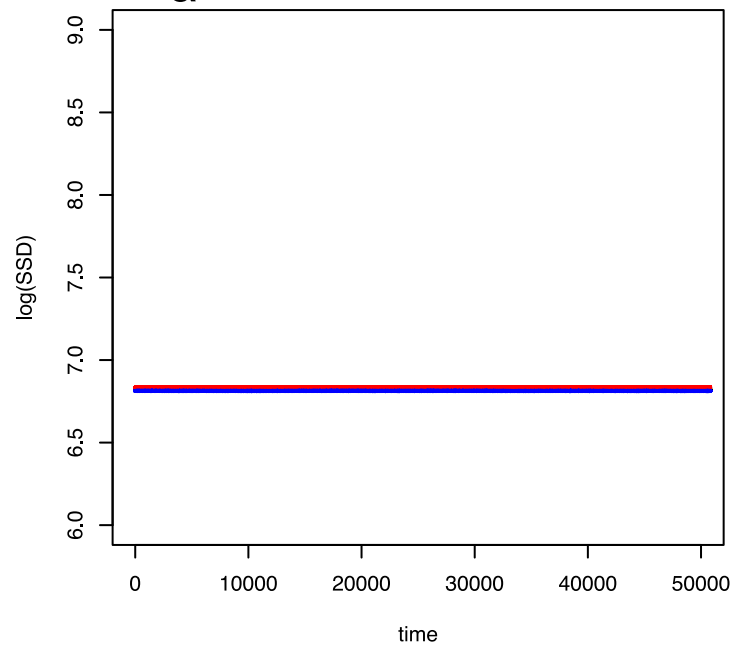

$d=0.01$

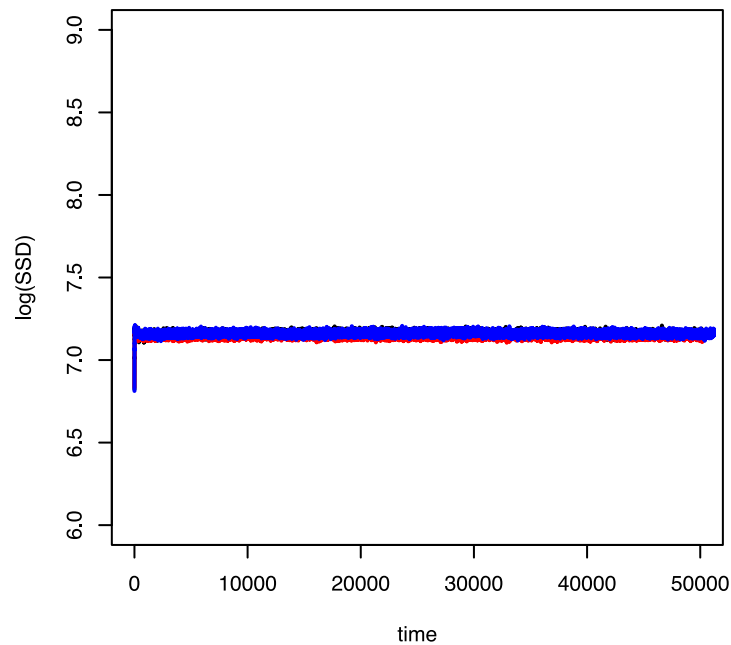

$d=0.1$

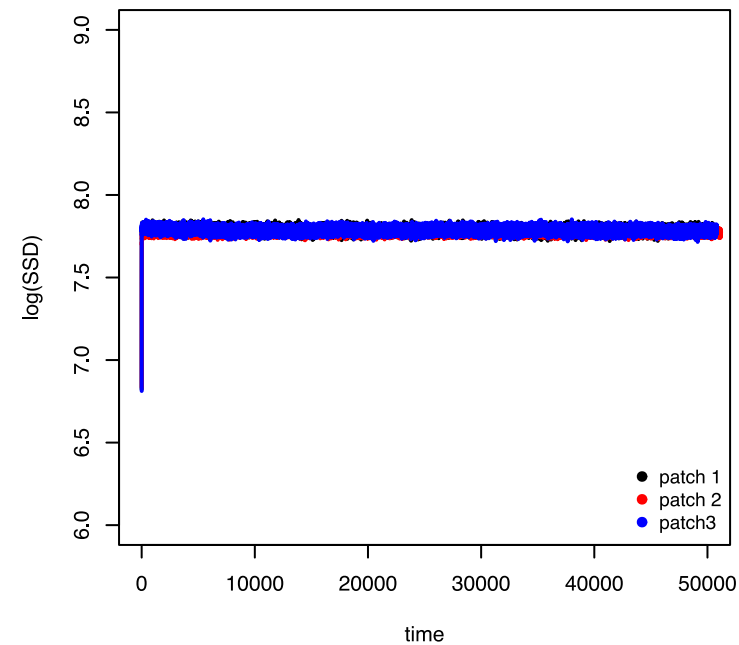

$\Delta=10^{-5}$

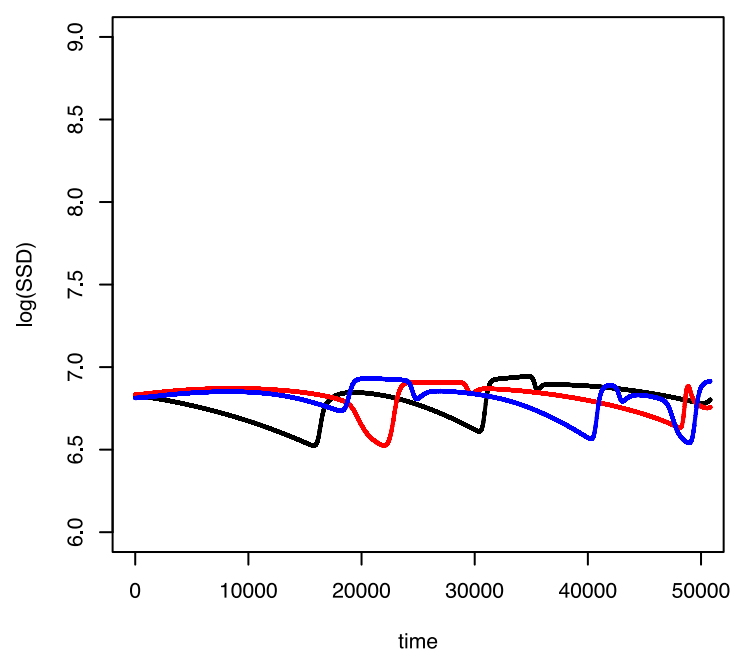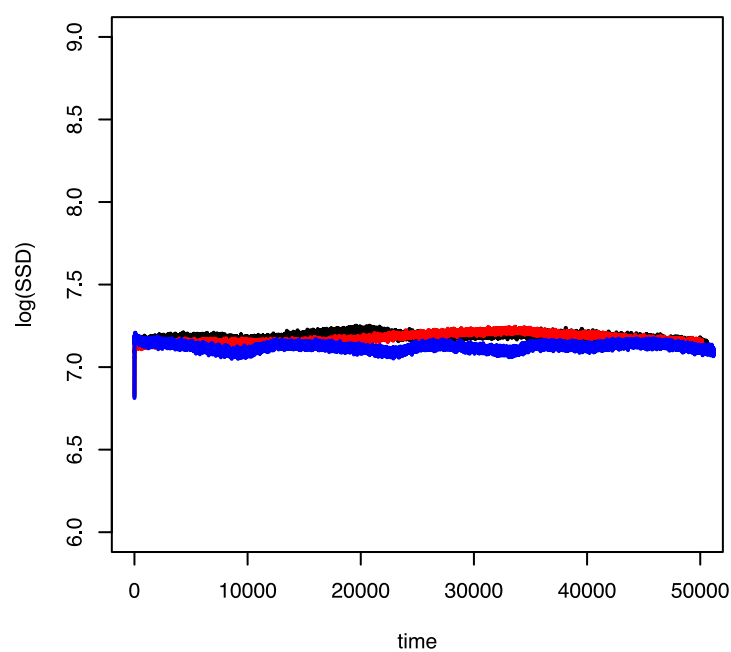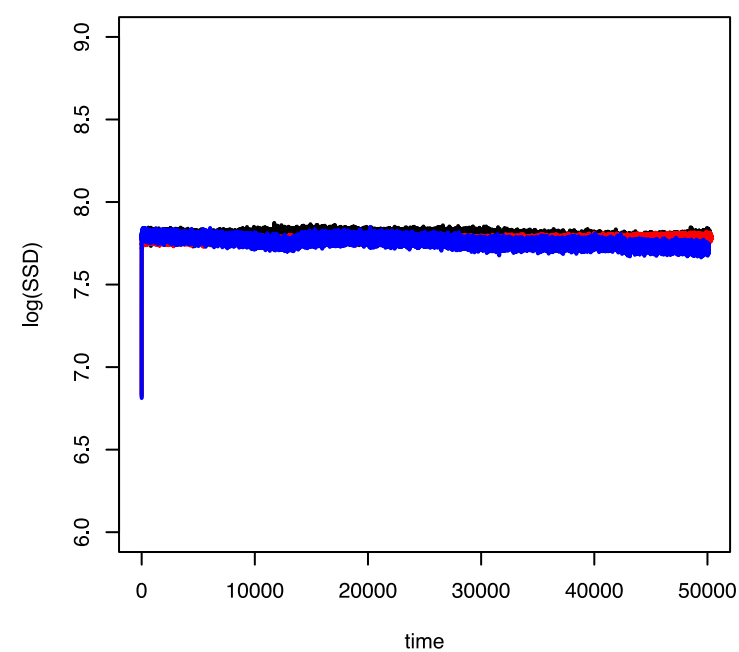

$\Delta=4.4 \times 10^{-4}$

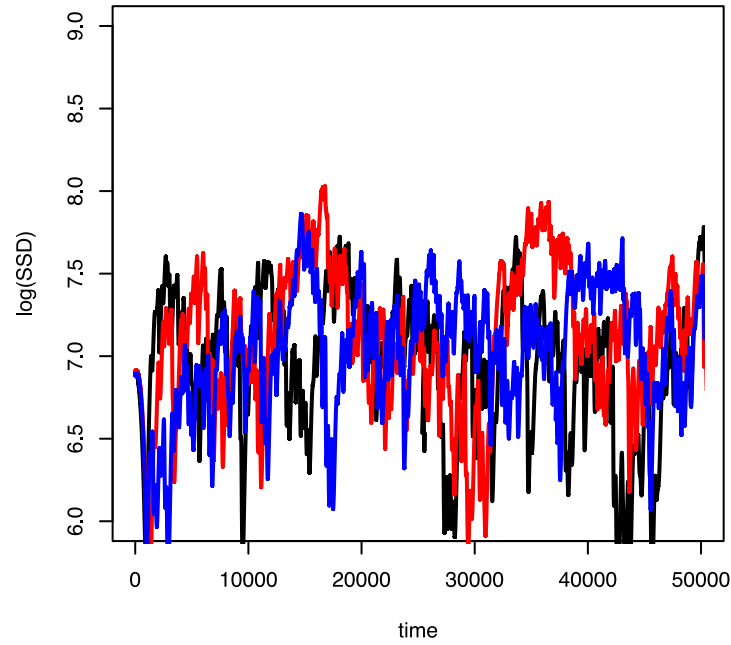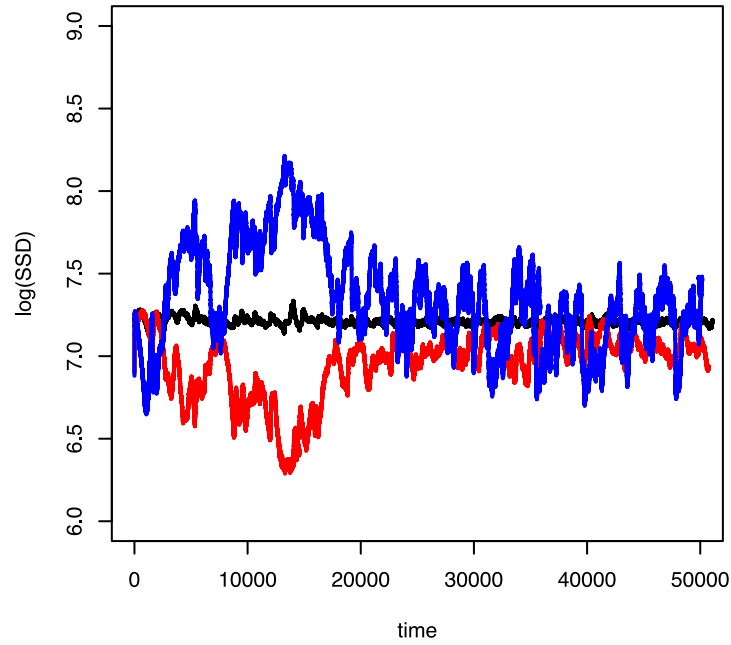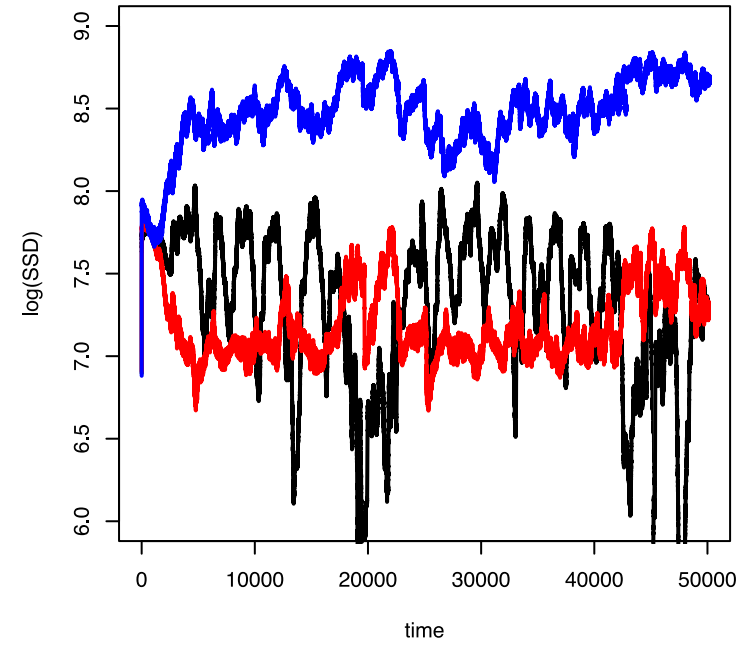

Supplement: Supplementary file 1 [file genes-11-01433-s001.zip › Figure_S26.pdf]

$\Delta=0$   
 $\sigma_\alpha=0.85, d=0$

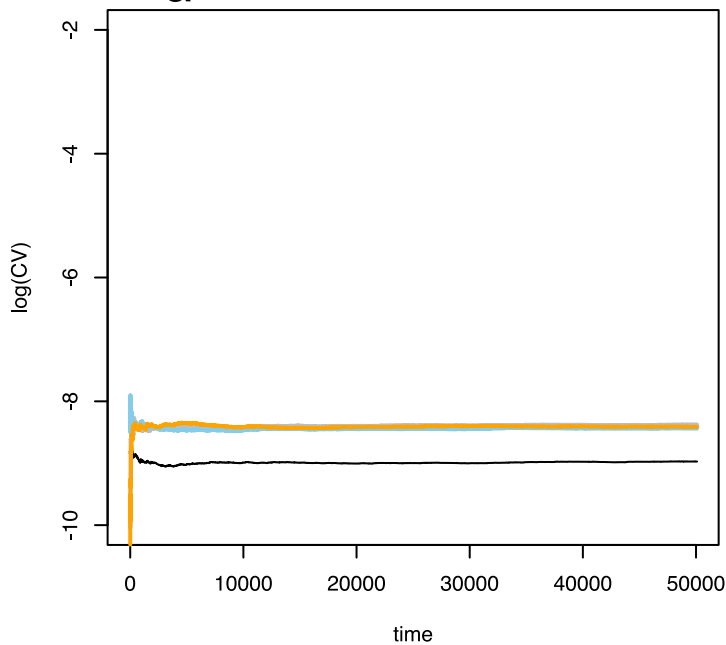

$d=0.01$

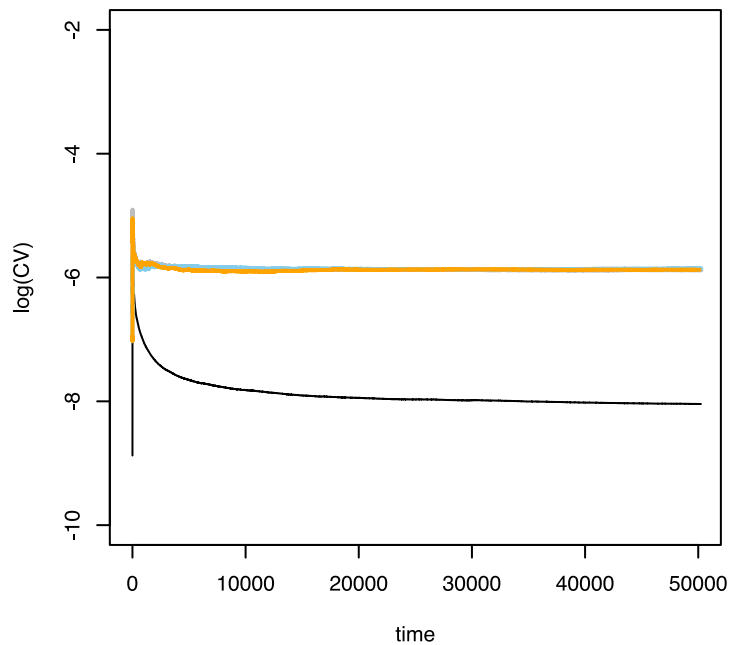

$d=0.1$

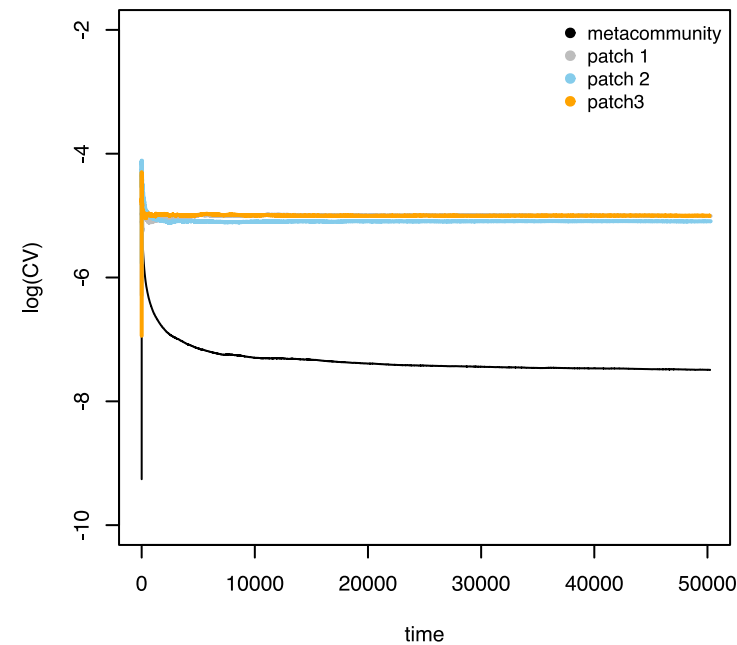

$\Delta=10^{-5}$

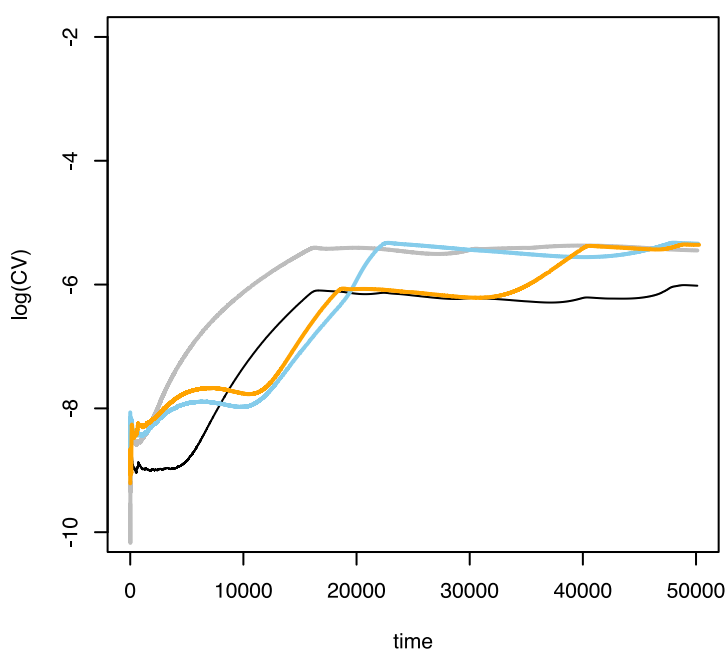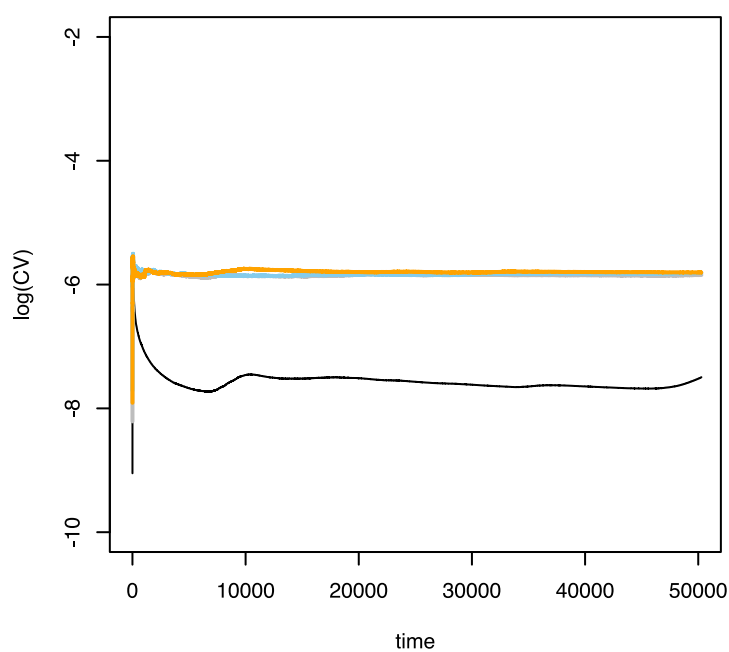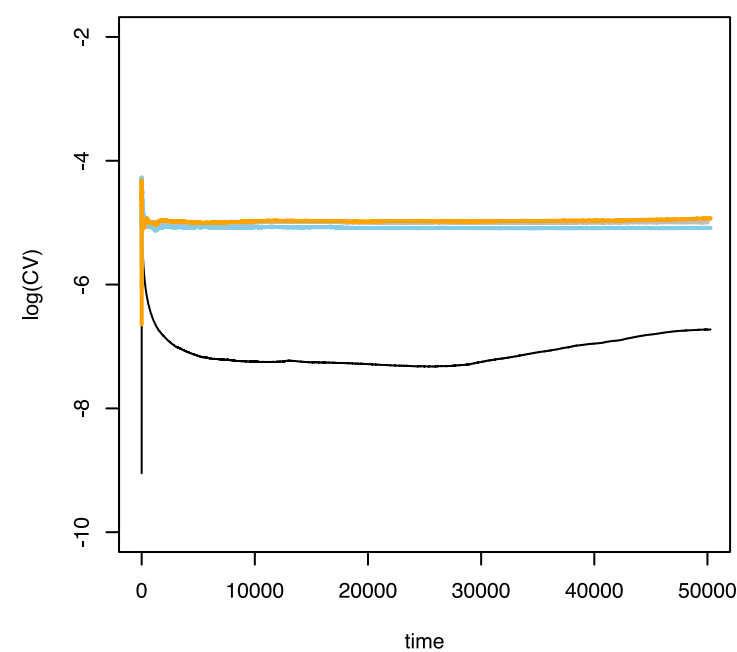

$\Delta=4.4 \times 10^{-4}$

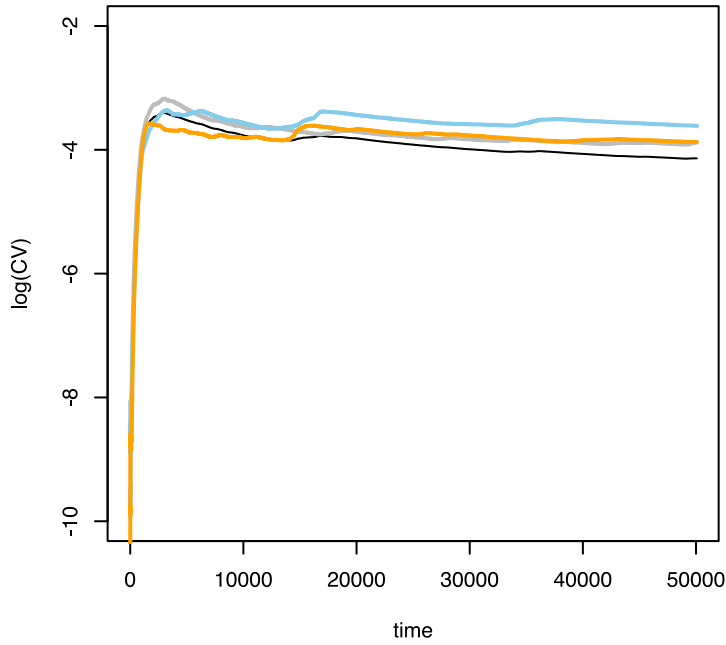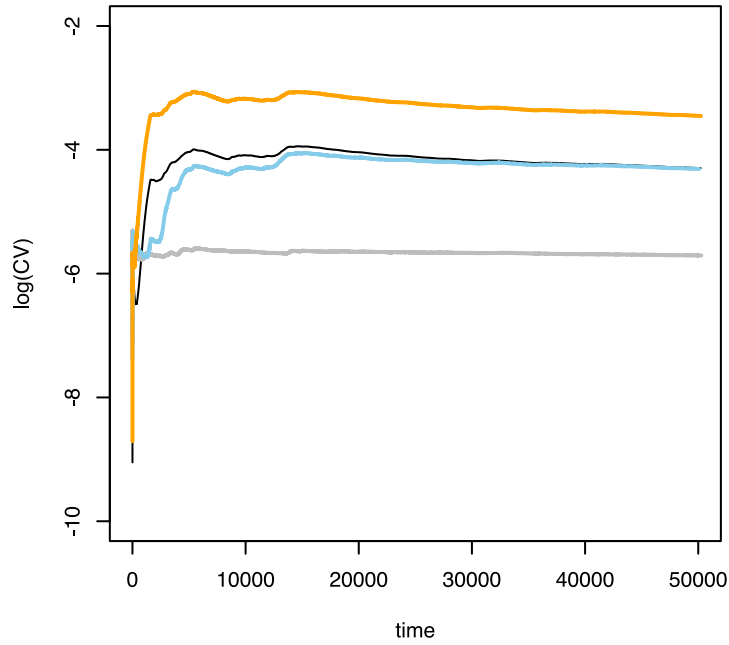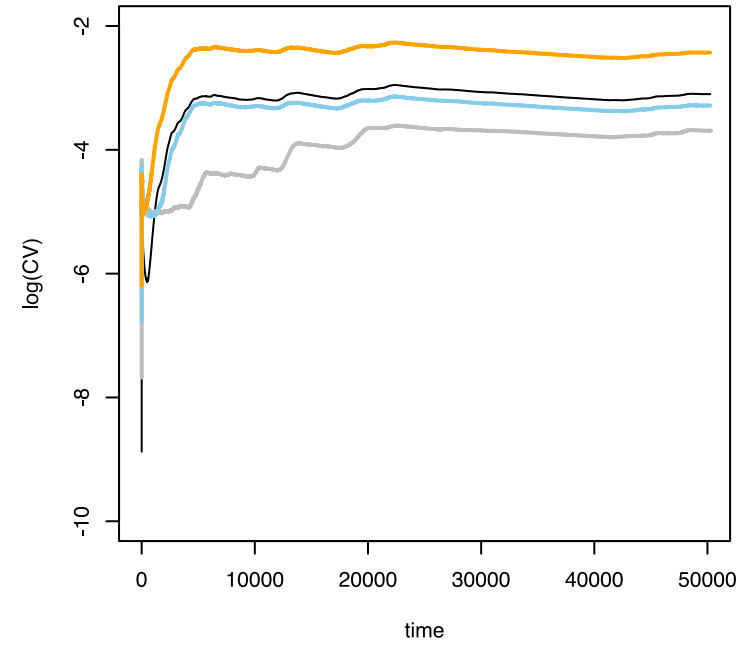

Supplement: Supplementary file 1 [file genes-11-01433-s001.zip › Figure_S27.pdf]

$\Delta=0$   
 $\sigma_\alpha=1.5, d=0$

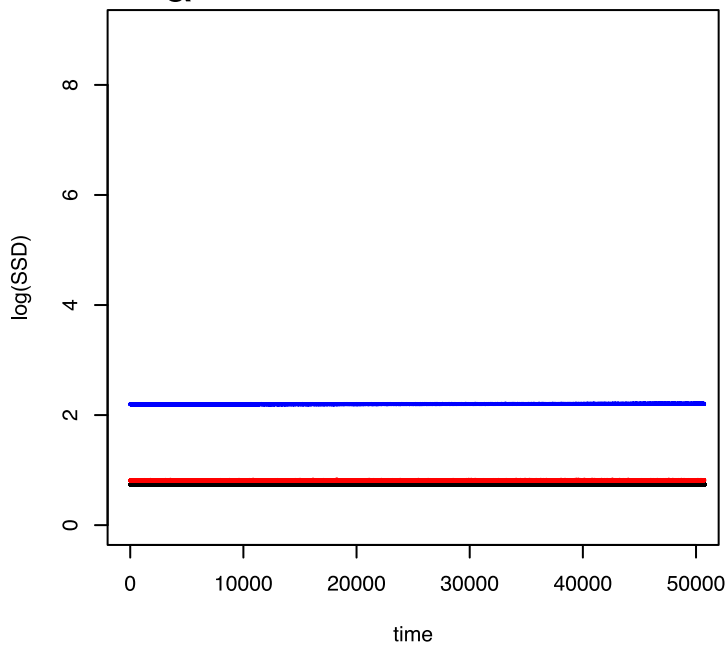

$d=0.01$

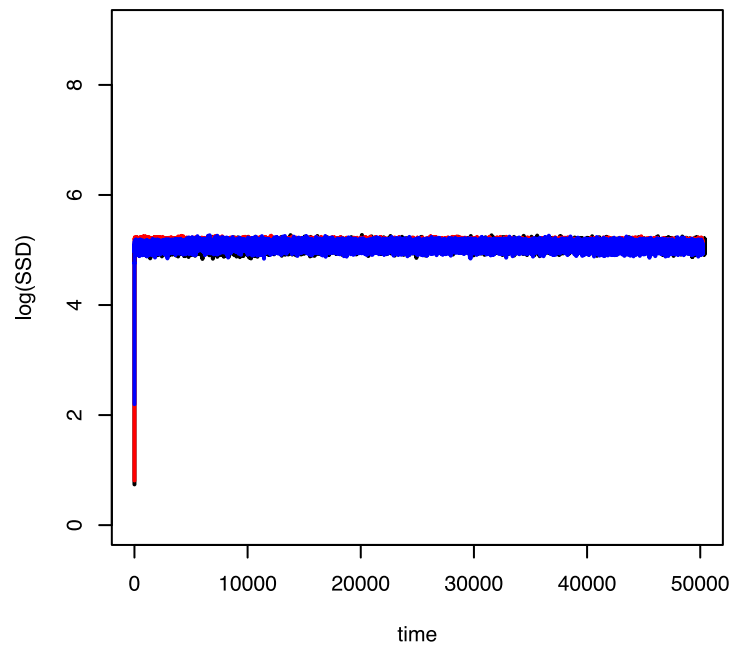

$d=0.1$

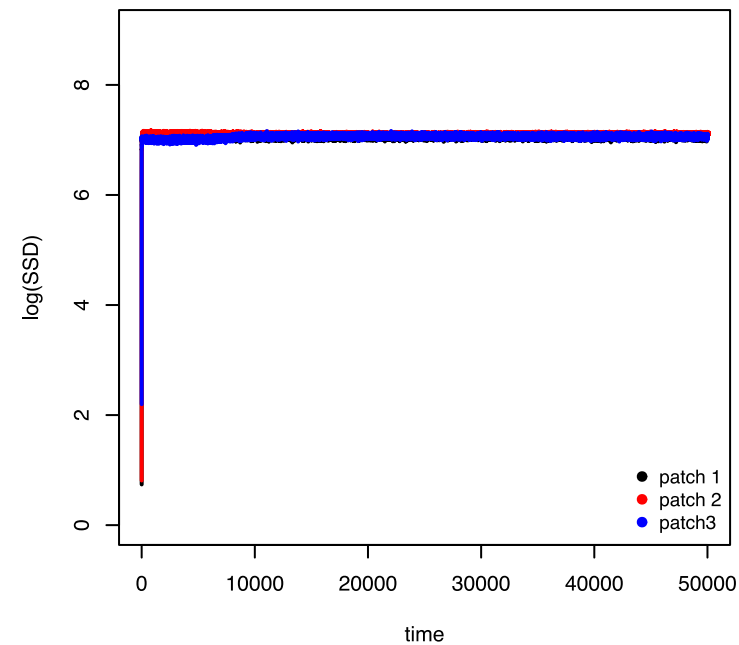

$\Delta=10^{-5}$

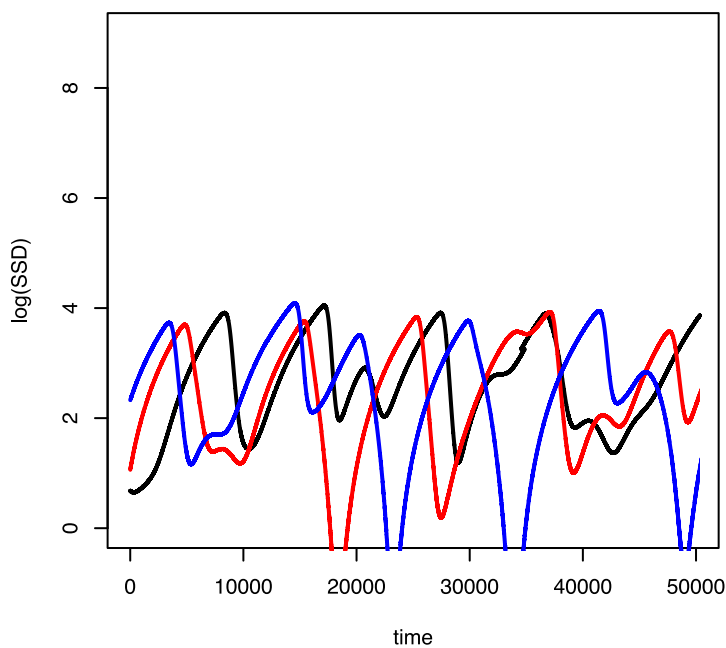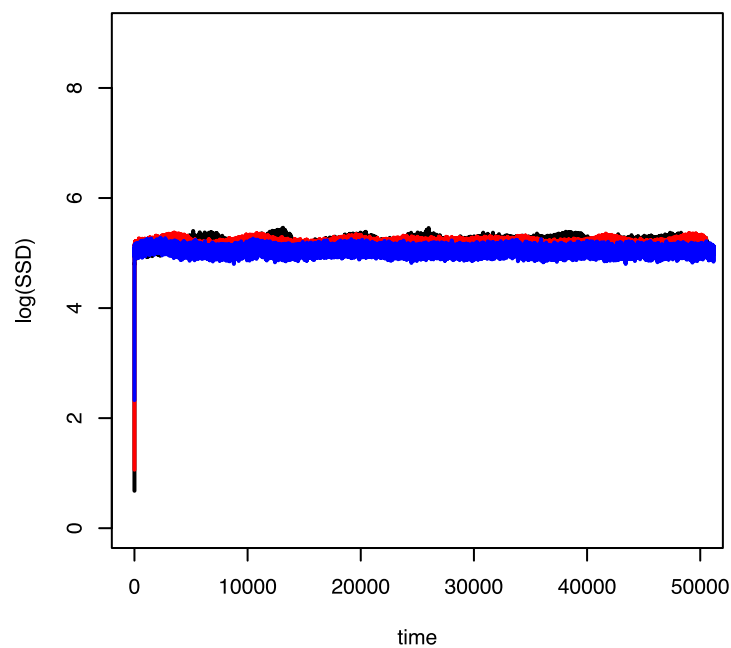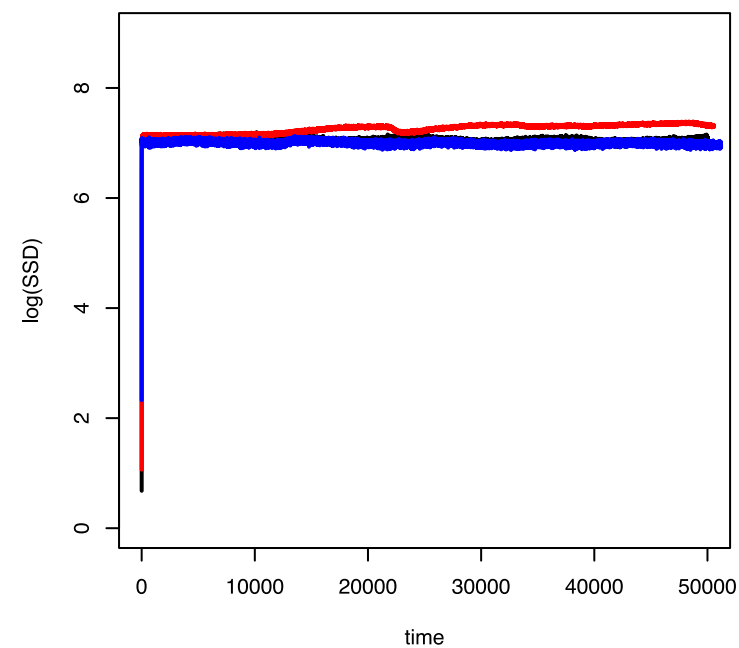

$\Delta=4.4 \times 10^{-4}$

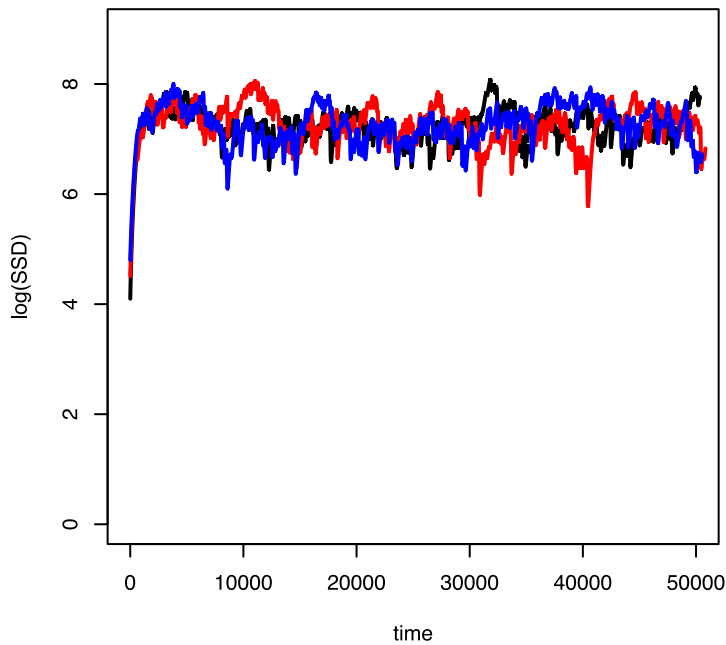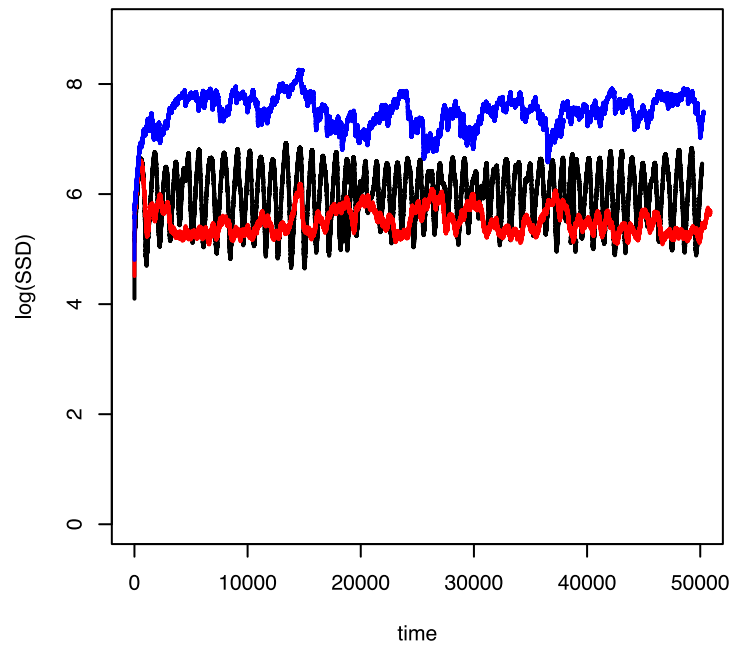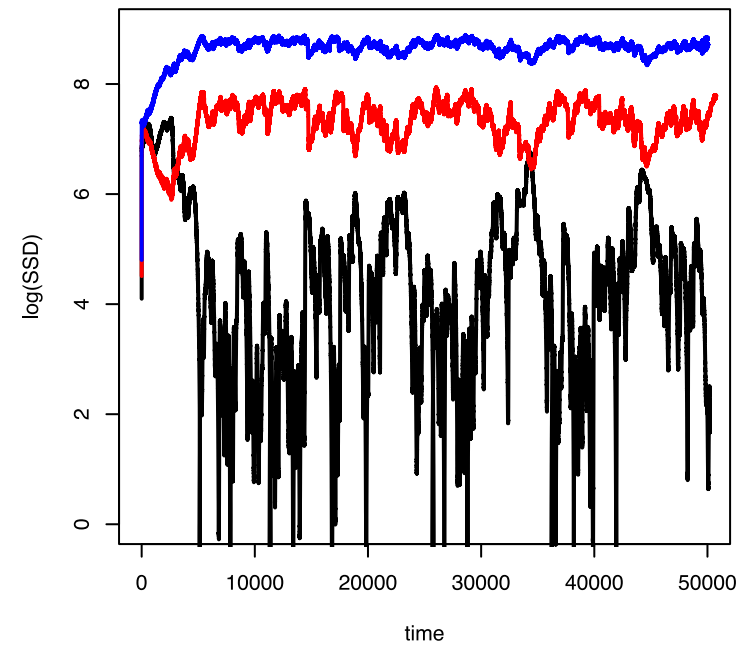

Supplement: Supplementary file 1 [file genes-11-01433-s001.zip › Figure_S28.pdf]

$\Delta=0$   
 $\sigma_\alpha=1.5, d=0$

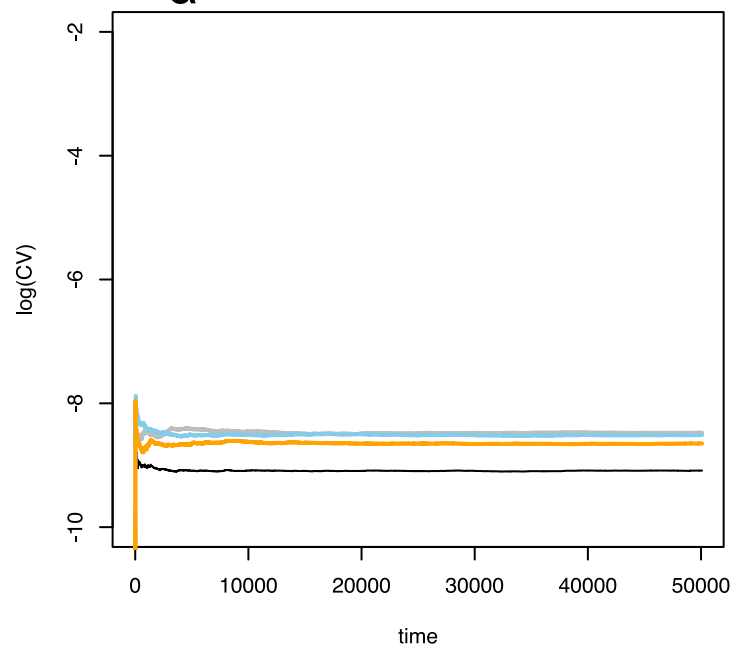

$d=0.01$

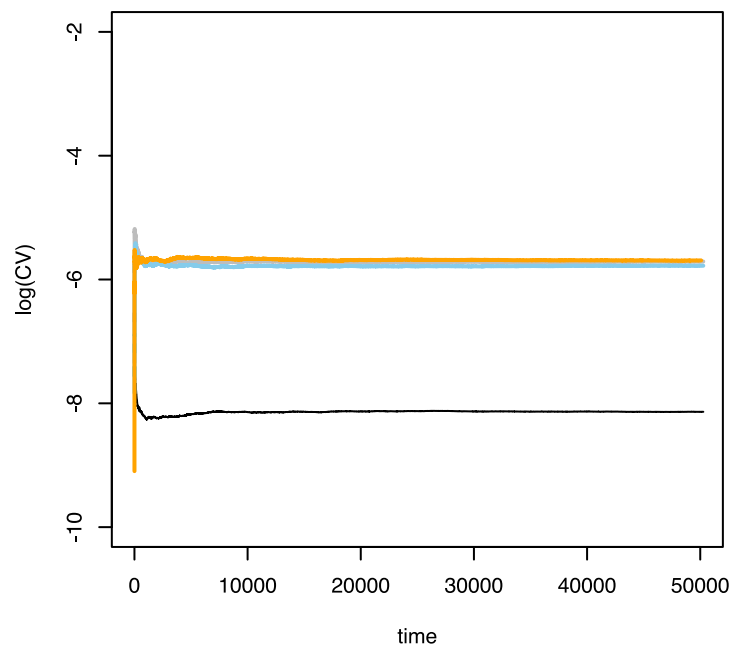

$d=0.1$

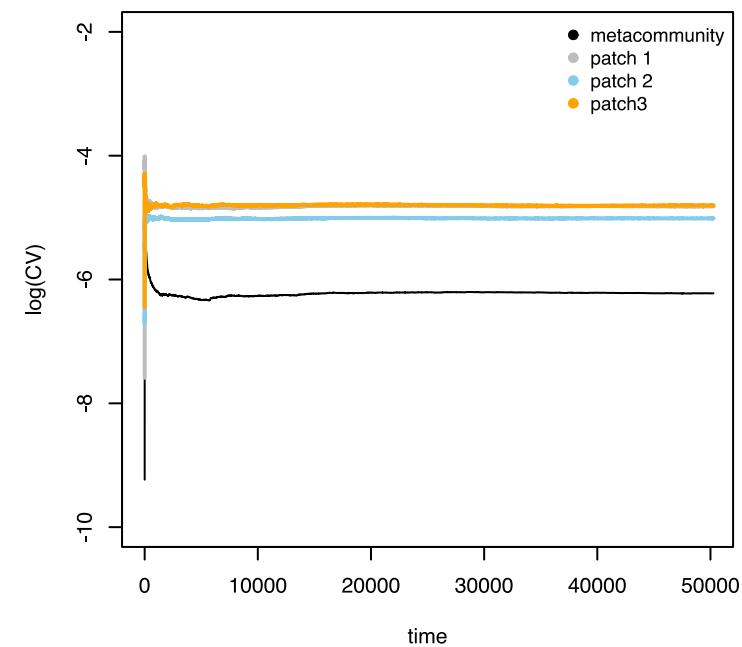

$\Delta=10^{-5}$

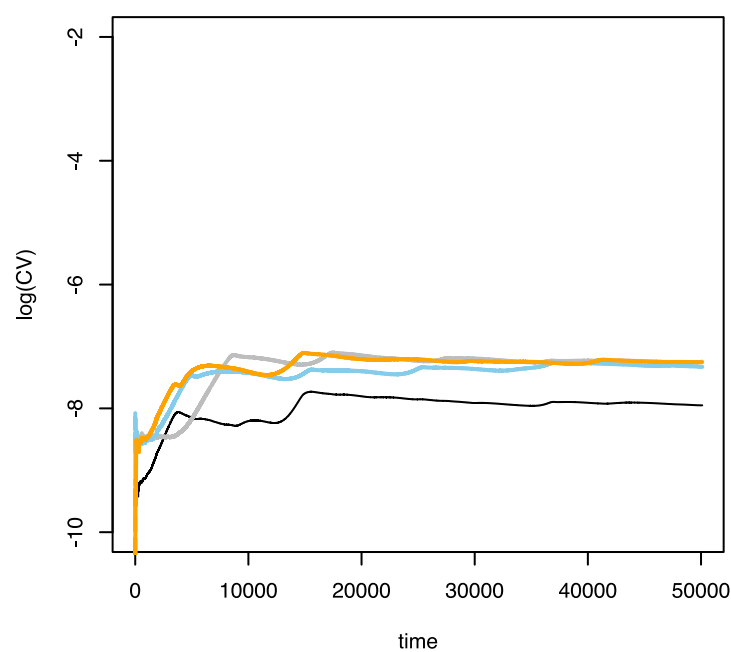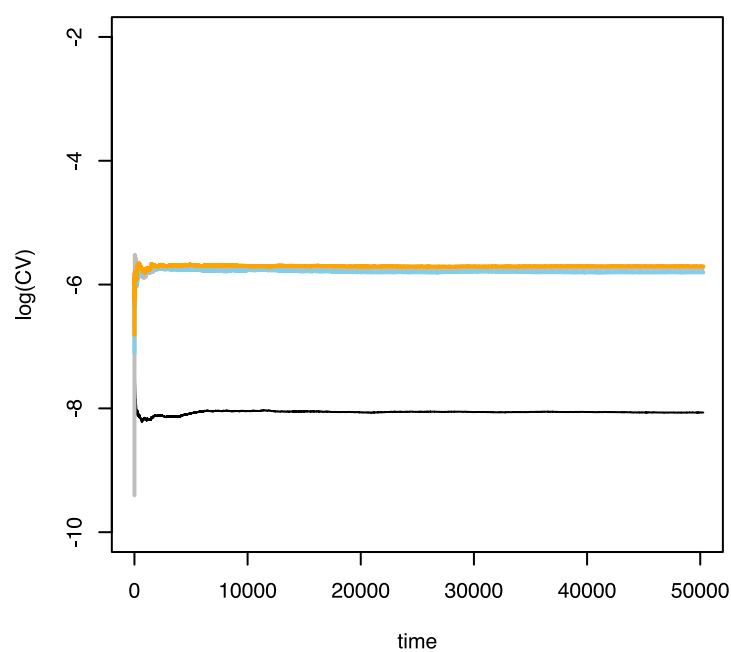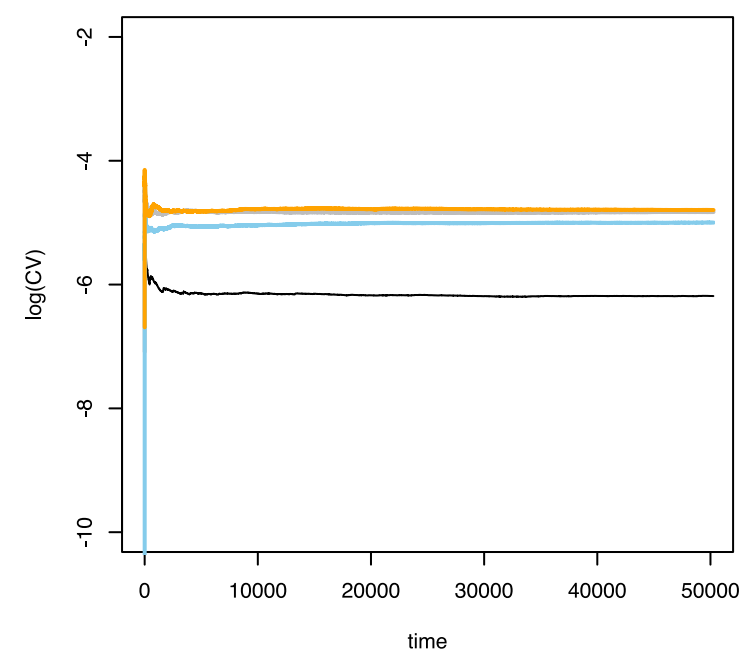

$\Delta=4.4 \times 10^{-4}$

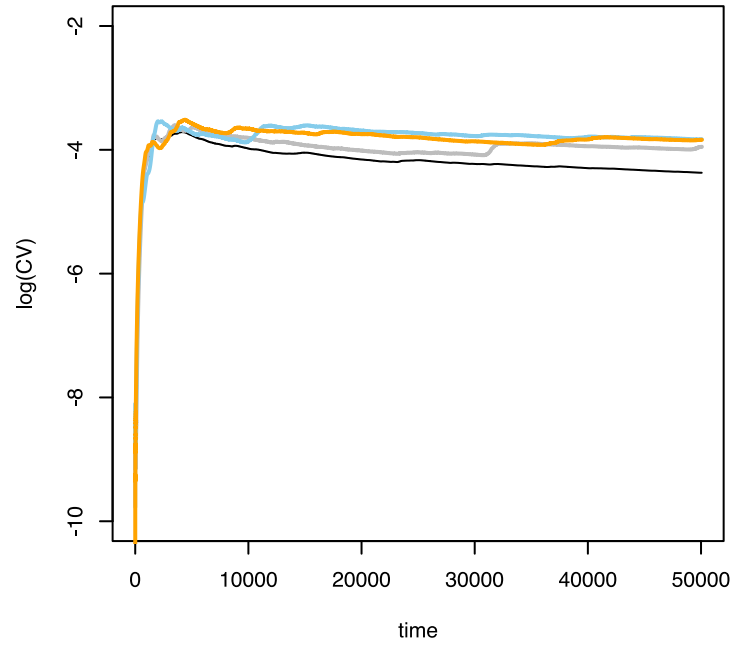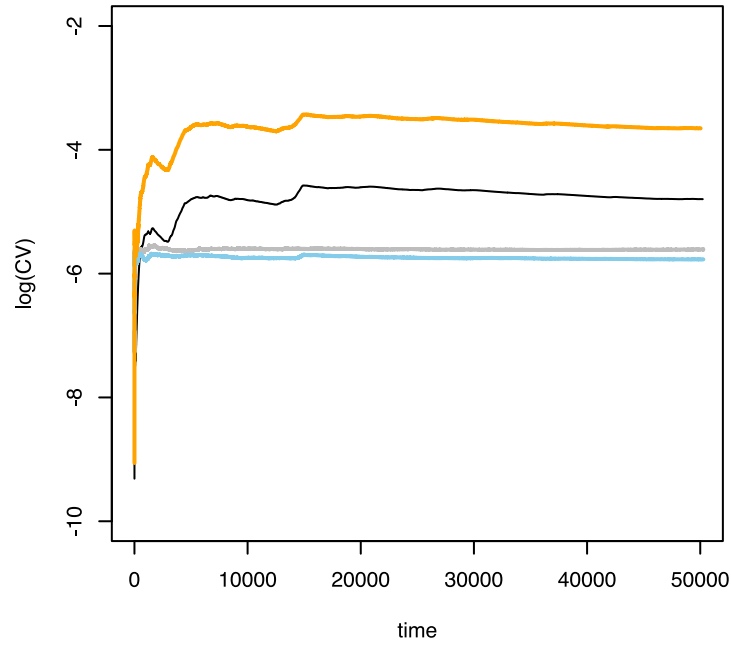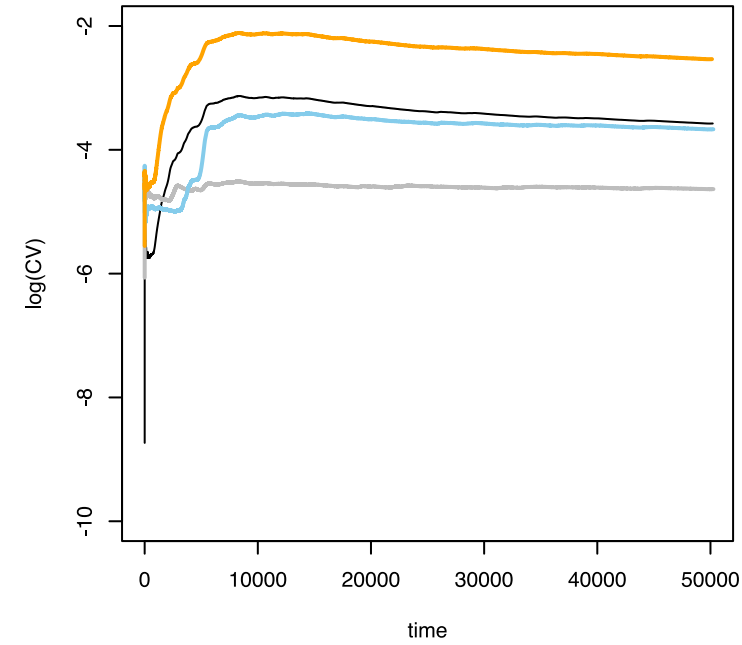

Supplement: Supplementary file 1 [file genes-11-01433-s001.zip › Figure_S29.pdf]

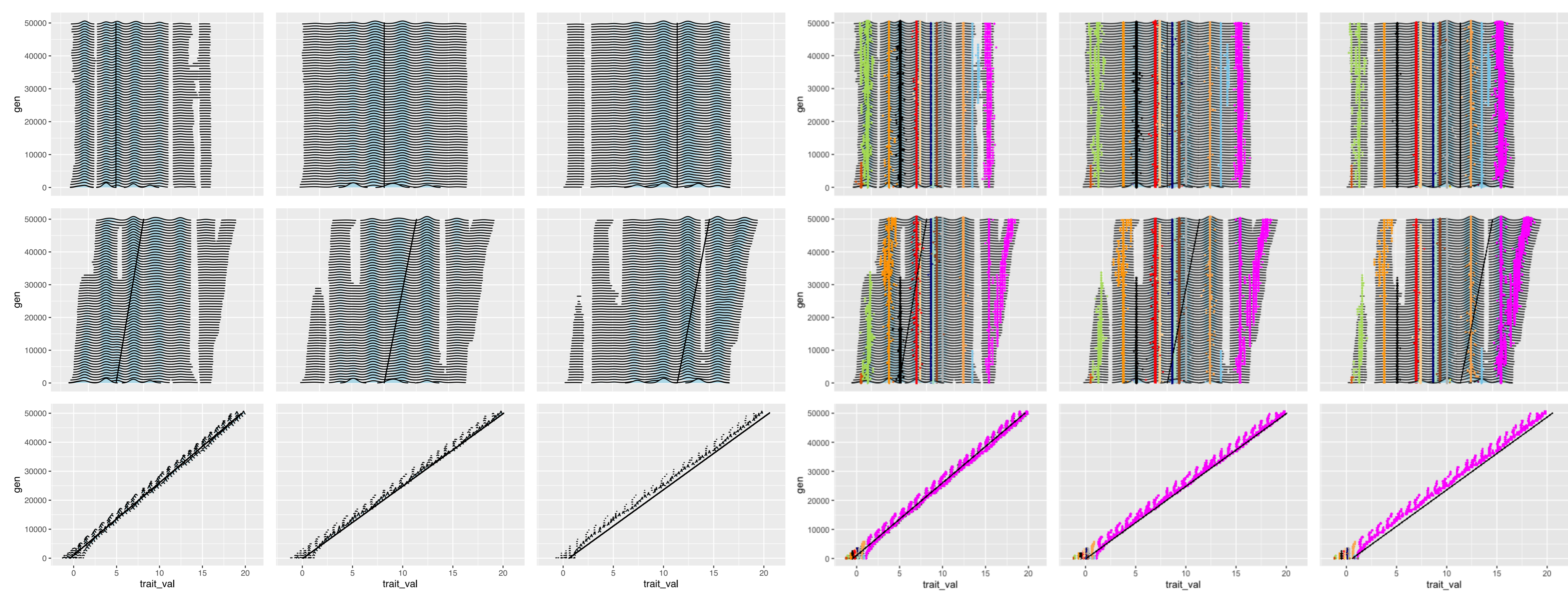

Supplement: Supplementary file 1 [file genes-11-01433-s001.zip › Figure_S3.pdf]

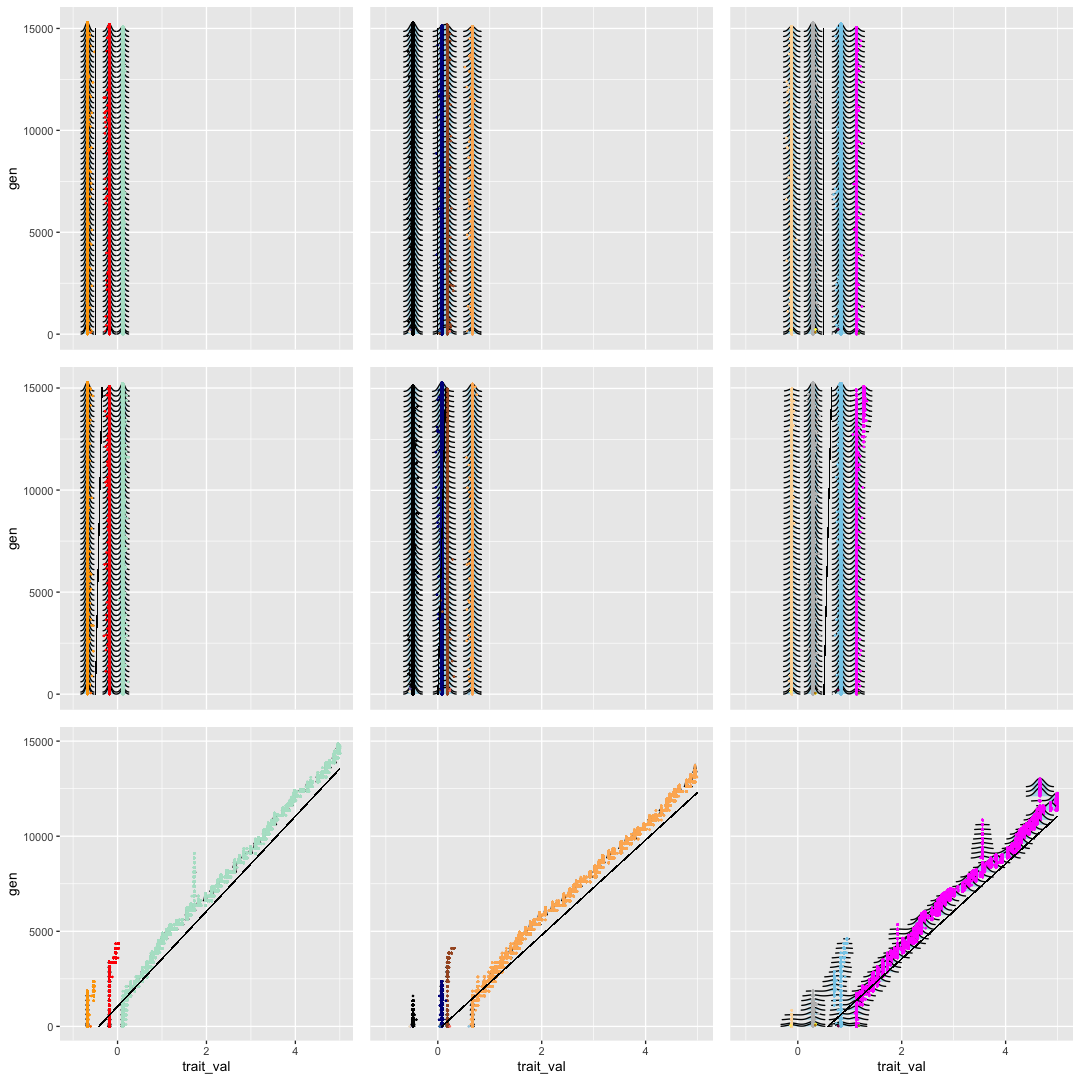

Supplement: Supplementary file 1 [file genes-11-01433-s001.zip › Figure_S4.png]

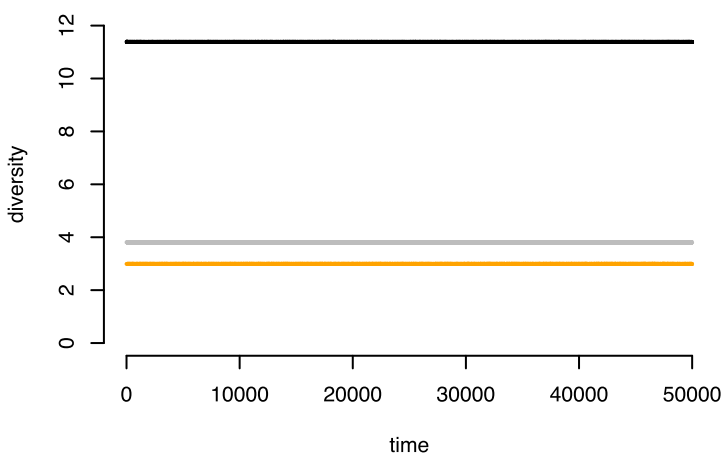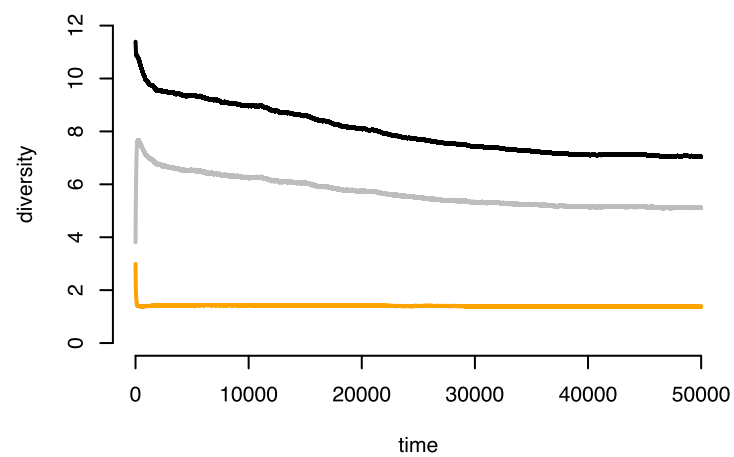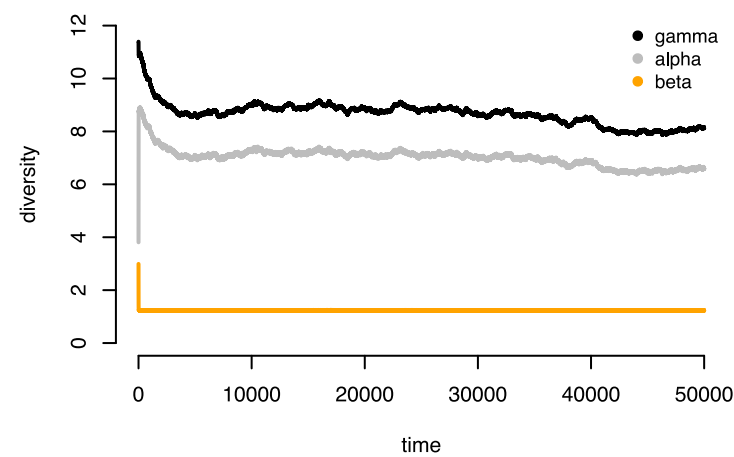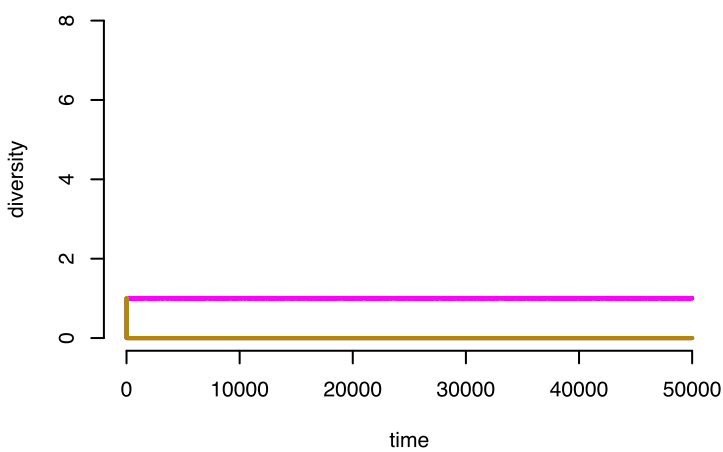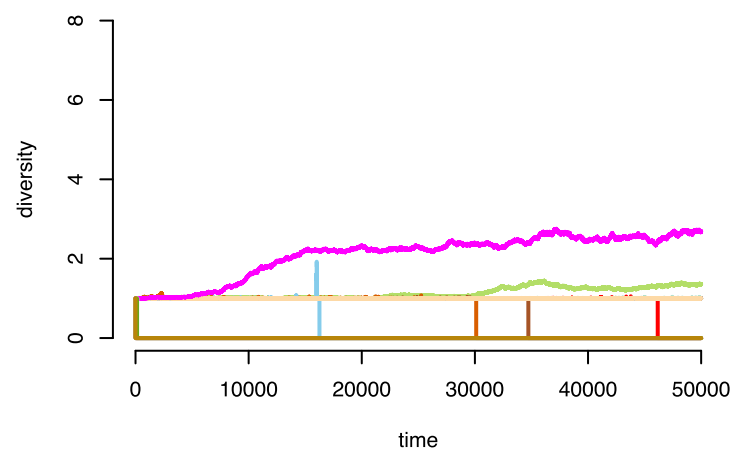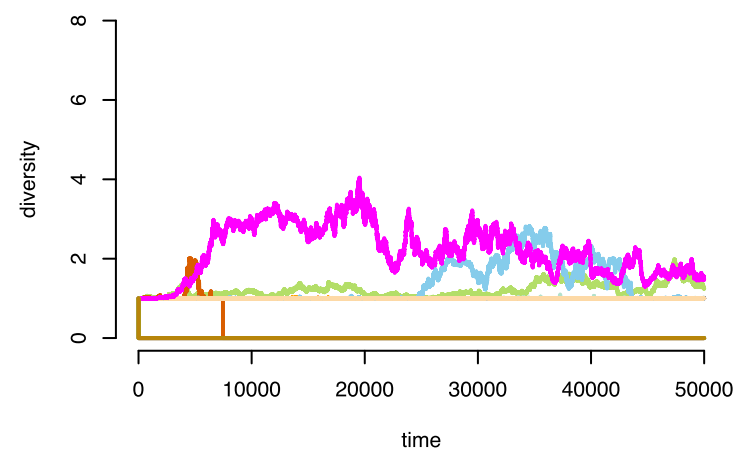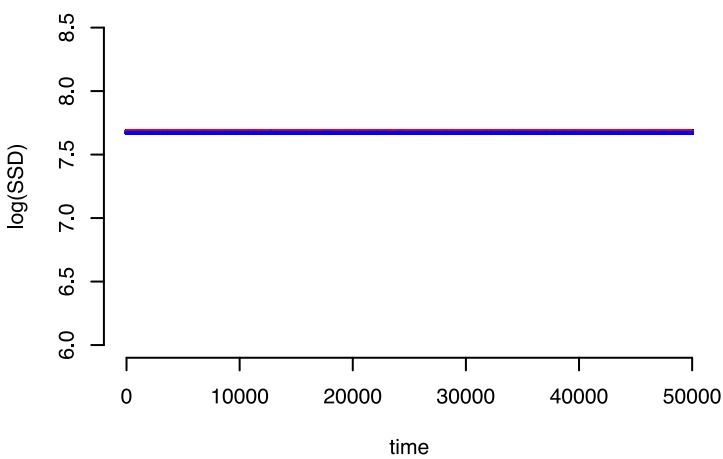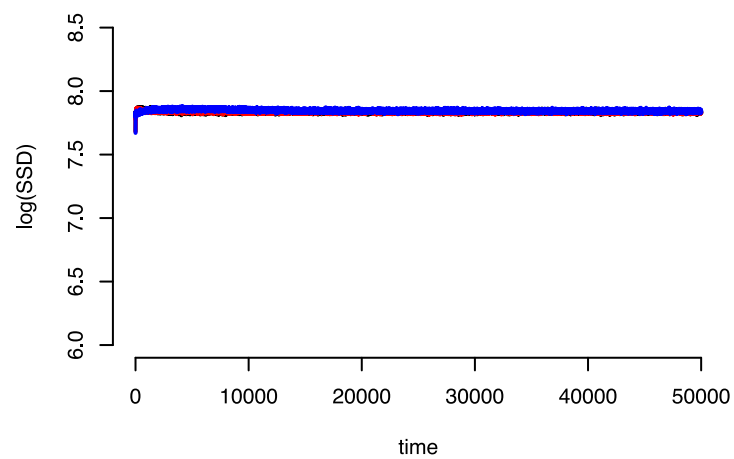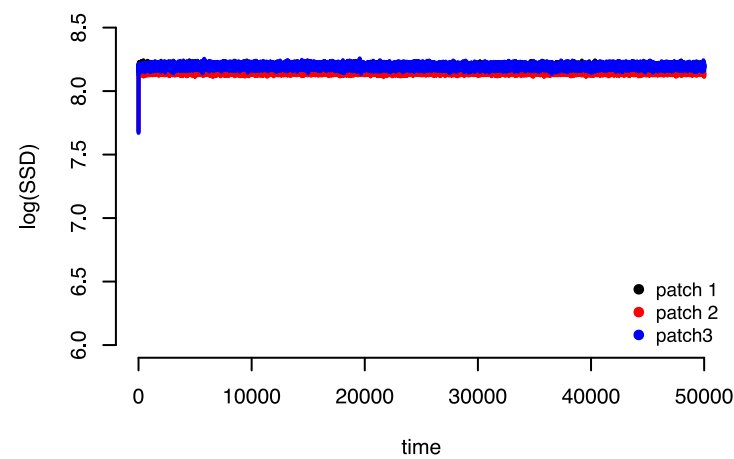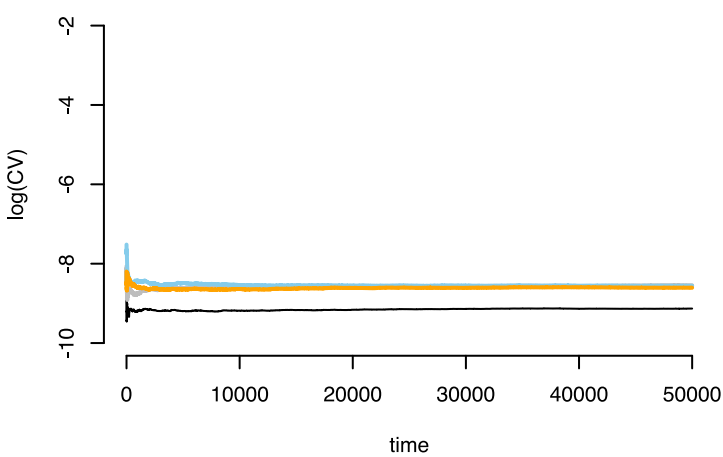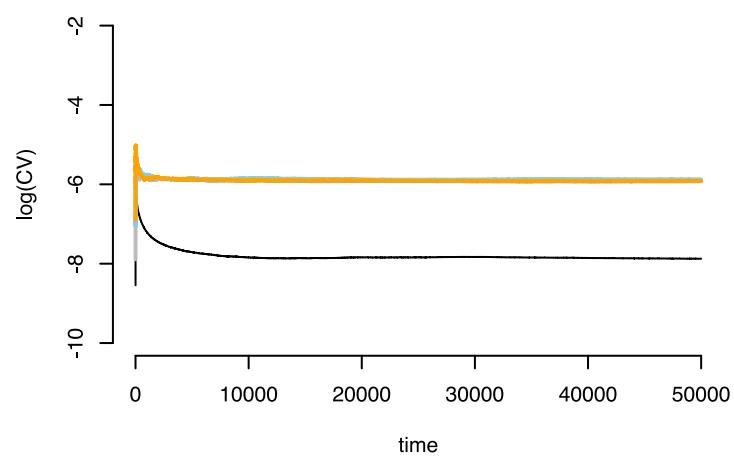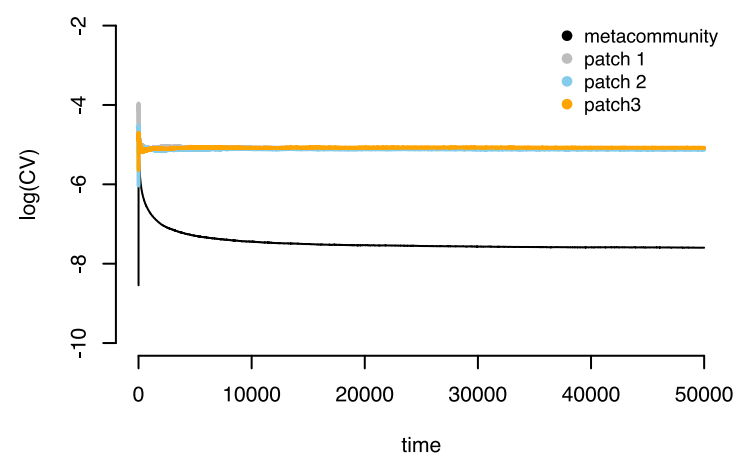

Supplement: Supplementary file 1 [file genes-11-01433-s001.zip › Figure_S5.pdf]

a)

Patch 1

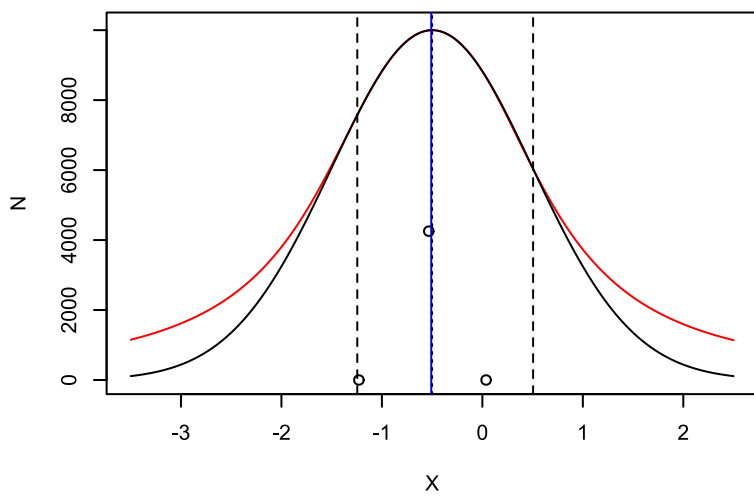

Patch 2

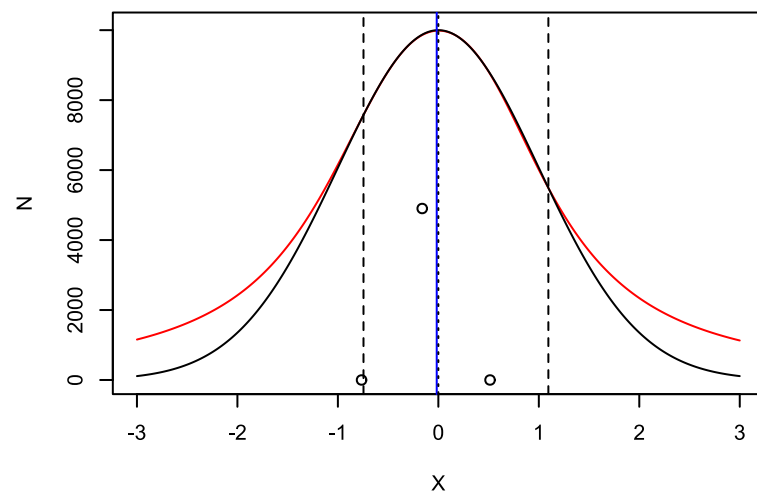

Patch 3

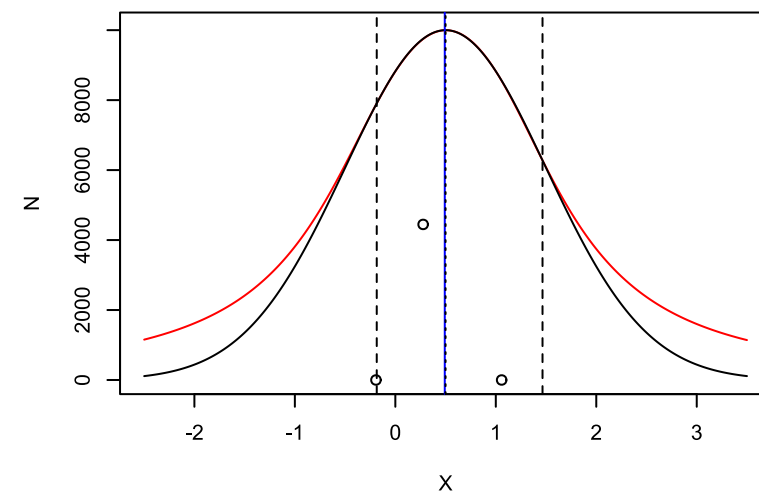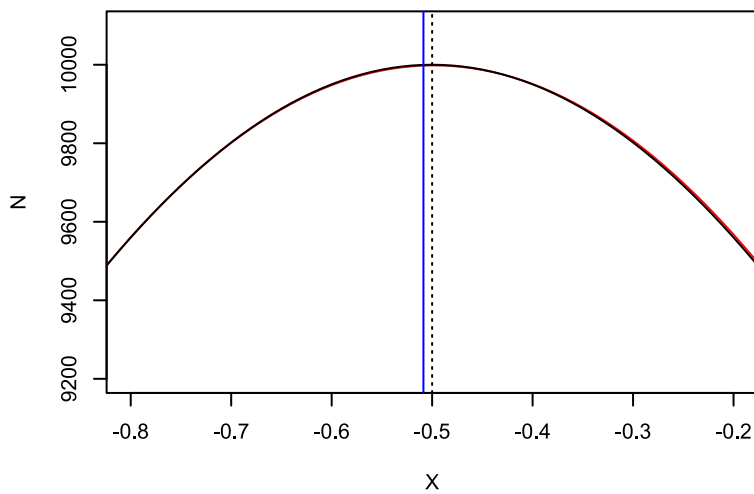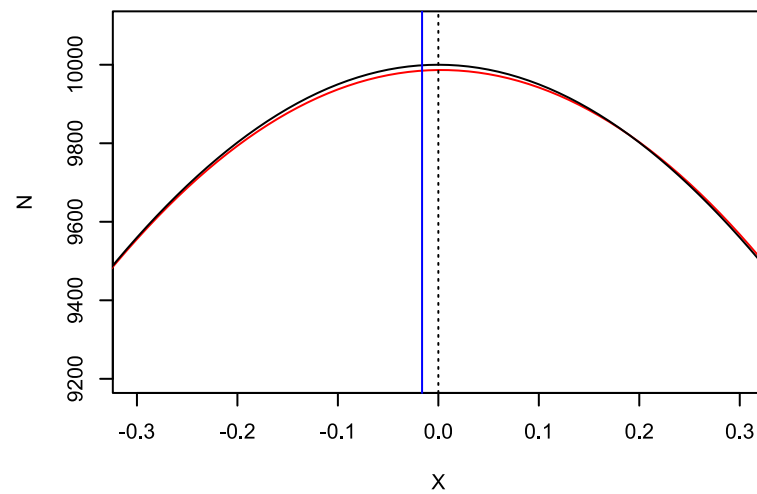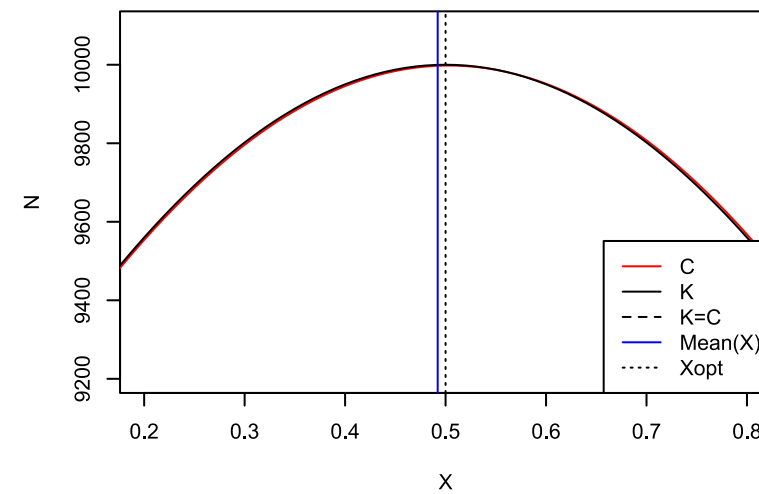

b)

Patch 1

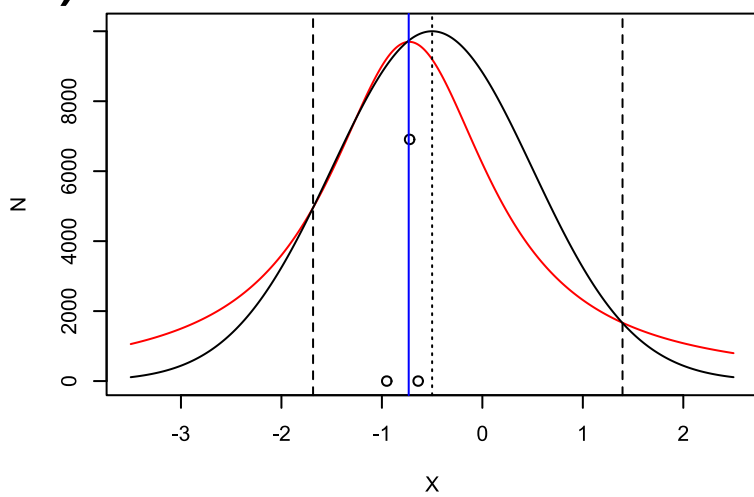

Patch 2

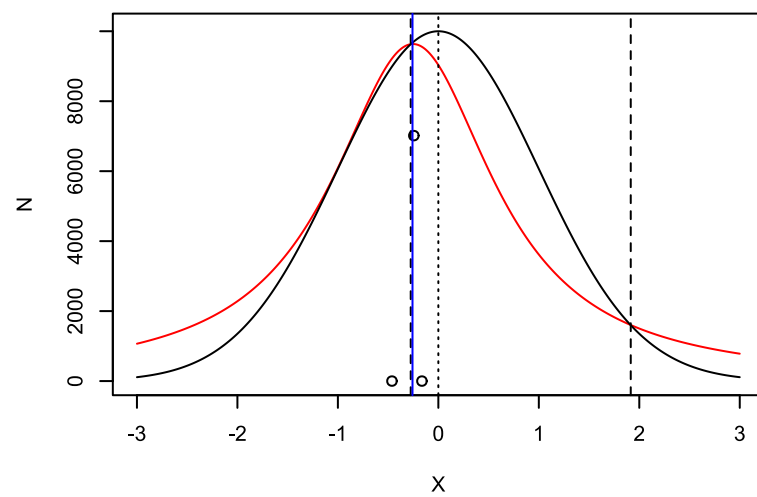

Patch 3

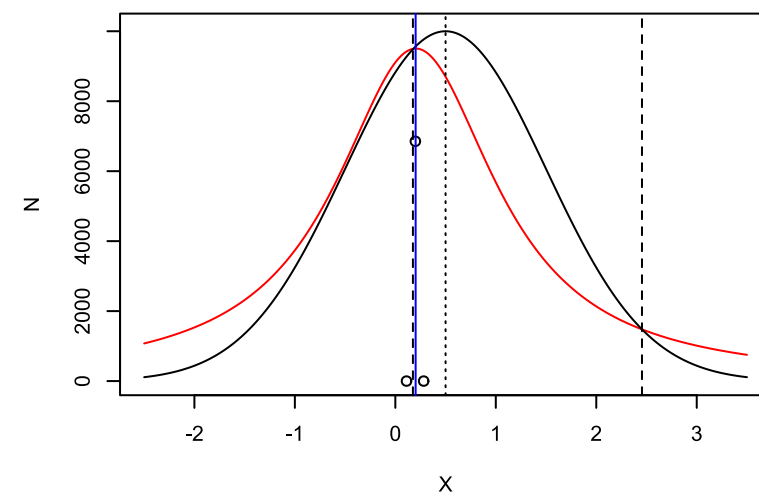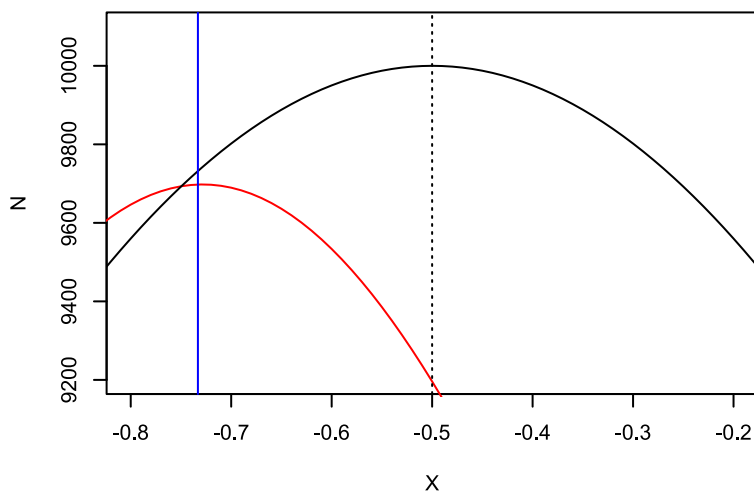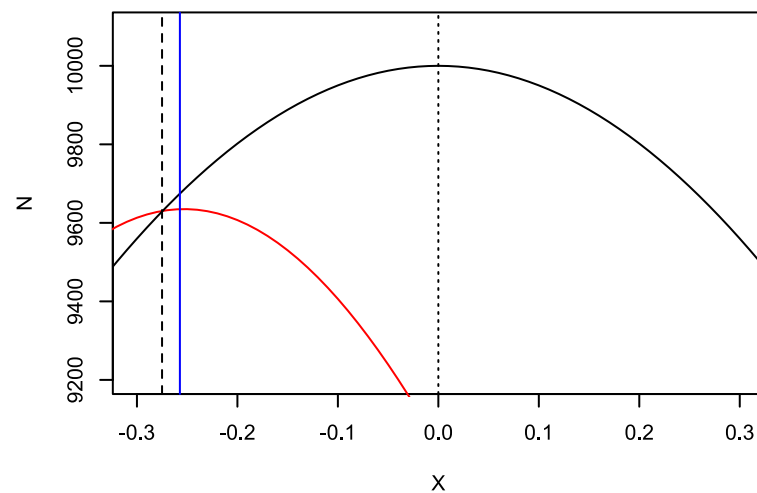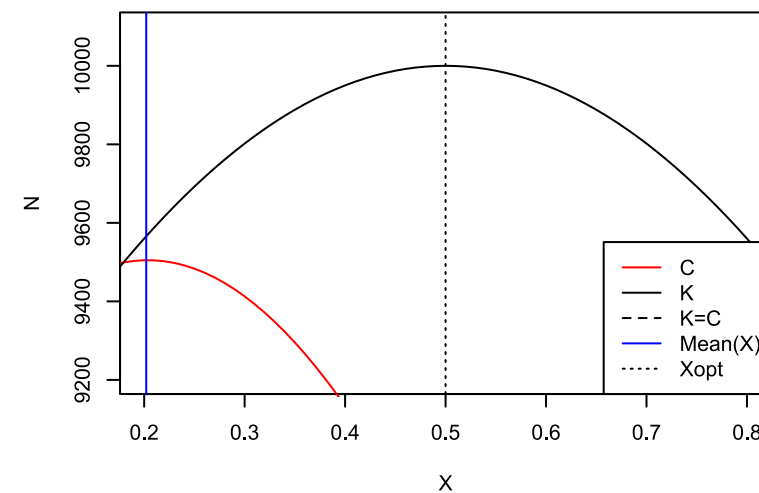

Supplement: Supplementary file 1 [file genes-11-01433-s001.zip › Figure_S6.pdf]

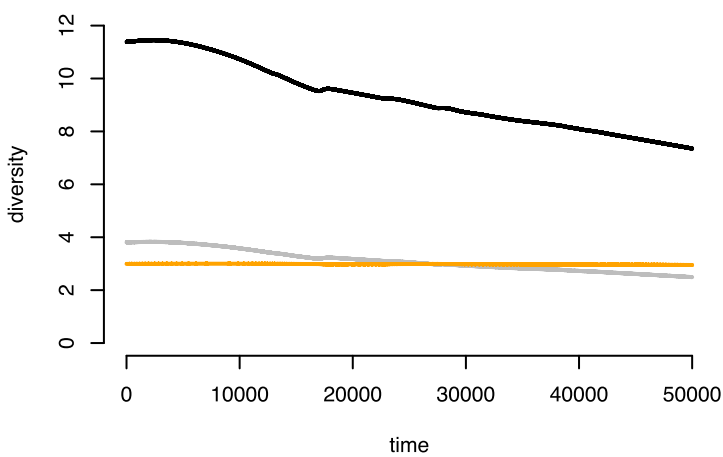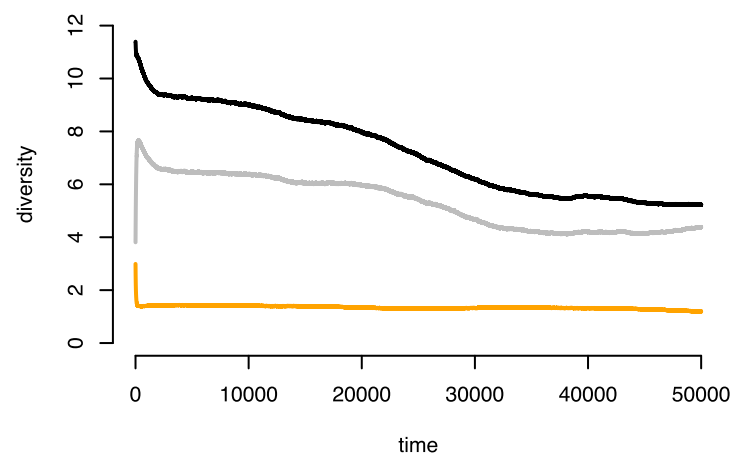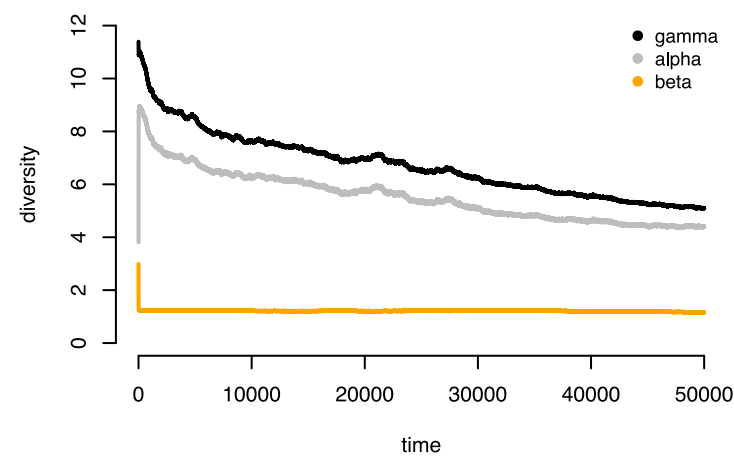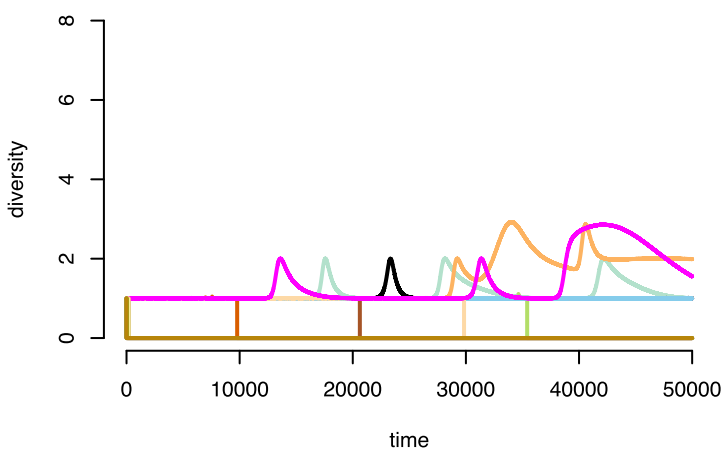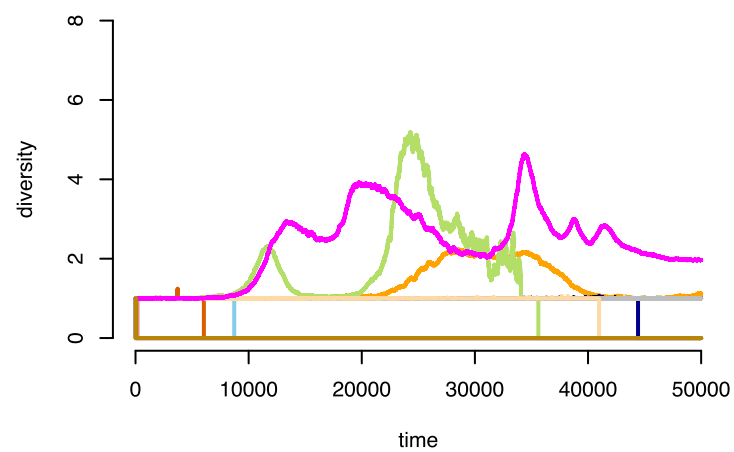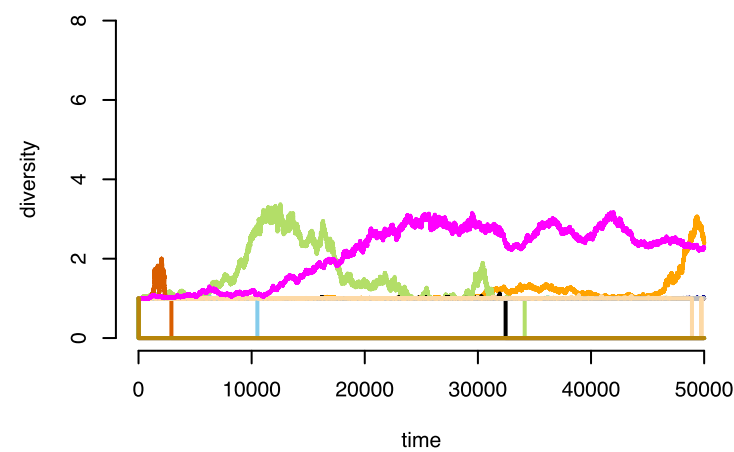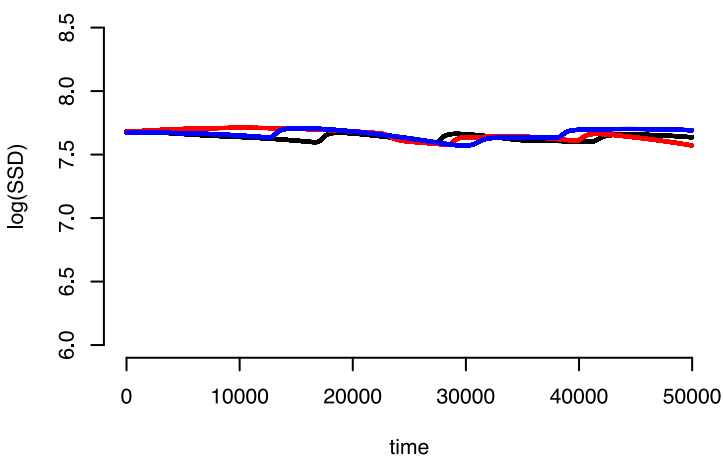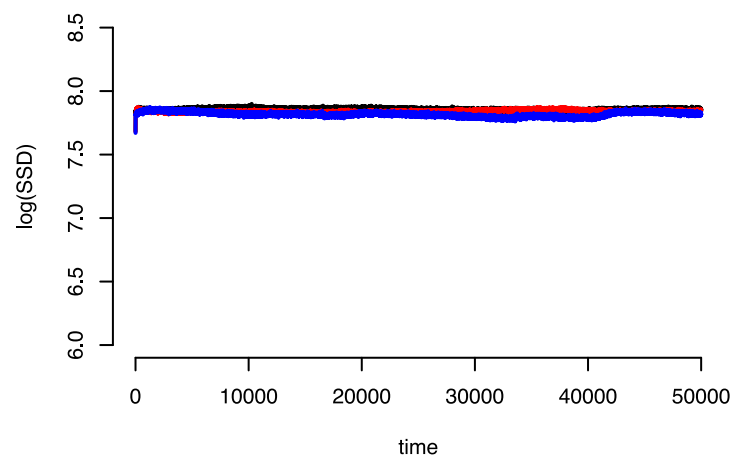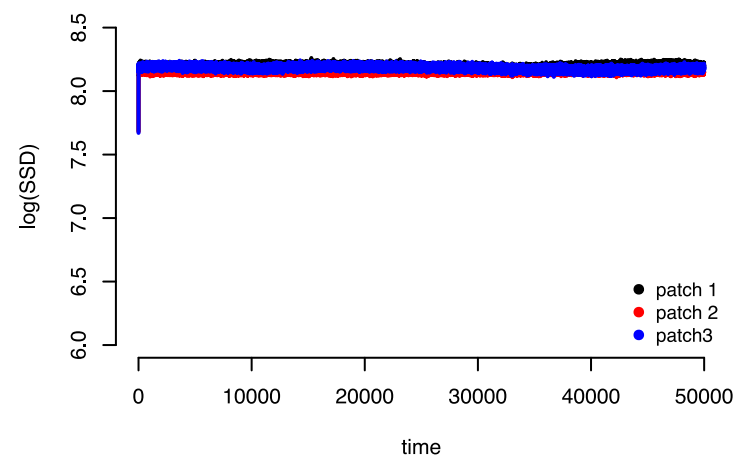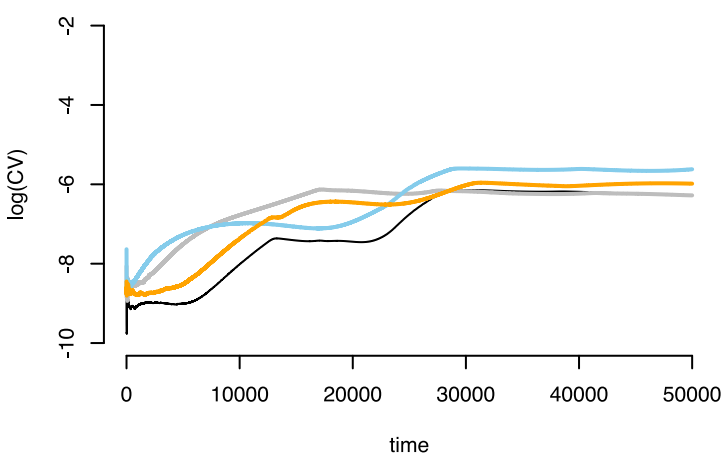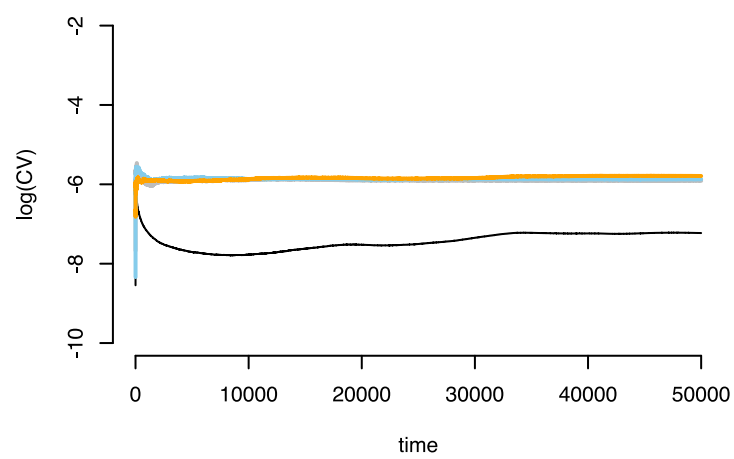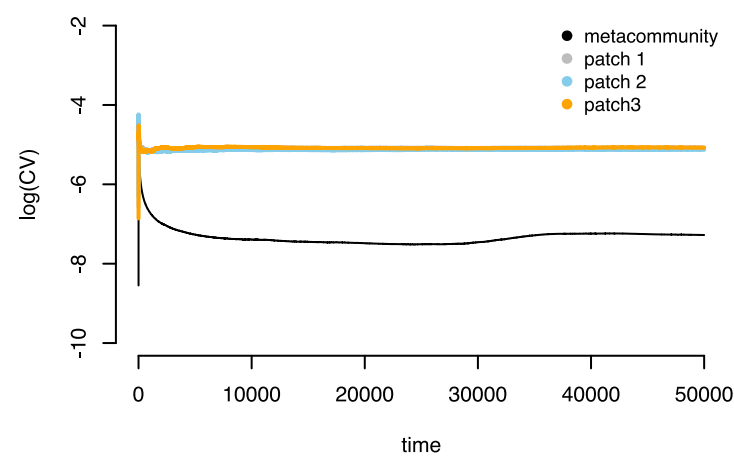

Supplement: Supplementary file 1 [file genes-11-01433-s001.zip › Figure_S7.pdf]

$\Delta=0$   
 $\sigma_\alpha=0.68, d=0$

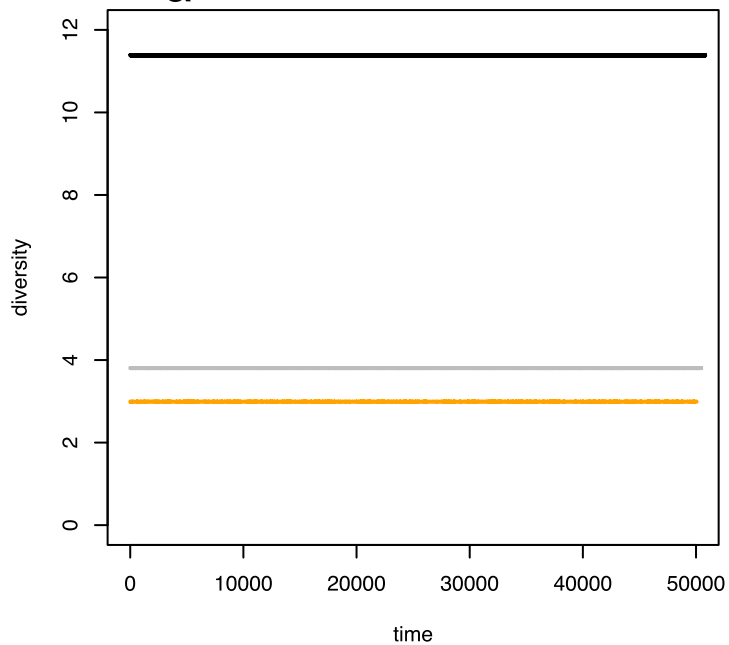

$d=0.01$

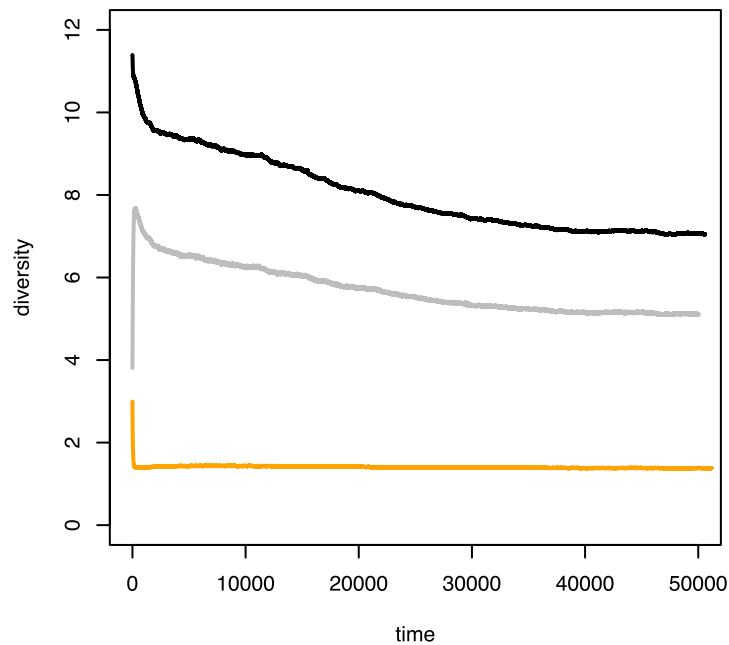

$d=0.1$

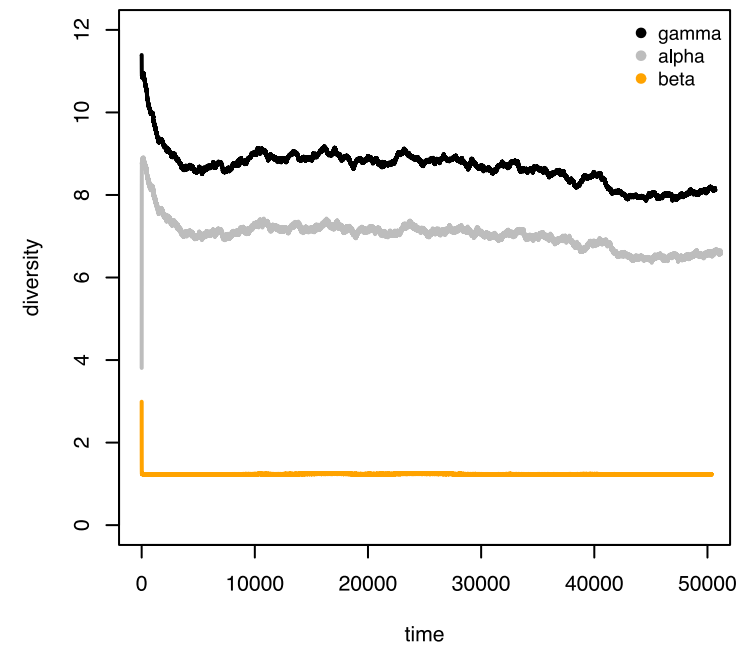

$\Delta=10^{-5}$

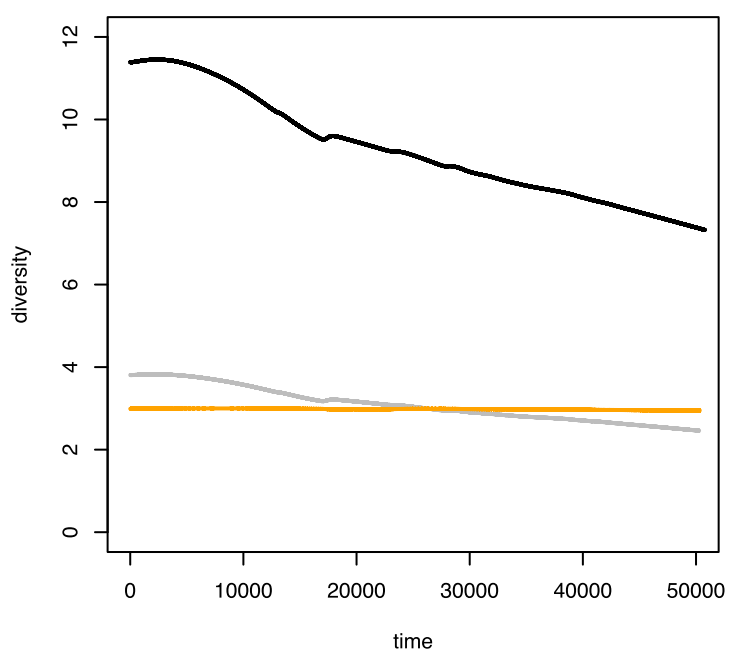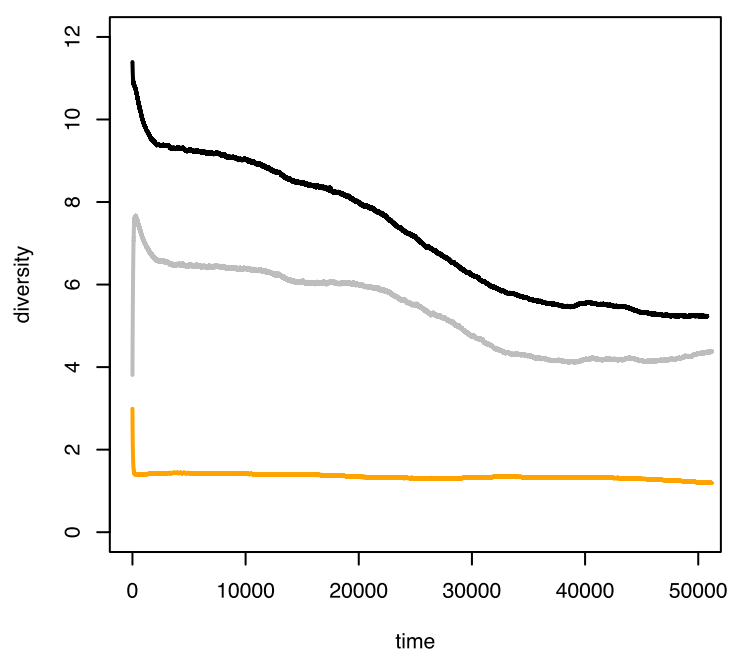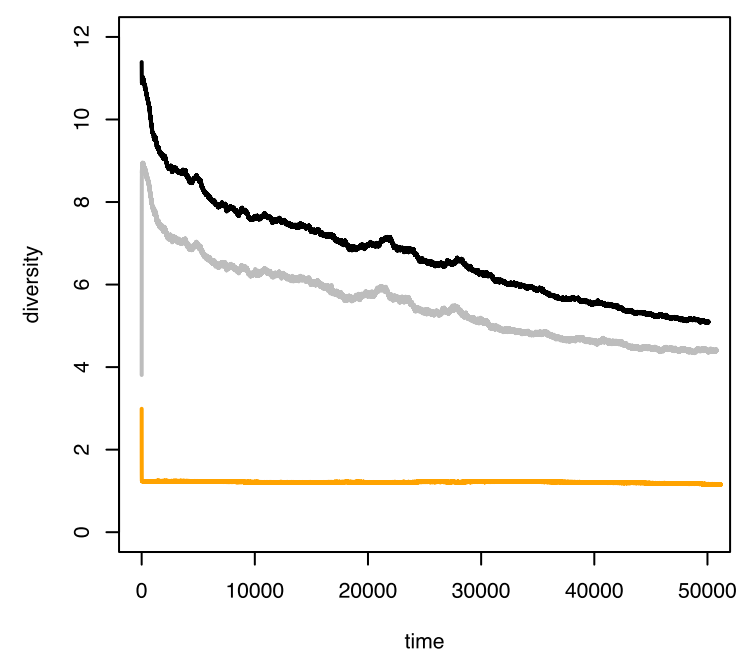

$\Delta=4.4 \times 10^{-4}$

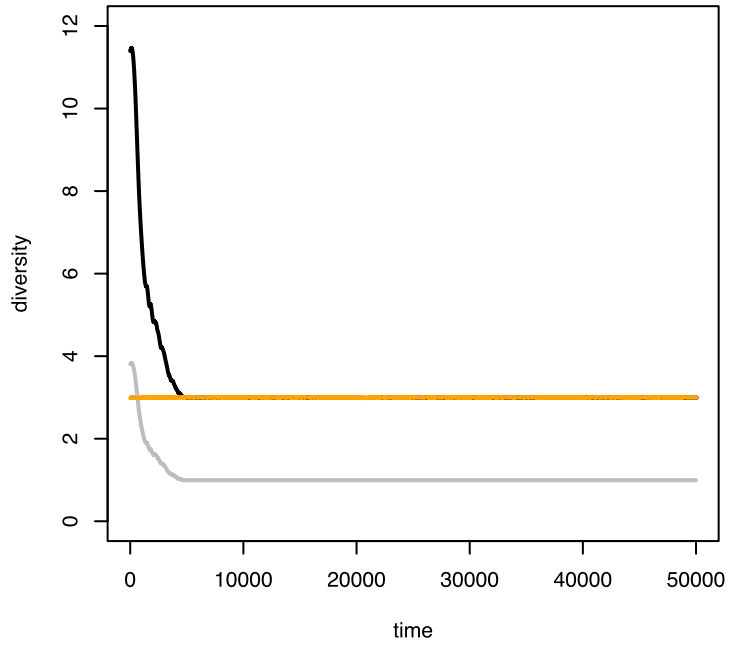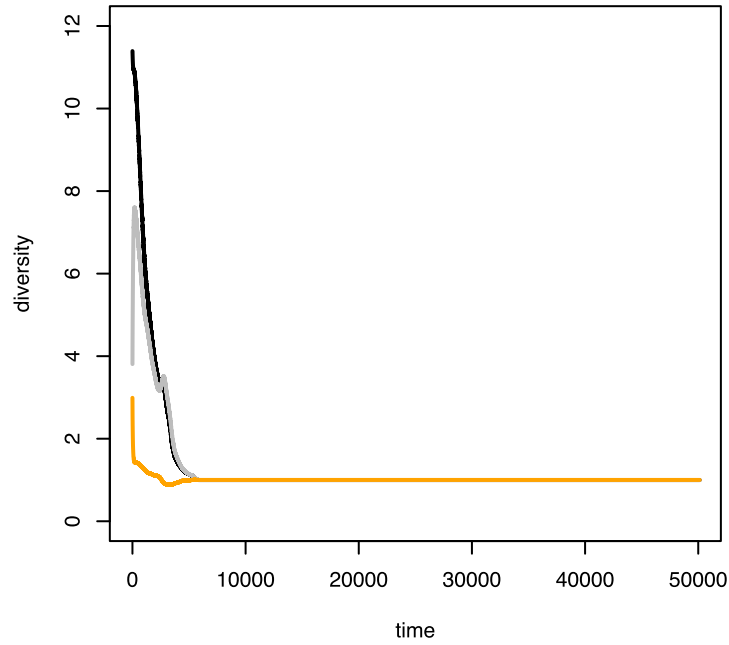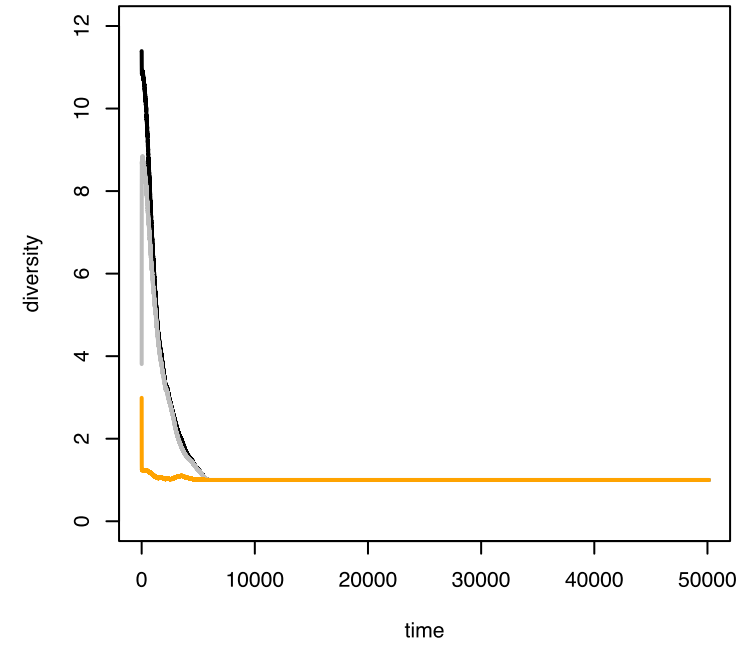

Supplement: Supplementary file 1 [file genes-11-01433-s001.zip › Figure_S8.pdf]

$$\Delta=0$$

$$\sigma_{\alpha}=0.68, d=0$$
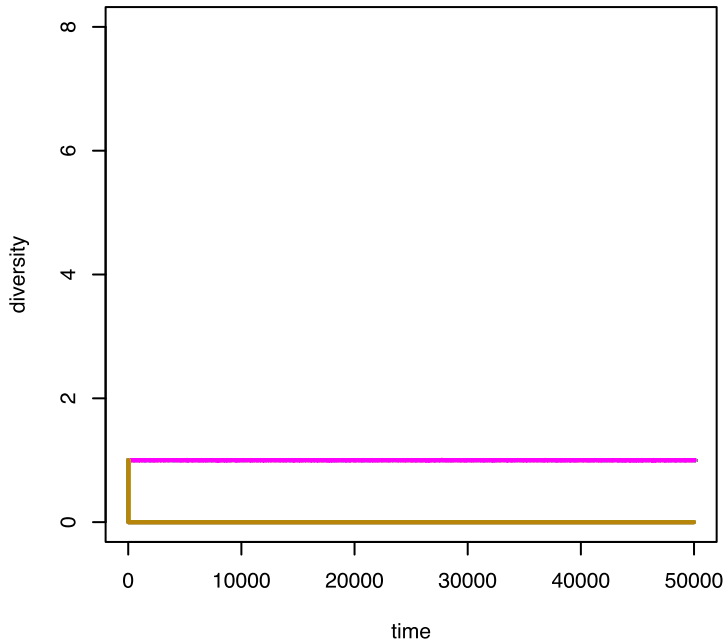

$d=0.01$

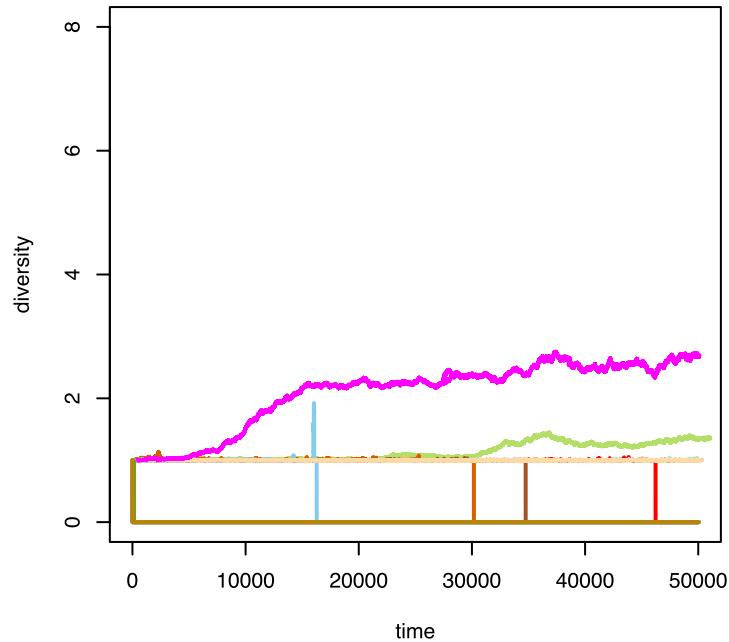

d=0.1

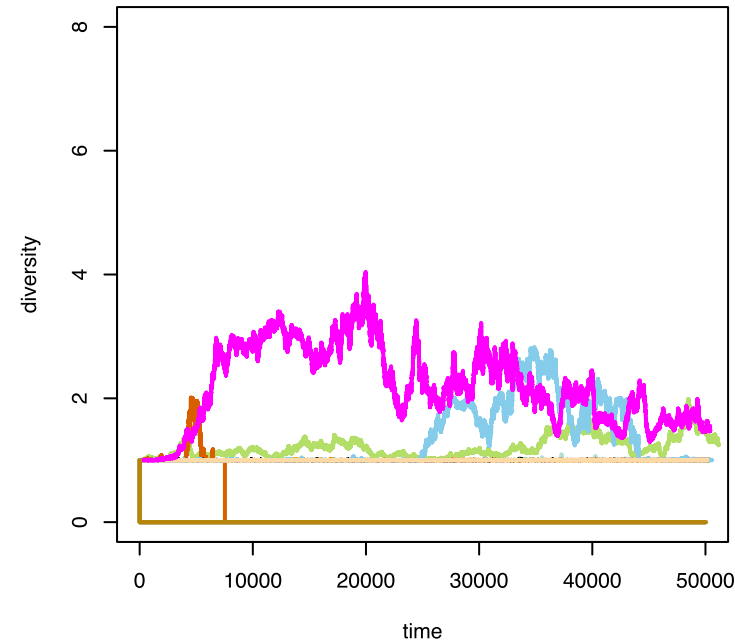
$$\Delta=10^{-5}$$
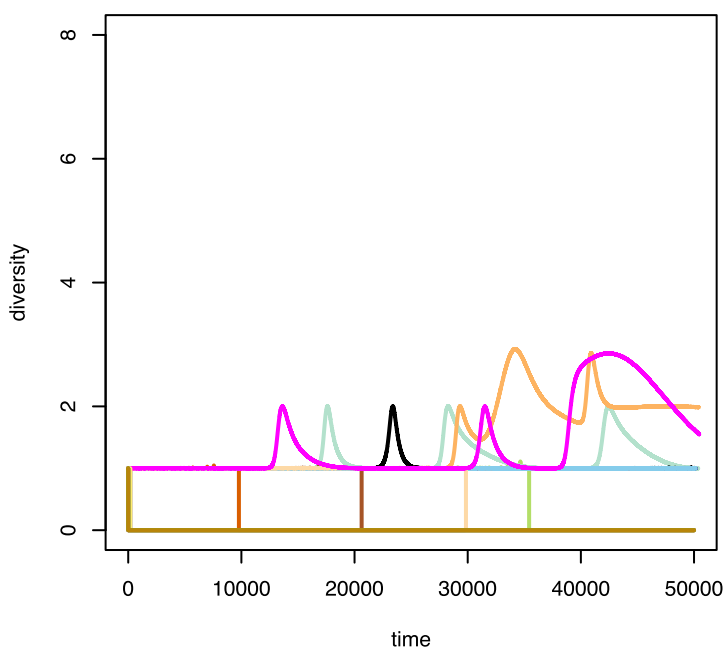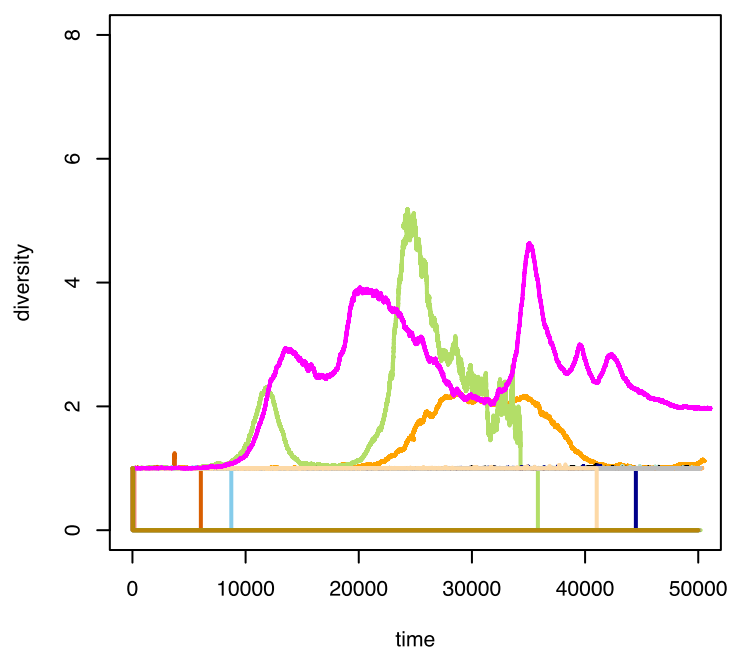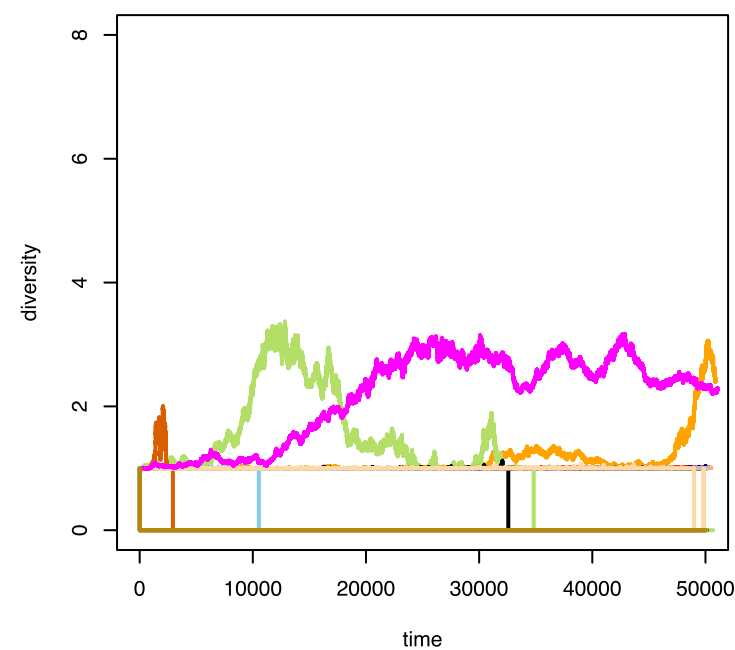
$$\Delta = 4.4 \times 10^{-4}$$
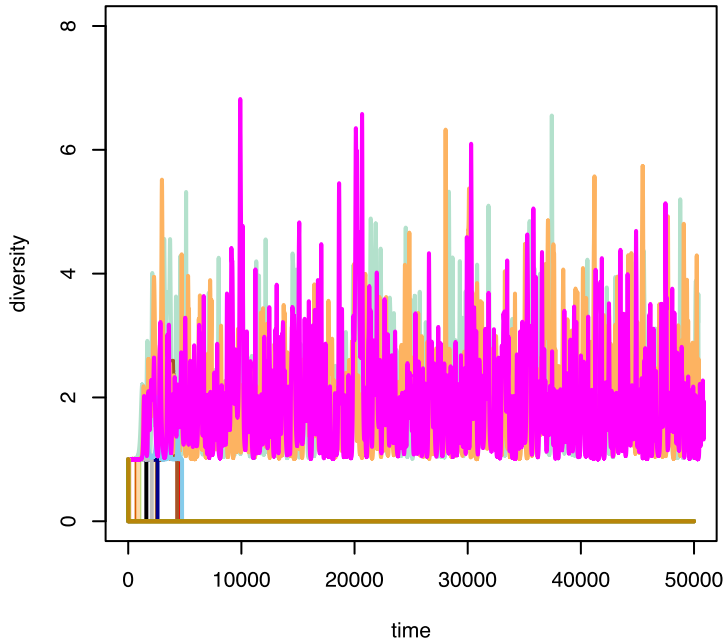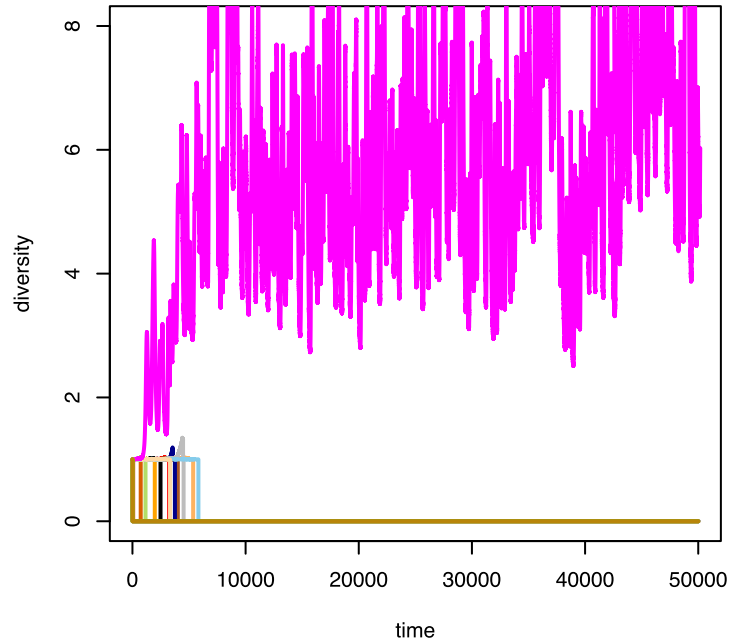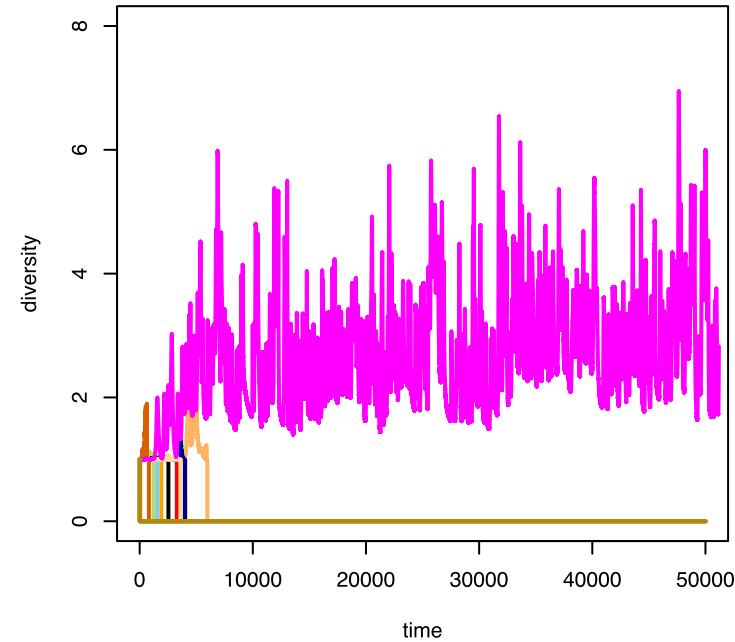

Supplement: Supplementary file 1 [file genes-11-01433-s001.zip › Figure_S9.pdf]
