# Supplementary material for: Eco-Evolutionary Feedbacks and the Maintenance of Metacommunity Diversity in a Changing Environment
Source: Genes (Basel). 2020 Nov 28;11(12):1433. doi: 10.3390/genes11121433 (PMC7761218; doi:10.3390/genes11121433)
Supplement: Supplementary file 1 [file Figure_S19.pdf › Figure_S19.pdf]

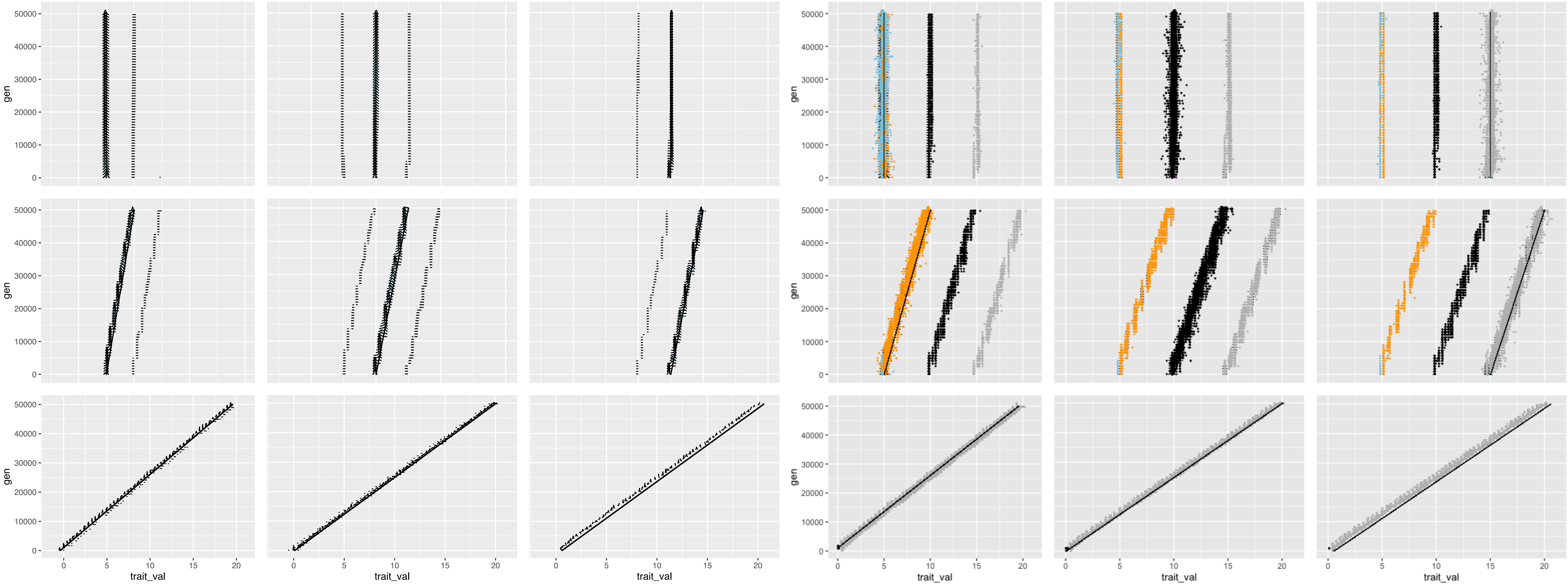

gen
trait_val
